# Supplementary material for: Whole Genome Development of Specific Alien-Chromosome Oligo (SAO) Markers for Wild Peanut Chromosomes Based on Chorus2
Source: Plants (Basel). 2025 Oct 9;14(19):3114. doi: 10.3390/plants14193114 (PMC12525989; doi:10.3390/plants14193114)
Supplement: Supplementary file 1 [file plants-14-03114-s001.zip › plants-3894958-supplementary.pdf]

Table S1 Differential loci and polymorphic markers between wild and cultivated peanuts of chromosome

Chromosome	Number of Indel Positions		Number of SNP Positions		Number of design markers	Number of specific markers	Markers polymorphism rate
	Compared to A genome	Compared to B genome	Compared to A genome	Compared to B genome			
A10chr1	2029	308	60132	3108	20	9	45.0%
A10chr2	273	708	36380	7210	20	8	40.0%
A10chr3	4086	1340	55023	13508	20	7	35.0%
A10chr4	3014	613	42113	6082	20	5	25.0%
A10chr5	3218	534	44730	6160	20	8	40.0%
A10chr6	3002	721	42077	8083	20	6	30.0%
A10chr7	2516	399	35893	4667	20	9	45.0%
A10chr8	2736	454	33288	4507	20	9	45.0%
A10chr9	2190	573	58259	5685	20	9	45.0%
A10chr10	3072	606	44637	6651	20	7	35.0%
A10	26136	6256	452532	65661	200	77	38.5%
	32392		518193				
	Compared to A genome						
A19chr1	3551		48384		104	48	46.2%
A19chr2	2652		37544		24	20	83.3%
A19chr3	4351		58382		24	15	62.5%
A19chr4	3153		45459		104	51	49.0%
A19chr5	3420		46989		24	17	70.8%
A19chr6	3200		44007		24	13	54.2%

A19chr7	2599		36835		24	15	62.5%
A19chr8	2932		34948		24	12	50.0%
A19chr9	3445		47637		24	13	54.2%
A19chr10	3273		47255		24	16	66.7%
A19	32576		447440		400	220	55.0%
	Compared to A genome	Compared to B genome	Compared to A genome	Compared to B genome			
A33chr1	4051	696	47768	7084	25	15	60.0%
A33chr2	2783	753	37697	7517	20	10	50.0%
A33chr3	5072	1636	57728	15101	20	15	75.0%
A33chr4	3463	788	45388	7260	20	13	65.0%
A33chr5	3562	654	47114	7158	20	11	55.0%
A33chr6	3999	1048	43897	9541	25	8	32.0%
A33chr7	2754	548	37002	6106	20	10	50.0%
A33chr8	3056	601	35214	5530	20	10	50.0%
A33chr9	3795	955	47406	9535	20	10	50.0%
A33chr10	3504	777	47736	8026	25	10	40.0%
A33	36039	8456	446950	82858	215	112	52.1%
	44495		529808				
	Compared to A genome						
G2chr1	109		68492		10	3	30.0%
G2chr2	62		57020		10	4	40.0%
G2chr3	122		92003		10	1	10.0%
G2chr4	94		62743		10	3	30.0%
G2chr5	86		65746		10	2	20.0%

G2chr6	80	67673	10	1	10.0%
G2chr7	86	54127	10	9	90.0%
G2chr8	85	48868	10	2	20.0%
G2chr9	93	71925	10	6	60.0%
G2chr10	92	65811	10	4	40.0%
Compared to B genome					
G2chr11	101	76744	10	2	20.0%
G2chr12	93	65410	10	1	10.0%
G2chr13	104	87527	10	1	10.0%
G2chr14	84	69161	10	3	30.0%
G2chr15	131	78050	10	3	30.0%
G2chr16	68	75198	10	5	50.0%
G2chr17	75	74167	10	7	70.0%
G2chr18	80	72255	10	4	40.0%
G2chr19	111	83179	10	3	30.0%
G2chr20	137	74162	10	5	50.0%
G2	1893	1410261	200	69	34.5%
Compared to A genome					
G3chr1	928	5163	7	3	42.8%
G3chr2	838	4635	6	4	66.7%
G3chr3	1660	8186	6	2	33.3%
G3chr4	902	5051	7	3	42.8%
G3chr5	937	5122	14	2	14.3%
G3chr6	1031	5590	7	3	42.9%

G3chr7	821	4256	11	3	27.3%
G3chr8	971	4235	7	2	28.6%
G3chr9	1031	5519	7	2	28.6%
G3chr10	934	5493	8	2	25.0%
Compared to B genome					
G3chr11	1427	8312	6	3	50.0%
G3chr12	1347	7714	9	3	33.3%
G3chr13	2059	10011	7	5	71.4%
G3chr14	1347	6969	7	2	28.6%
G3chr15	1499	8218	6	5	83.3%
G3chr16	1487	8040	6	2	33.3%
G3chr17	1270	7727	10	1	10.0%
G3chr18	1436	8070	6	4	66.7%
G3chr19	1787	9326	7	4	57.1%
G3chr20	1479	8266	7	4	57.1%
G3	25191	135903	151	59	39.1%

Table S2 Sequence Information of SAO Markers in Wild Peanut Species and Their Corresponding Chromosomal Physical Positions in the Tifrunner Genome

Markers name	Corresponding to chromosome	Left primer starting site	Left primer termination site	Left primer sequence	Right primer starting site	Right primer termination site	Right primer sequence	Marker type
A10chr1-1	1A	3951056	3951076	CTGTTTGTTTCACGTTCATGG	3951414	3951437	CGAATTTCTGCTAAAGATAACACG	IV

A10chr1-2	1A	6064238	6064263	TTGCTACCAGTCTATTACAGTCTTCC	6064728	6064747	TGTTTTCCTTCCGAGTTGCT	I
A10chr1-3	1A	9339889	9339907	ATGGCGTCTGTGACCGTAA	9340112	9340137	ACTTCAGCTCACTGTAAAATCTCAAA	IV
A10chr1-4	1A	11087159	11087184	TTATATCACTCTAAATGACCACCGTA	11087369	11087388	GGTTTCGACTTTCAGCACAA	II
A10chr1-5	1A	18702574	18702600	GACTGAGAGACATTGACTTAGAGTGAT	18702900	18702919	GCAACAACGACACCACTGTT	I
A10chr1-6	1A	36381776	36381799	AACAAACAAAGAAAGACCTGTGTG	36382055	36382077	TGGTGGAGTTTAGGTATGAGCTT	IV
A10chr1-7	1A	86914417	86914439	GCTTTCTAGTGCTGATTGTGTTC	86914776	86914797	TGAGGGATTTTAAGACATGAGG	IV
A10chr1-8	1A	101279502	101279521	GATGACGATGATGGGATTGA	101279936	101279955	TTCTTCTCCTCTGCATCGTG	I
A10chr1-9	1A	107683879	107683898	CGTTTCACGAAGACCTTTTG	107684150	107684170	TTGTTGAGGACTCCCATCTTC	IV
A10chr1-10	1A	112128351	112128377	TGCTAATAATTTCAGATTAGTTGTTGA	112128744	112128768	GAAGTATTAGATTTGTTGCAAGTGG	II
A10chr2-1	2A	3476210	3476235	AACCATCAGCGTATATACAACATACA	3476488	3476511	ACAAAAGCCAATCAATCATTAGTC	I
A10chr2-2	2A	9861145	9861171	TTTGTTTCGAAAGTTATCTAAAAGTCA	9861340	9861361	CCCTTGGGTATATTCAGAATCC	I
A10chr2-3	2A	12530035	12530057	TTCTCCTTTGAGATACTGGCTTG	12530312	12530334	CTCTGAAAGTAATCTTGGGATGC	IV

A10chr2-4	2A	34382076	34382096	AAGGGTTTGTCCGACTATGAG	34382522	34382542	GGCTAGCATCGATTTTCTTTG	IV
A10chr2-5	2A	73377920	73377945	TTAACTTTATCTAACAATGGAACCTG	73378141	73378161	GAGGTGGTGGTCAAGAAAAAG	IV
A10chr2-6	2A	88973318	88973344	GAATTTATTCGCTTTATAGTATCAGCA	88973808	88973827	GGAGAAACGGCATTAGAAGG	IV
A10chr2-7	2A	90997104	90997125	TTCGGTTTCTGTTTTTGTTTTG	90997585	90997609	AGCAATGCTTTGAATATTCTCTCTC	IV
A10chr2-8	2A	93487094	93487113	AATTTCTCCATTCGCTTTGA	93487479	93487501	GCAAATTATGAGTGTCTCTGAGC	IV
A10chr2-9	2A	95321573	95321596	AACCAAATACCAACTATGTCACAA	95322040	95322064	CACATTGACACGTACAATAATTTCT	IV
A10chr2-10	2A	100402409	100402434	GGTTCTTTAGTTAGCAAAAGTGCATA	100402588	100402609	TGAGGTCGCTATACTTTGAGGA	I
A10chr3-1	3A	340022	340048	GAGACTAGAGAGAAGACTTAAATGCTG	340477	340496	ATCGTCCCAGCAGCAATTAT	IV
A10chr3-2	3A	6607702	6607723	TGGGTTGCTAATTTTCGATGTA	6608184	6608207	CTGAGGTAATTTCTCTCCAGTGAC	IV
A10chr3-3	3A	13817765	13817784	CAACTTAGCAGCAGCAGGAG	13818086	13818105	ATGCGTTGGTGTTATTGTGC	III
A10chr3-4	3A	20012402	20012423	TGCGTCTTGTTAGTGTCTCTGT	20012731	20012750	CATGTGGGGAATTCGGTAAT	I
A10chr3-5	3A	25041773	25041792	TGGGTTGGGTGTGTGTGTAT	25042209	25042230	GGAAGGAGATGTTCATTTGTCA	I

A10chr3-6	3A	29804730	29804749	TCCGATCACAGTCATTCCAA	29805030	29805050	GCTTGTTCTCTTCCATGATCG	IV
A10chr3-7	3A	34709844	34709868	AATGTTTTATATTGGGATGTTTGGA	34710117	34710135	TCATGGGGTAACGGTCAGA	IV
A10chr3-8	3A	49166881	49166906	TGAAAATGTATATGTGTTCCTTGATG	49167292	49167317	ACCCTTATAAACCTTCTTACAGTTGG	I
A10chr3-9	3A	99133136	99133155	CGCTTCTTCTGGTTCGATTC	99133346	99133368	ACAGCCACAAACATAGTATGCAA	IV
A10chr3-10	3A	124498670	124498689	GGGTATAGCTAGGGGAAGCA	124499151	124499177	CACGCTAATATAAATTTCGTTAACATT	I
A10chr4-1	4A	526613	526635	TGGCATAGTATCCCCATACTCAC	526837	526858	GATGTACAAGCATAAGCCATCG	IV
A10chr4-2	4A	3567413	3567432	CGGGGAAGCAATCTGTAAGT	3567662	3567681	TTTGGTCGGCATGACTGTAT	I
A10chr4-3	4A	8378019	8378038	ATTGCCGGTTGAATGAAGAT	8378497	8378519	TGATGAATTTTATGGAGCCAGAT	IV
A10chr4-4	4A	12665236	12665255	AGGAGTAGAGGGGCCGAGAT	12665543	12665566	TCCAAGATCAAACATAATCGCTTT	IV
A10chr4-5	4A	21350977	21350996	TGACAAACCCAAGAGAAGGA	21351316	21351340	CCGCATACTTTAGCTTTCTATCTTC	IV
A10chr4-6	4A	38261668	38261690	TTCGCTATCTCTCTCTCTTCGTC	38261898	38261917	CAACACGTGAACCCAAACAT	IV
A10chr4-7	4A	85089667	85089690	TACAGTTGGAGTAAAGGATTTTCG	85089939	85089958	ATAGCCGATGATGGTTTTGG	IV

A10chr4-8	4A	102791572	102791595	CACTTGACGTACTTCTGAATGTGA	102791910	102791931	TCCATTCTAGAAAAGCACCTCA	IV
A10chr4-9	4A	113520621	113520647	AGAAAAGAGGACAGAACACTATTTATC	113520977	113520996	CTGAAACGAACATGTGACCA	IV
A10chr4-10	4A	120253688	120253714	AAACTAGCTGTTACACTTTTAATGCTC	120254217	120254240	TGGGGCTTAGGATAATAATTGTAG	IV
A10chr5-1	5A	6708434	6708460	AATAGAAGAATATTAGTTACGGGTTGA	6708884	6708903	ATCGTAAATGCGCAGTGTTG	IV
A10chr5-2	5A	11803930	11803949	CATCCGCCGACAAATTCTAC	11804363	11804387	CCCGTAGTAGCATTACTAAGATGGA	IV
A10chr5-3	5A	23578895	23578919	TGATGAATGTAACGAATGTAACACA	23579237	23579260	AGACTCTCACTCCTGGTTAGCATT	I
A10chr5-4	5A	28460846	28460866	ACAGTGCTTCAAAGAGGAGGA	28461089	28461111	GAGTTGTTAGCGTTATCATCGAA	IV
A10chr5-5	5A	36970969	36970994	CTTTCACCTTCTCTGTACACTAAAAA	36971188	36971207	ATGCAAGGACCCAAAAGACT	I
A10chr5-6	5A	71510536	71510558	TTTTGGTGGCTGTTTGTAACTTT	71510786	71510808	GTTGTTAGAAAATGGATGGGAGT	IV
A10chr5-7	5A	91019981	91020001	AAATTGCCATACCCTGAAATG	91020316	91020338	TTCGCTTTTAGTGAATTTGTTTG	IV
A10chr5-8	5A	100571617	100571636	GGGATGTGAGTGTGCTTGAG	100571839	100571858	TGGACGCTCGAAATAAATGA	IV
A10chr5-9	5A	103940564	103940586	TGTTAGACTAAAGGATGGGATGG	103940783	103940801	CGGAGACACCAGAGAAGGA	I

A10chr5-10	5A	111492934	111492960	AGATAGAGAGAGATGCACTACTAGCTT	111493152	111493173	TGTGGTATCTGTGATGGTTTTG	I
A10chr6-1	6A	837200	837219	TGGCAGCTTGAGTTCTATGC	837452	837474	TTGTCCATTCTCAGTCCATACAA	III
A10chr6-2	6A	7437887	7437912	CGTATTCCTCCTCTTAATCATCTCTT	7438252	7438272	TTCCCGGTATTATGATGTTGC	I
A10chr6-3	6A	11072422	11072448	CCTCTATCAAGTGCCTCATATATACTT	11072895	11072921	ATTCACATTAATGATAGTCATCATAGC	IV
A10chr6-4	6A	17374026	17374050	TGAGCTGCTTATTCTCTTTATTTGG	17374359	17374378	GGATAATGGTAGCGGTGGTG	IV
A10chr6-5	6A	42287580	42287600	GGAAATTGCATTGTCATTGAG	42287926	42287949	GCTTAAATTTTGCTGTTCTCTGAA	IV
A10chr6-6	6A	81002608	81002626	GACGATCATGACCGCACTT	81002939	81002959	GGAGGTCACGATTAATGGAAG	IV
A10chr6-7	6A	98913486	98913509	GAATAGTGAGTTGTTTGTGTTTGG	98913824	98913844	CCCCAAAATGCTTAATTTTTC	IV
A10chr6-8	6A	105930842	105930864	TCTTCGTGCAAGGAGTAATTTTT	105931041	105931065	AGAAAGTATCACTCGTCACTGAAAA	IV
A10chr6-9	6A	110337880	110337904	TGGAGAGTGACAGAGAGTTGATATG	110338171	110338194	GAAAATAAAGGGGAATGATTGAAA	IV
A10chr6-10	6A	115726558	115726578	CCTGGACATACATGGATTTGA	115726913	115726932	TGTGAAAGCTTGGCATACCT	I
A10chr7-1	7A	5672075	5672094	AATTCGGCGATTAAAATCAA	5672270	5672289	AATGTATTGGCCAAAATGGA	I

A10chr7-2	7A	10438471	10438496	CAGCAGTGAATGATATATAGTCTTGG	10438806	10438825	TGAGTGCAGCAAAGTCAAGA	IV
A10chr7-3	7A	22250687	22250706	ACGTGCATGCATAAGACCAT	22250969	22250990	TCCATTCTTGTTGACTCCCTAA	IV
A10chr7-4	7A	35046987	35047006	TATAAGTGAGGGCGGGAAAG	35047195	35047217	TGGTTAGTTTGAGGAATGTACCA	I
A10chr7-5	7A	53880917	53880937	CCAAAGCCTATTGTGGTTGAA	53881423	53881446	GCAGTGAAAATACAAGAACGAAGA	IV
A10chr7-6	7A	59788556	59788577	TCAATCCATGTATGGCTCTAAA	59788849	59788869	AAATAACCCACTCCACCAAAA	I
A10chr7-7	7A	62101525	62101546	TGTGCATTCCATCTACAATTCC	62101991	62102011	TGGACTATGGGCTTAATGTGC	IV
A10chr7-8	7A	72423267	72423286	TCCACGTATTTTCCCGAGAC	72423728	72423748	CTCCAAAACCTAACTCCCACA	I
A10chr7-9	7A	78037289	78037309	TCACAATTAGACCCATGTGGA	78037647	78037671	CTGTATCGAATTCTAATCAACATCC	IV
A10chr7-10	7A	80041220	80041240	GCTGTTAGGTTAGGGGTTTGG	80041516	80041538	TTGTGTGATTCTTAATTGCTGGA	IV
A10chr8-1	8A	131740	131766	ATCTTAAAAGACCAAACTAGAATTACG	132097	132119	TTGTACACTTTTAACAGGGCTGA	I
A10chr8-2	8A	7191114	7191135	TCTAACCCTAGGACCAAAATCA	7191396	7191421	CATACAATTAAACAATGAATCAAGCA	IV
A10chr8-3	8A	11718684	11718706	TTTTTGTTAATCTCCAACGGTTA	11719005	11719027	TGGCTTTAAACTTACCCTCTTTG	I

A10chr8-4	8A	17253686	17253709	CAACACTCAGATAGAAACCATTGC	17254071	17254090	TTCTCCTTCTCCAGCACGTT	I
A10chr8-5	8A	25208498	25208524	AAAAAGAAGAAGAGAAAATCAAAGTCA	25208892	25208916	TCCATGTTTATCCAATTATCCATTT	IV
A10chr8-6	8A	31301262	31301286	TGTGGTGGTACTTGGTAGAGTATTG	31301571	31301593	TCACACCATTCTGAGATATACGC	I
A10chr8-7	8A	34699589	34699612	TTGACACTCAACGTGTTACTTGAT	34700064	34700085	GATCACTTGAAGGAGCTTGGTT	I
A10chr8-8	8A	40065724	40065750	TCTCTTCATTCTTTTATAGCCTGAAAT	40066021	40066042	ATCACACCATAATTCGTTTGGA	I
A10chr8-9	8A	44551914	44551933	TCCACTCAACCCAAGATTCC	44552168	44552187	GATGAAAACAACGACGATGG	IV
A10chr8-10	8A	51157195	51157217	CACAACACGGTAACTTCATTTCA	51157506	51157529	GGATTCATGACCTCTTAGCATTTT	IV
A10chr9-1	9A	692846	692872	CAAGAGTAAAGTAAACAATGGTAGGTG	693039	693065	TTATTAACACAAGAACGTACGATTAAA	I
A10chr9-2	9A	2959623	2959643	CCACAAGCCAGTGCTACTGTT	2959853	2959876	TGAATTATTGTTGTTTTGGGATTC	IV
A10chr9-3	9A	5640506	5640530	TTAAGAGGAATTGAAGATGTTAGGA	5640847	5640870	TTTATTGTGATTTGATGCATACCT	I
A10chr9-4	9A	9672469	9672492	TGATGGTGTAACTGAATGTTTCAA	9672822	9672842	CTGGATGATTCAATGGCCTAA	III
A10chr9-5	9A	12663811	12663831	CCAACAACACCACCAAGTTCT	12664100	12664122	TGCGCTCTTATAAGTGTCAGAAG	IV

A10chr9-6	9A	16429065	16429091	GTGAATTCCTTATCTCCTTAATTATCA	16429305	16429325	CAAATTCACTCCTCATCAACC	I
A10chr9-7	9A	81232378	81232397	GAAAGAATGCTCACGCATCA	81232603	81232622	ATGAAGGTTCTGGGGAAACA	IV
A10chr9-8	9A	103989381	103989403	TGTGTATGTTCGTTCCTAGCTGA	103989578	103989603	ATCTCTGTATTTTGACGTAAGTTCCT	IV
A10chr9-9	9A	111234505	111234524	CCTGTTGCACGAACAAGAAT	111234707	111234726	TGGTCTCGACGATACGAAAG	IV
A10chr9-10	9A	119835563	119835587	GGAGTATTGTTTAATTTGTCGACCT	119835841	119835863	CCCAAAATCACCTTAAGACAGTG	IV
A10chr10-1	10A	1869143	1869165	GCATCCTCATCTACACAACACAA	1869402	1869425	AACACAAGAAATAGCCATACACCA	IV
A10chr10-2	10A	7344687	7344712	CAGAAATTGGACTAACTGATTTGTAT	7345125	7345145	GAACGACTTCTGAGAGCGAAT	IV
A10chr10-3	10A	9698168	9698190	CAACACACTCAGAATATGGGAAT	9698560	9698579	ACCACCGAGCAGATCATGTA	IV
A10chr10-4	10A	14468108	14468134	CAAGGATGCTCACTTATTTATTTATGA	14468435	14468460	TTCAATTTTGCACTTAGAAAATAACA	IV
A10chr10-5	10A	23814778	23814799	CCATTCTTTCCAAAATGACACA	23815184	23815207	TTTGAGCTTATATCTCGTCCAAAA	IV
A10chr10-6	10A	32074633	32074655	CCTAATCCTCTAACTTGCCACCT	32075007	32075027	CAAGGCTAGAACGCAGAGAAA	I
A10chr10-7	10A	80064613	80064633	GATCCGCTGAACATCTATCCA	80064916	80064939	TCAATCACACCAAAACATTATTCA	IV

A10chr10-8	10A	95015876	95015894	CGTCGTCGTTTGGAGAAGA	95016138	95016159	TTCCAGTCGAAAAACTCATTTG	II
A10chr10-9	10A	101549575	101549596	CCTTGGTGTGTTAACAGGAAAC	101549907	101549926	ATGGACGCCTATTCCAGCTA	III
A10chr10-10	10A	112825324	112825345	AAGGTTGGTAGGTCTTCGCTTA	112825618	112825642	TGCACACAATGAAAATATCTAGAGG	IV
A10chr11-1	1B	3383585	3383605	CCAGAACAGGTAGGACACTCA	3383791	3383810	TTAACCTGTTGGATGGAGGA	IV
A10chr11-2	1B	12279535	12279559	CAGGCATGTCAAACTTTAGTTATTC	12279737	12279757	CCTTCTCCTTAAGCTCCCTTT	I
A10chr11-3	1B	12856996	12857016	GGATGAAATCATGCAATTGTT	12857302	12857321	GGAGGTAGTGGAGGGACGTA	IV
A10chr11-4	1B	17984860	17984879	ATAGAGGCAGGGCAGTCAAA	17985178	17985199	AAAGTGTTTTGCCATTCCTGTT	IV
A10chr11-5	1B	18015783	18015809	TGACAACATACATAACAATAACTTTGA	18015985	18016004	TGTGCATTGAGGATTTGGTT	III
A10chr11-6	1B	53481520	53481542	TCAATAGAGGAGAAATGGGATCA	53481957	53481976	CGAAGGATCGAATCCAGAAG	IV
A10chr11-7	1B	120035035	120035061	TTCAACAACTTTAAAATCTCCATTATT	120035434	120035455	TTGTTTTCACTAGAGGCACAAA	IV
A10chr11-8	1B	125189189	125189212	TGTTCACGTATTTTGGACATTTTT	125189663	125189685	ACACCAGATGGTACATGAACTTG	IV
A10chr11-9	1B	125515802	125515827	TTCTACTGAATACCACCTCTACTGAA	125516058	125516080	ACTGAACTTTGATATCGTTTTCG	I

A10chr11-10	1B	109109457	109109482	GACGTAACAACTATCACTTCCTTTCA	109109739	109109762	CAAATATGTCAAGTGTCACAACCA	I
A10chr12-1	2B	3162703	3162720	CCGGGAGCCAAATGTTTC	3162908	3162932	TCAAGAAAGATTTCATTTGATTGTG	IV
A10chr12-2	2B	5384361	5384385	TCTTAATGAACCGTATGAAACTTTG	5384555	5384577	TTTCAAATTCACTTCCATTACGA	II
A10chr12-3	2B	11064291	11064310	ATTGTGTGGGTGGAGTTTCG	11064486	11064505	CATGGTATTTGATGCCGTTG	IV
A10chr12-4	2B	22649889	22649913	TCATTATATGTGTGAATGACCAACT	22650149	22650174	TGAATATTGAGGTTTAGATTTAGCAA	IV
A10chr12-5	2B	90920525	90920544	ACAGCAGGATAGCAGGACAG	90920756	90920780	TGTTGTAACCATGCTAAGATCAAAA	I
A10chr12-6	2B	104289449	104289469	GCTGCGTGTTGTGTTATCTGT	104289685	104289708	GAGAAGAGATGCACAAGAGATAGG	III
A10chr12-7	2B	112492265	112492290	GGACAATATTCAGATACATTCAAACA	112492472	112492498	TTCCTCTCAACTTGTGAATAAATCTAA	I
A10chr12-8	2B	113911328	113911350	AAGAAATGTCTTGGAATTGAAGC	113911761	113911787	TCAACAATTACTAATAGGGATTCACTT	IV
A10chr12-9	2B	115194211	115194237	TCTATTTGATGATGAACAAAATACTGA	115194421	115194446	AATTAATTGCTTCTTGTGGTTACATT	I
A10chr12-10	2B	115910751	115910773	CCAACACCTCTAAAACACTCACC	115911029	115911049	CCGATTAACACTGTTGGCAAT	IV
A10chr13-1	3B	5997886	5997910	TTAACTATGCTCATTTTGTGAATGT	5998157	5998180	CAGAAATGGATACTTCGAAATCAA	IV

A10chr13-2	3B	11776140	11776161	GGAAAGGAAATTCTCAGAGAGG	11776450	11776468	CGATTATGGGACTGGCAAT	IV
A10chr13-3	3B	16093895	16093920	AATCAATCATATATACTTGCGTGTCT	16094136	16094160	GGGTAGTTCATAGTTGTTTAATTCG	III
A10chr13-4	3B	83054506	83054525	AGCTCACACATGAGGCAAAC	83054808	83054829	TCCATTCAGATTTCAGAAGCTC	IV
A10chr13-5	3B	131041960	131041984	TTAATTAATCCCAAGATTTGCTTAG	131042161	131042180	CATTCTCGCCCTCAAGAGTT	IV
A10chr13-6	3B	136734714	136734738	CAACACATACATTGTTCTCGTTGTA	136735105	136735131	TCTTTACAATTTACAAAAATCAACAAG	IV
A10chr13-7	3B	139811540	139811565	TCAATACAGGCTAAGTGTATGTTTCT	139811977	139811998	CTCATTAGTTCCTGCAGGTGTC	IV
A10chr13-8	3B	140364119	140364138	CCATGCCCGTAAACAAACTT	140364362	140364382	AGCAACCAAAATCCAATGTTT	IV
A10chr13-9	3B	141228347	141228365	TCCTTCGCGTCCAAGAAGT	141228776	141228795	CATCCTCATCACTTGCATGG	IV
A10chr13-10	3B	142269034	142269054	CAGAGAGACGCGGAGTACAGT	142269661	142269682	GCTTACAAAACTGGGAAAAGGA	III
A10chr14-1	4B	750096	750117	CATACTATGGGAGATTGGATGG	750356	750374	AATCGGAATCGGAGAATGG	IV
A10chr14-2	4B	4639343	4639369	TGTGTTCTGTTAGTTAGCCTTATTGAA	4639566	4639586	TGATTGCCTGTTTCAGATTCA	IV
A10chr14-3	4B	10426594	10426611	GACCCAGTGGCGAAGAAG	10426911	10426930	GGCGGAGGAAAGTTATACCC	IV

A10chr14-4	4B	109312213	109312232	GGGGTGGGTTTAGAGTTAGC	109312484	109312503	CGTCAAACTCTTCCGCTTCT	IV
A10chr14-5	4B	115985387	115985408	CCTTTGTATCCTCCCTCTCTCT	115985623	115985643	TGCGGTTGGGACTATTAATTT	I
A10chr14-6	4B	124361331	124361357	AAAGATCTATTATTCAAGAGTCGCATA	124361538	124361559	TCCCATACGGTTTGAAGATATG	IV
A10chr14-7	4B	129077583	129077602	TCATGTCGCACTGGAAATTG	129077930	129077952	AATCTTCTTGTTATGTGGGTGCT	I
A10chr14-8	4B	130557547	130557566	CTGCGTCTGATGTACCTGGA	130557762	130557781	CACGCACAATCCATCACATA	I
A10chr14-9	4B	134418710	134418735	TTCAGTTAGTTACATCGGCTAGAGTC	134419016	134419041	TGACGTATCGATAACATATTCTTGTG	IV
A10chr14-10	4B	139466861	139466880	TCAGCACTCAACACTCAGCA	139467293	139467313	GGATTGGTTATTCGAATGCAG	II
A10chr15-1	5B	9182277	9182299	GCAGGTTGAATTTGTATTTGAGG	9182588	9182607	CTTCACGATCACATTGAGCA	I
A10chr15-2	5B	10112281	10112302	CCTTCGATTTTGGATCATCATA	10112691	10112715	TTCTAAATCGGATTAACATATTGGA	IV
A10chr15-3	5B	13445955	13445975	GCTTTGGGTCTAGAGGTTTTG	13446190	13446209	ATCATCCAAACGCAAATTCC	IV
A10chr15-4	5B	17818562	17818581	GCGAATTCCATCTCCTGTTC	17818818	17818837	TTCTCCGACGGTGTTCTCTT	I
A10chr15-5	5B	25489938	25489962	GAAATCCGTGTTTTGTATAATCCTT	25490158	25490177	AGACAACAATGCCTTGGCTA	I

A10chr15-6	5B	26762773	26762796	GCACACTCTAGATTTCAGGTCTCA	26763111	26763132	GCATGTTCCTAGTGAGCTTCAG	IV
A10chr15-7	5B	45695893	45695917	GCTTGCTTTGATTGAAATTCATACT	45696296	45696319	AACCGACATTTACAGACAAAGACA	I
A10chr15-8	5B	75987651	75987676	TGCATGATTACCTAAGTAGTGTTTGA	75988069	75988094	CTTGCCTTTATTTTGAGTGTAATAGG	IV
A10chr15-9	5B	132934533	132934559	TACTGTGCTATAAACAGAGAAATATGG	132934846	132934865	TGAGCTGGCTTGTGTTTGTT	IV
A10chr15-10	5B	133517321	133517347	CAGCAAAGATCTAGATTATTATTCCAA	133517533	133517557	TGACACAAGTAAAAGCATATCATCC	IV
A10chr16-1	6B	6710089	6710109	CAGTGTTCATGAGGCCTTAAA	6710288	6710307	GTCGAAATTTTCCGGTGAAG	IV
A10chr16-2	6B	11412628	11412654	GCTCATGGAAAATGTAATAAGAGTCTA	11413061	11413083	TGGGGGATGTACTATATGAAAGG	IV
A10chr16-3	6B	15355474	15355496	GAGAGTTCATGATCCATCCAGTT	15355835	15355853	GACAACTGGGAGTGGGAAA	III
A10chr16-4	6B	44498019	44498038	GAGGCTCAGAGACCAACTCA	44498276	44498298	CGAGATTTTCATAGATAGCACCA	IV
A10chr16-5	6B	51184825	51184845	TCGTCGAAAGAGAGAGAGAGG	51185118	51185137	GTGCCATATTCCTGTGGTTG	III
A10chr16-6	6B	99369232	99369255	GCCTTGGATATATCACTACCCACT	99369569	99369590	GGAAATGAGGATACTCGTAGGG	I
A10chr16-7	6B	124482142	124482168	GCAAGAATGGTAGATATATAAGTCCTC	124482356	124482377	TCAGAAGGTATGCTTTCATGTG	IV

A10chr16-8	6B	129377487	129377510	TTTATCACAATTCTGTTGGGTAGC	129377933	129377953	TGTTCATACGATGAAGGCTCA	IV
A10chr16-9	6B	136364989	136365015	TGGAGATTATTACTAGTTCTGGCTACA	136365235	136365255	AACTCTCGGAGTTTCAAGTCG	IV
A10chr16-10	6B	142918074	142918096	TGGGTATGTGTTGATCAGTATGG	142918349	142918375	ACTTATGATTTGAGAGTATGTTCTTGC	IV
A10chr17-1	7B	441704	441723	CCCCTTGGAGGATGTGTAAT	442194	442216	TGAAGCTCTCACTGTGGAGTAAA	I
A10chr17-2	7B	1544455	1544474	GCATGAGCCATGAGTCTTCA	1544693	1544716	CAAGGGATAAAATGAACAGAAAAA	IV
A10chr17-3	7B	4480692	4480712	TGCCCAAGGTATTAGGCTTTT	4481073	4481099	TCAATGTTCGAATAAATACTATCCCTA	I
A10chr17-4	7B	18056410	18056430	TCAACAGTCGTCAGTCGACAC	18056834	18056855	GAAAGACAACAATCAGCCAAAA	I
A10chr17-5	7B	23184677	23184703	AATATTCGAAAAGTACATACCTTCCTT	23184919	23184938	GAATTGTCGGTTGATGTTGG	I
A10chr17-6	7B	40362902	40362925	CAGGAATCATAACTCCAATAGCAA	40363219	40363238	GCGACTTGTTATGGGAGGAG	I
A10chr17-7	7B	122254784	122254807	TTTCTAAAGTTAATTTCCGTCCTG	122255243	122255265	CTCTAAATCAAGCATCAAACCTG	IV
A10chr17-8	7B	124265271	124265294	TCTCTTAGCAGCTTCTTCAATCAA	124265513	124265532	AGGTGTGCCTTGTGGAGTTT	IV
A10chr17-9	7B	128355257	128355276	TGCATCACAACAACACTTGC	128355466	128355490	CACAATATAACGAAGAAGGTTTCCT	IV

A10chr17-10	7B	131641786	131641810	CGGTCTTCAAAGTATCACTTCACTT	131641967	131641992	GGTCATTACCTATTAGTGATGTTTGC	IV
A10chr18-1	8B	6576798	6576817	TTACCACCCGCCATAACTTC	6577122	6577141	AGGACAGGCAGTGGACAAGT	II
A10chr18-2	8B	7393679	7393699	TCGTTTGTTCTTGCACCTACT	7394021	7394043	CACCCTTGTTGGTTAAATAAAGG	I
A10chr18-3	8B	17854836	17854858	CGCAAAATGAATAATATCCACAA	17855174	17855193	CGGATTGAATCACATCGAAT	IV
A10chr18-4	8B	23506419	23506440	AAGAGAATATGGACCCCAATGA	23506643	23506662	TGACCACACTTTTCCTTCCA	IV
A10chr18-5	8B	30034539	30034564	GAAAGCTAAGATGTTGATCACTTTTG	30034966	30034985	GCGATGTAAAATGCACAAGC	IV
A10chr18-6	8B	34324932	34324957	AAAATTAATTGGCACTTGTTTTAACC	34325246	34325265	TGAGGATCCACTGGCATAGA	IV
A10chr18-7	8B	97118027	97118047	TCAACTCCCTTGATGTGTGTG	97118341	97118364	CATGAGCTTACACCAATAAATGCT	IV
A10chr18-8	8B	102275212	102275236	TGTGTCACAACTATAAGCATTTTCC	102275512	102275536	CCTAAATGTTTAAGGTTTGGGATAA	IV
A10chr18-9	8B	119313019	119313041	TGAGGCTTTACAACAAGGTGTTT	119313310	119313332	CATCAATGTATGTTGTCCAGACC	IV
A10chr18-10	8B	128181947	128181968	TCTCTTCTCAACAAACCCAAAA	128182261	128182285	TTGGATTGTAGCTATGTGTAGTGTG	III
A10chr19-1	9B	6608148	6608167	CACACTTGTGCCTGACCTGT	6608416	6608435	AAGTCTTTGAGTGGGGATGG	IV

A10chr19-2	9B	8266512	8266531	TGTGGGAAGAGGAAAGCCTA	8266706	8266727	GGGGTGGTACTAAGTTTCTCCA	IV
A10chr19-3	9B	14974751	14974777	CAAATTAAAACATTCCACACTATCTCA	14974973	14974992	GAGAACGTGGCTGCTATTGA	IV
A10chr19-4	9B	18878564	18878585	CTTGCAACGAACTACTTTCCTC	18878824	18878846	TTTCCTTTGCACATAAACACTTG	IV
A10chr19-5	9B	21443189	21443208	TTGTCCTGGGCAGGTAGAAC	21443430	21443449	GATGCCACATCAATGTCGAG	I
A10chr19-6	9B	26719185	26719204	TGCCTTGCCCTATGACTTCT	26719601	26719621	TGTGAATTTGTGGACCATGAG	IV
A10chr19-7	9B	97719253	97719272	ACGAGCTTATGCTTGTCCAT	97719516	97719538	AGCAGGAGAAACAACAGTAGATG	I
A10chr19-8	9B	98219009	98219028	ACGCATCAACACGAACAAGT	98219246	98219271	TTTACATGCAGTAGATGTGGAAATTA	I
A10chr19-9	9B	98853760	98853778	GCTACGCAACGTGGTTCAC	98854004	98854026	ACGAGGTCATGAAGAAGAATTGA	III
A10chr19-10	9B	120456514	120456538	TGTGTTAGAATTTTGATATTGACCA	120456812	120456833	CGATTCTCTTTTCGATCTTGTT	I
A10chr20-1	10B	651904	651924	AGTCCCAATCTCAGTGACTCG	652304	652323	CTCAAGCTAGCTGGCGATTT	I
A10chr20-2	10B	4262523	4262542	ATCCATCCATCCATGACCAG	4262770	4262789	GGAACCACCGTAATTCTTCG	IV
A10chr20-3	10B	13987725	13987748	TCACTGTCTCACTCCATAGAATTG	13987970	13987996	TGAATTGTGAAGTATTTAGAAGCAAA	II

A10chr20-4	10B	105643895	105643919	GGCCTTGAAATGTTTATATATAGGG	105644118	105644137	GGGACACAATGAAAGCTTGA	IV
A10chr20-5	10B	112537181	112537202	TTCCTTGTTTTTCAAAACTCCT	112537653	112537673	CAAGGGGGATAGTTTGTTTTG	IV
A10chr20-6	10B	121723252	121723271	GCTATTCATGCCTCAAACCA	121723532	121723556	AGTGAATGAGATGAAGATTGATTTG	I
A10chr20-7	10B	126378606	126378627	GATGCATTTTGGATTCATTCAC	126378820	126378845	TGCTCTTATGCTAACGTTTAGTGTTT	IV
A10chr20-8	10B	133545262	133545283	CTCAGGTACTGATTTGGTGCTG	133545484	133545508	TGTTTCCCAGAGTAACTATCGATCT	IV
A10chr20-9	10B	138004372	138004393	CTCCACATCTACACCTTCCTCA	138004812	138004831	ACATGCACAGGAGGATGATG	III
A10chr20-10	10B	139966590	139966616	ACACATATTACAAATAGAATGCAACAG	139966930	139966949	AATGCAGTGCAGGATTGTTG	IV
A19chr1-1.1	1A	1073168	1073194	AAGTACTCTTCAGACTGTTATGGAAGC	1073445	1073468	TGAGATTTACAAAACATGCTACCC	I
A19chr1-1.2	1A	1073181	1073200	ACTGCATGCCAGTGTTATGG	1073445	1073468	TGAGATTTACAAAACATGCTACCC	IV
A19chr1-2.1	1A	109428349	109428373	CAGGGTATTTAGTCAACCATATTCA	109428684	109428705	TCACCACTTGAGCAATTACCAT	II
A19chr1-2.2	1A	109428351	109428373	GGGTATTTAGTCAACCATGACAC	109428684	109428705	TCACCACTTGAGCAATTACCAT	IV
A19chr1-3	1A	475789	475809	CCAATCCAATGCAAGTTTGTT	475997	476016	TGGGATGAATAAACGTGCAA	IV

A19chr1-4	1A	1153116	1153136	TCAGGATCAAACCAAACATCA	1153383	1153405	TTCACGCATATCCGAAAATATAA	IV
A19chr1-5	1A	1366923	1366942	TGTTGTGTTGTGGTGAGTGG	1367194	1367215	AGCCAGTAACAAAGAAATGCAG	IV
A19chr1-6	1A	1400458	1400476	GGGGGTGGTGAGATCTGTT	1400688	1400714	TTGGATCTTTATTCTCTCAATACTTCT	IV
A19chr1-7	1A	1463058	1463084	TTAAGTATGCTACTAAAACCTCCCTCA	1463346	1463367	AGTTGAAGCCAGTGACAGTTCA	III
A19chr1-8	1A	2445098	2445117	CCCACCATTCATTACCATTC	2445461	2445486	TCATTTAAGCTCTATTAGATTGGTGA	I
A19chr1-9	1A	3013859	3013880	GGACAGCAATAACCAATAAGGA	3014181	3014203	GACTCAGACATATTCCCTTCTGG	IV
A19chr1-10	1A	3555204	3555223	AGCCCAAACTTCACCAAAAG	3555651	3555670	TTTGGGATGGGTTCGAGTAT	IV
A19chr1-11	1A	3951056	3951076	CTGTTTGTTTCACGTTCATGG	3951414	3951437	CGAATTTCTGCTAAAGATAACACG	IV
A19chr1-12	1A	3960271	3960294	GAGACTTCATTCTCTCCACTGTGA	3960520	3960541	TTCAAAATGCAACTTCCTTCAA	I
A19chr1-13	1A	4981231	4981250	GCCTAGTTGGGACTGTGCTT	4981474	4981493	CGCAATATGAATGGACGTGT	IV
A19chr1-14	1A	5053409	5053434	CACAACTAACCTTCTTTACTTCCTCA	5053890	5053911	CAGATTTCAGCCCAATGTTAGA	I
A19chr1-15	1A	5350312	5350332	AGCCATGTCATTTCTGTAGCC	5350573	5350594	GCTGTAGGTAGTGATAGGTTCCTG	IV

A19chr1-16	1A	5959705	5959723	GTAGGACCGGAGTGCATGA	5960002	5960023	CACACTTCAGCATGCTACACAA	I
A19chr1-17	1A	7427884	7427905	TGCGTGCAATTCTGTTTTATTT	7428342	7428361	ATCGGTTACCCACAAACACC	I
A19chr1-18	1A	7845912	7845934	CAAGTGCTGGTGAATAACTAACG	7846329	7846351	AAACAGTCGCTAATAATCCGTTG	I
A19chr1-19	1A	8097936	8097956	TGATGAGCGATATCAATTCCA	8098210	8098231	AAAGCGATTGTACGAATTTTCA	I
A19chr1-20	1A	8743065	8743084	CCTTGAAAACAGCCTTGAAA	8743351	8743371	GCAATCTACATAGGGCTGCTT	IV
A19chr1-21	1A	8977896	8977922	ATAAATAGTTGCTAAGAGTGATTGCAT	8978132	8978158	TTGACCTAATTACTATCATGTATGCAG	IV
A19chr1-22	1A	9953440	9953466	CAACGATAGATATTTTCTAACTCGACA	9953712	9953738	GAATAGGAGTGAAAGTGAAATTAAGGT	IV
A19chr1-23	1A	10025298	10025319	TCACATCAGATCAGTTCAGACC	10025541	10025560	CCCGAGACGGATTAGTCATT	I
A19chr1-24	1A	10126979	10126999	ACTTCTTTTTCTTGCGACCTT	10127274	10127294	TCAAAACTGGTCATTGGTTCA	I
A19chr1-25	1A	10820080	10820102	TTAATTAATGCCACTCTCGACAA	10820545	10820564	CCGATTTGAGGAACAAGTCA	I
A19chr1-26	1A	12488083	12488109	TGGTAAAACTAACTGAGAATATGTTGA	12488293	12488312	GGAAATGTGATTGCGAATTG	IV
A19chr1-27	1A	12922653	12922673	TGTTGTTGTAGTAGGCCCTGA	12923073	12923096	TCTGAAACTCGATTTAGAAGACCA	IV

A19chr1-28	1A	13420774	13420796	CTCTCATCAGTCTCATCAGTTGG	13421267	13421287	TGGGATTCGTAGATGAGGAAG	IV
A19chr1-29	1A	14820848	14820873	AAGAGTTGTTCAGCTCTACTTCCTTT	14821065	14821089	CGACAGACAAGTAGTGGTAATTGAA	I
A19chr1-30	1A	15771319	15771345	CAACACATACATTACACCAAACTAACA	15771610	15771629	CTCCCCAATCATTCCTGAAA	IV
A19chr1-31	1A	17269694	17269714	CTTCCACACACACACACACAC	17270089	17270114	GGGTAAATTTTCAACTTAGATTATGC	IV
A19chr1-32	1A	18305025	18305045	TCTCAGGGTTGTCTCCTTTCA	18305225	18305246	CCAAAACCTAGAAAGAGGTTGG	IV
A19chr1-33	1A	19950370	19950393	TTCCTAGTTTTCTTTCCTCTTCCA	19950791	19950810	TGTCTCATGGCGAGTTTCAG	I
A19chr1-34	1A	21959659	21959685	TCGATCCCTTATTACTATTATCTATGC	21959897	21959922	CAAGTCAATCTCCAGATATCTTCTTA	I
A19chr1-35	1A	22274919	22274942	GAAGGTACAACGACATTACACAAT	22275217	22275238	ATGCCCCTGAAGTAACTTAGAA	IV
A19chr1-36	1A	22493261	22493284	GCAGACAGTCACTAACAAAAGTCA	22493500	22493519	TGATATGGAGCGTTTTGAGG	IV
A19chr1-37	1A	23006634	23006658	CAACTGAATATATCCTGACACCAAA	23006906	23006931	GCATATTTAACTCTGAAAACTTACGC	I
A19chr1-38	1A	23887649	23887671	CGTGTGGTATTTTATGTTCTGTG	23888105	23888124	AGCATTACCGTGAGCCCTAA	I
A19chr1-39	1A	24486323	24486342	CCATTCGCTAGCATGACACT	24486774	24486793	TCGTCTCGTCCTCACAATCT	IV

A19chr1-40	1A	27539675	27539695	CCATCCCGTTAATTAATTGCT	27539924	27539950	GGTTAGATTTGATAACTCTTTTATTCG	IV
A19chr1-41	1A	29708154	29708173	CTGCACAACACTGCAAAATG	29708442	29708468	GATTAGTTTGTAAAACAAGGAGTTCAA	I
A19chr1-42	1A	30125156	30125175	GAATGGAAGGGGGAAGAAAG	30125378	30125404	AATGCTAAGACAAATCCAAAATAATAA	I
A19chr1-43	1A	31539444	31539470	CACAAAATACTAAAGTGTTCAAAACAA	31539871	31539890	TAAGGTCGGGATCTTGAAGG	IV
A19chr1-44	1A	31773450	31773473	CATTTCACTTTCTGTCTTCTTCCA	31773874	31773900	TTGATAACTATGATTCCAAGAGTATGC	I
A19chr1-45	1A	31873824	31873846	CCTGAACATTAAATGGTGTGATG	31874184	31874205	AGAGAGTGAGCATTGAAAGCAA	I
A19chr1-46	1A	32728454	32728477	TTCAGAAACCAACATAACCAAAGA	32728903	32728928	CATACCAATGACTAGCTGATAAGTGA	I
A19chr1-47	1A	33660138	33660158	TCTTGCATTAGACCGACCCTA	33660527	33660546	GGGAGATGCAGGAATCAACT	I
A19chr1-48	1A	33848941	33848965	TCCTAATAGATGTTTAACCAAACGA	33849396	33849415	AGCATGTTATATCGCCTCCA	I
A19chr1-49	1A	37319714	37319734	TCACACAGTCACAGACGAAGG	37320022	37320048	AGAGTTATGGTTAGAATTGGATTTAGC	IV
A19chr1-50	1A	37450555	37450575	GCGGTTGTGTCTCTCTTGTCT	37450884	37450910	AAACAAATACAGATTAAACACAATTCA	I
A19chr1-51	1A	38129471	38129497	ATATGAAATAGTATCCTTAAGGCTGTG	38129880	38129903	TGAGAAAACAAAGGAGAAGAGATT	IV

A19chr1-52	1A	39077232	39077254	TCTTTATTCTTGGGTTCCCTTTC	39077527	39077546	TACGCCCCGAGACTACTAGG	IV
A19chr1-53	1A	41320145	41320166	TTGAAATGGTTCAAATTGAGGA	41320434	41320453	TTCGACAGCCAAGTGGAGTA	IV
A19chr1-54	1A	41411640	41411661	CCTTCTTTGATGGGTATTTTCG	41411832	41411855	AACCGTCTAACTACCATTTTCCTG	IV
A19chr1-55	1A	42235996	42236022	CACAAGAATTAAAGCATTAAAAACTCA	42236200	42236218	GAGCGAAGCTGGATCCTTT	IV
A19chr1-56	1A	43312713	43312732	ACCCCAAACACATCCAAAAC	43312951	43312970	TGAATCCCTGACACAGCAAC	III
A19chr1-57	1A	44119906	44119932	TCTGTAGAGAGTGATGAAAGTTTAGAA	44120253	44120271	TGGAAATGCCACCTTTGAC	IV
A19chr1-58	1A	44548513	44548532	GTTGGTCATGGACTCATGGA	44548848	44548874	TGGGATATAATTCTACTTTCACTTTTG	III
A19chr1-59	1A	46165561	46165580	GGCTGAATCGATTTGGTTTT	46165989	46166012	AGCTAAACTCGGAAGAGAAGAAAA	IV
A19chr1-60	1A	47374936	47374957	GCCAAGGTCAATGATAAAAATG	47375196	47375216	TTTATGTGGAATTGTGGATGG	IV
A19chr1-61	1A	47776994	47777018	AATCTTAATTTTCCTTGTATGGTTG	47777340	47777364	TTTGAAGAATCTAACATTGACACAC	IV
A19chr1-62	1A	49326897	49326918	CACACCCTTTGTACAAGATCCA	49327269	49327293	TGACATGATTAGAGAGATCTTGAGG	IV
A19chr1-63	1A	49993953	49993975	AAGTATTCAAAGACCCACAAACG	49994276	49994295	TGACCTTGGTGATGGATGAG	IV

A19chr1-64	1A	52510754	52510780	TGTACTTTACTTTGTTACCTTGTAGCC	52510976	52510995	GTCAAGCTTGGGGATGTGAT	III
A19chr1-65	1A	52575693	52575712	ACCATCCTTCCTTCTCACCA	52575948	52575972	TGTGTGGAGAGGTTATGTTACTGTT	I
A19chr1-66	1A	54298199	54298223	AGAATTGAACAAGTCATACCAACTT	54298455	54298475	TGCGGAGTTGGTATTTATGTT	I
A19chr1-67	1A	56397692	56397715	TTTCAAAAGAGCATAAAATCATTG	56397905	56397927	TCTTCTGTTCGATTCAACTCTCA	IV
A19chr1-68	1A	60514316	60514336	AATGCTTTGCCTTTTGAATTG	60514584	60514603	GATTCGATGCCGTTACCACT	III
A19chr1-69	1A	63158104	63158123	ACCCGGTTGTGCTATGACTT	63158488	63158507	GCATTTCCTACACGGGATTC	IV
A19chr1-70	1A	82038787	82038810	TGGTACGCTACTATGCATTACTCA	82039030	82039052	GGGAGAAATCAACTTAAACATGG	III
A19chr1-71	1A	86116324	86116344	AAGGACCCAATTTTTGGTAAA	86116730	86116752	TCATGCGGTAAAATAATTAGCAA	I
A19chr1-72	1A	86914417	86914439	GCTTTCTAGTGCTGATTGTGTTC	86914776	86914797	TGAGGGATTTTAAGACATGAGG	IV
A19chr1-73	1A	87219796	87219816	AGTCACGGTCACAGTCACACT	87220154	87220173	TGAGGTGGGTGACGACTAGA	III
A19chr1-74	1A	87438652	87438678	CGATGTTTGATACTCTACTTGGTACTC	87438868	87438888	AGCGAAAGGAATCACAAAAGA	I
A19chr1-75	1A	90383609	90383628	CACCAAGACCTCCCACAAGT	90383897	90383922	GATGTGAGAGTCATAGAAAATTCCAG	III

A19chr1-76	1A	94773406	94773424	CTTGGGCCTTAACTTGCAG	94773800	94773823	ATGTTGTTTTGTGTTTATGCAGAG	IV
A19chr1-77	1A	94858013	94858039	TCTTGGAACACAGTAATTGACTAATTG	94858346	94858366	TTGAGAGTAAGCAAGGGTCCA	IV
A19chr1-78	1A	95596190	95596215	TTTTAAATATCAACTTTAACCGTTCC	95596654	95596674	CATCGCATTTTGGACAATAAA	IV
A19chr1-79	1A	96278480	96278500	TTTTCATGCCTCAAAACCATA	96278685	96278705	AGGTGTCGTCACGTATTTGAA	IV
A19chr1-80	1A	96702985	96703011	TCATTTCAATATAAATATAAGGCATGG	96703389	96703415	TTCCATTTTAATTGTTATATACTGCAC	IV
A19chr1-81	1A	97127097	97127116	TCAACCGGAAGAAGCTAAGA	97127442	97127464	TGAGGTGTATTTTTGTCGATTTT	I
A19chr1-82	1A	97987962	97987981	AATGCGTTCACCTCTCCACT	97988305	97988328	CTTCTTGCCATAGGTAACATGAAA	I
A19chr1-83	1A	98075170	98075192	CACGAAGATAAGTGCATTTGAGT	98075547	98075566	GAAATCGTTTGGATGGTGCT	IV
A19chr1-84	1A	98876986	98877012	CCGTTTTAACATCCCTAATATCTATAC	98877415	98877434	TATGGGGCAAAGTTTTCACA	I
A19chr1-85	1A	101123630	101123652	GAACCAAAGCTCTCATTTACTCC	101123891	101123912	CGCGCCATTTATTCTTACTATT	IV
A19chr1-86	1A	101279502	101279521	GATGACGATGATGGGATTGA	101279939	101279958	GTCTTCTTCTCCCCTGCATC	III
A19chr1-87	1A	101528646	101528666	CCCTTTAATTTCTTCCCCAGA	101529021	101529047	TTAGCTGGTCATTAAATACTGAAACAA	IV

A19chr1-88	1A	101629479	101629500	TGGATAAAGAATTGGATCTTGG	101629968	101629988	CGCATTCTCTCCAAATTTACC	I
A19chr1-89	1A	102400908	102400930	CTAGAATTCGAACATGGAGTAGC	102401272	102401291	CGGAAAAATCCGACAGTAAC	IV
A19chr1-90	1A	102429463	102429487	GCATATGAGAGAAAGGATAGAGTGG	102429862	102429880	CTCTCCACCTTTCGCACAG	IV
A19chr1-91	1A	102872524	102872549	TTCAAACATGTCTTAGTGTCTTAACG	102872860	102872884	TGGTTAGAGATGGGAGAATACATTT	I
A19chr1-92	1A	103824205	103824226	TTCTTCAACCATTTGTAAACCA	103824622	103824641	ATTGGGGACAATGAGGTCTT	I
A19chr1-93	1A	103951812	103951836	TTTTTAACTATCAATTCCGAGCTTC	103952060	103952086	GCATTAAAACGAATATCATAACATCCT	I
A19chr1-94	1A	103963195	103963219	AAGAAAATACAGGTCAAAATCCTCA	103963551	103963570	TCAAAAGGCTCAGCAACAAC	IV
A19chr1-95	1A	104456505	104456530	ATACTGCTTCTGATGACTTCTTCTAA	104456727	104456747	TCGGGCTCGTAATAAATTGAA	I
A19chr1-96	1A	106856722	106856740	GTCCAAATGCGTCCAAATG	106856933	106856954	AGGATTATACGGTGCGAATATG	I
A19chr1-97	1A	107713200	107713219	TTGACTGACACGATGTGCAA	107713693	107713711	CCCTAAACCCCACATCTGA	IV
A19chr1-98	1A	107922591	107922610	CCACAGCCTTTACTCCCTCA	107922922	107922941	GGTGTTGGCAGAGGTTAGGA	IV
A19chr1-99	1A	108362361	108362381	TCTTGAATTTCCTCTCGCAAA	108362800	108362818	GGATCCGTCTGTGAAGCAA	IV

A19chr1-100	1A	109515167	109515192	TTTATCATACGTTATTGGTTACATCG	109515423	109515442	CGGTCACTAAATCGAAACCA	IV
A19chr1-101	1A	109845712	109845736	TTTTAAAAGCGCTAATAGATTTTGG	109846089	109846110	ATTGATTACGTGCCTCTTTGTG	II
A19chr1-102	1A	110056549	110056574	TTCATTAACCATCGGTATAATTTCAA	110056873	110056893	CTTACGAGGCGGTTGTATGTC	IV
A19chr2-1.1	2A	2488663	2488689	CAATTCACAAATGAGGTATAGTTATGG	2488900	2488923	TCAATTGCTGAATACACAAGAAGA	II
A19chr2-1.2	2A	2488672	2488698	GAATTCACAAATGAGGTATAGTTATGG	2488900	2488923	TCAATTGCTGAATACACAAGAAGA	II
A19chr2-2.1	2A	91114362	91114386	GTTGTAGTGTTCAGCGTTAGTAGCA	91114603	91114623	GGCAGGGACAAATACTTCGTA	II
A19chr2-2.2	2A	91114361	91114384	GGCTTATTGTAGTGTTCAGCGTTA	91114603	91114623	GGCAGGGACAAATACTTCGTA	II
A19chr2-3	2A	964631	964653	CTTGAGTAACAGCGATAGAAAGC	964975	964992	ACGGGAACAGGACAATGG	I
A19chr2-4	2A	1593630	1593655	TTCTTTAGGGATTATGTATTTGACCA	1593856	1593878	TGACAAGGAGAACAGGTACAACA	I
A19chr2-5	2A	2489000	2489026	TTCTGTATATTCATCAATCCTAGTTCA	2489338	2489360	TCACCAAATTTTAGAAAGGCTAA	III
A19chr2-6	2A	5190932	5190951	ACCCCTACCCGATTTTGAGT	5191127	5191147	AGCTTTGATGGCTTGTTATGG	I
A19chr2-7	2A	5884317	5884340	TCACAGAATAAAATGGTCCCATAC	5884615	5884641	AGAAATATTGCTGTAGTTGTTGATTTT	II

A19chr2-8	2A	6751812	6751835	AAGGAAGACAGAGAATTGACACAA	6752212	6752238	TTTCACGAATGTTTCTTATTTTTAGAT	II
A19chr2-9	2A	6917061	6917080	TGCATACCACCGTACCTCTT	6917299	6917318	ACTGCCATGAGCTTGAAATG	I
A19chr2-10	2A	7311209	7311234	AATGATTCTTTAAATTCTCCTTAGCA	7311514	7311533	AGGAACTCGGCTTAGCATCA	IV
A19chr2-11	2A	9240389	9240414	CCATCAAATATTCTTTTACGTTTCAA	9240686	9240708	CAATGTTGGGTAACTCATCACAA	IV
A19chr2-12	2A	9525545	9525569	TTGTTAATAGAAAGTGCATTTGACG	9525912	9525934	GACCCGAATTTAAGTTGTAAACG	IV
A19chr2-13	2A	9737610	9737636	ACCCATCAACACTTATTAGTTATTACC	9738019	9738038	AGGTCACACATCACCCAATC	I
A19chr2-14	2A	9969588	9969610	GGAAAGAAACCACACACTCACTT	9969926	9969951	TTTTAGGTTTCTATGAAACACAGGTC	I
A19chr2-15	2A	11330719	11330740	AAGGACTAAGGAGCATCGATTT	11331111	11331136	AAATTGAATCTGATATGATTTGATGA	I
A19chr2-16	2A	11543255	11543280	CCCTAAACTATTGTTAGCATTTTCTT	11543599	11543620	TGCAATCCTAAAGTGATTCTCC	I
A19chr2-17	2A	12530037	12530057	CTCCTTTGGGATACTGCTTGA	12530312	12530333	TCTGAAAGTAATCTTGGGATGC	I
A19chr2-18	2A	14771983	14772008	TTGAGCTATTTAGTCGATAAGTCAAG	14772195	14772215	TTGGACGGTATTAGTTGTTGG	I
A19chr2-19	2A	15038954	15038975	CCCTCTCTGTCTCTCTCGTCTC	15039206	15039226	TGATGAGACAGAGAGCGACAA	III

A19chr2-20	2A	15502361	15502385	GAGATGAATTTGATAGAAGAACGAG	15502823	15502844	AGAATTGTCTTCTCCTGACCTG	IV
A19chr2-21	2A	15644818	15644835	TCACAATAACCCGCCACA	15645274	15645295	TGCATTTGATTATGTGTTTGGA	II
A19chr2-22	2A	18712609	18712633	TCATTGAGCCAAACATTATTTATTT	18713008	18713027	CACCCCTGCTTTGTCAAGTA	I
A19chr3-1.1	3A	12081450	12081472	TCATGAGTACTATCCCAAACACC	12081799	12081823	GGCATGCATGATTTAGTGTACTATT	II
A19chr3-1.2	3A	12081453	12081476	TGAGTATTATCCTTCACAAACACC	12081799	12081823	GGCATGCATGATTTAGTGTACTATT	IV
A19chr3-2.1	3A	128863896	128863918	TGATTTGACTAAGAAGGGTAGCA	128864344	128864367	AACATGTATTTAAGTTGGCCCTTA	II
A19chr3-2.2	3A	128863901	128863926	TGACTAAGAAGGTTAGAAGGGTAACA	128864344	128864367	AACATGTATTTAAGTTGGCCCTTA	I
A19chr3-3	3A	463865	463891	TTGAGATGGGTAGAGAACTAGGTAAAA	464103	464128	GCTAGTTTCATGTACTGTGGATATTG	I
A19chr3-4	3A	3002887	3002910	GGTTATGGTCTTCAATCTTCATGT	3003210	3003232	CACGCTTTAATCTGAGAAAATGA	II
A19chr3-5	3A	5523241	5523262	TCGGTTCACTAGCAATTTTCTG	5523604	5523627	TGGAACGGTAAAAGAACTGATCTA	I
A19chr3-6	3A	8285653	8285673	CCCCCTCTCTCTCTCTCTTCT	8286038	8286061	ATAATAACCCATTTTGCTTCAAGA	IV
A19chr3-7	3A	13284644	13284664	TCCACTCCAGTTAGGGATTTC	13284989	13285009	TGGACGAATATATCAGCGAGA	I

A19chr3-8	3A	20012402	20012423	TGCGTCTTGTTAGTGTCTCTGT	20012731	20012750	CATGTGGGGAATTCGGTAAT	I
A19chr3-9	3A	22998978	22998997	TAATTATCTCGCGCCCTTTT	22999324	22999343	CAGCTGCTGTTGCTAAAGGT	IV
A19chr3-10	3A	27672744	27672767	TTGATCCAATAAAGGAAAGTTACG	27673048	27673067	GCCGGATCAGTGTTCTTTCT	I
A19chr3-11	3A	30821184	30821203	GAACACGGAGTGGGGTAGAA	30821418	30821437	TTCATGGCTCTCCCTTTCAT	IV
A19chr3-12	3A	33706339	33706365	ATAAACTGTTGCTCTACTACATTTGAA	33706555	33706577	TGCAGTGTACAATCCAATAAAAA	IV
A19chr3-13	3A	38387674	38387693	TTAAATTGCTGAGCCAACGA	38387987	38388006	GCAATGAGAGCTCATGGAGA	I
A19chr3-14	3A	41066167	41066193	GCTAAGATAAGAGAAGACCGTTTATGA	41066406	41066432	GAAAATGTATTAGCAAAGTGTATCTCA	IV
A19chr3-15	3A	43397324	43397346	TGAGTGTGAGGGTGAGTATGAGA	43397537	43397557	GATCCATTATCCCCAATACCC	IV
A19chr3-16	3A	52117056	52117079	GAAATCAAGGTATCAAAAGGGTTA	52117367	52117386	GGCATTGAGTCAGCGATAAT	IV
A19chr3-17	3A	62365875	62365897	TGCTCATGTAGCATAGTCGGTTA	62366258	62366279	ACAAATGCGTGCATAATGTCTT	I
A19chr3-18	3A	91081539	91081559	GGTGTGACGTTTGTTTGTGAA	91081815	91081834	TGTGAGCTTCAAACCCAGAA	IV
A19chr3-19	3A	107487175	107487196	TTTGCTAATGGATGGTTTTGAA	107487579	107487601	TGTTCAACATCCATACACATCAA	IV

A19chr3-20	3A	111473977	111473996	CCTGCTTTATTTTCGGCATT	111474221	111474247	TGTTTAGATTTAGGATACAGGTCGTTT	I
A19chr3-21	3A	131443067	131443090	CAAAATCAGACACATTCATTTTCC	131443461	131443481	CTGATCTGATGTTCACCGACA	I
A19chr3-22	3A	131829057	131829075	GTTTGTGGTTGGGTTGTCG	131829318	131829342	TTGGTGTTAAAGAGAAAGTCAACC	II
A19chr4-1.1	4A	2736645	2736663	CCGATTCCAACAATGCCTA	2737019	2737044	AAAATCCACTAGAAAGTAGAGCGTTT	II
A19chr4-1.2	4A	2736651	2736671	CCAATTTCCAACAATGCCTAA	2737019	2737044	AAAATCCACTAGAAAGTAGAGCGTTT	II
A19chr4-2.1	4A	124023447	124023468	GGCAGGAACGTATGTAAGTCAA	124023752	124023778	TTACGTCATCTTATATTTTCGTATCAA	II
A19chr4-2.2	4A	124023446	124023465	TGGCAGGAACGTAACTCAAG	124023752	124023778	TTACGTCATCTTATATTTTCGTATCAA	I
A19chr4-3	4A	102509	102534	TGACGATAAAATAAAACACAACAACA	102823	102841	AGTGAAAGGGTGGGAGAGG	IV
A19chr4-4	4A	526622	526645	ATCCCCATACTCACTTAAAGGTTG	526840	526863	TGATAGAGACCGCTATTAACCAAA	IV
A19chr4-5	4A	683938	683958	TGAGTAGTGTGTGGTGCTGGT	684444	684464	GGCACTTTATGGGCTTAAACA	IV
A19chr4-6	4A	685355	685378	AAGCGAACTATAGTCTGGTGAAGT	685824	685843	CACTTTGGCATCGTTGTCTT	IV
A19chr4-7	4A	1146618	1146641	AAACAAGCTCTAGTTTCCGTTCTC	1146989	1147013	AGAATCATTATAATTGGGTTTGGTC	IV

A19chr4-8	4A	1316243	1316267	AAGAATCTTATGCTGTTATGCATCT	1316695	1316719	TGTGAATCACCTATCTTATATGCAA	I
A19chr4-9	4A	1391096	1391116	GCATCCCAAGTTGTAGCTCAG	1391331	1391350	GGCTTAGCACCAGCAATCTC	I
A19chr4-10	4A	2072925	2072944	GTGAGATGTCCGGTGATGTT	2073172	2073191	CTATTCTTCCCGCCATGACT	III
A19chr4-11	4A	2075285	2075305	AGGGGTTTGATCACAGTTTTT	2075696	2075715	GGATTTGGGGATGACAAAAG	IV
A19chr4-12	4A	2308096	2308120	GAAAGGAGAGAACTTGCATTAGAGA	2308341	2308366	CCTTCTAATATCATCAGCTGAACAAG	I
A19chr4-13	4A	3059639	3059660	TGGGCTTGATATATCCCCTAGA	3060018	3060040	TTAATCAGCCTCAAATTCATCCT	I
A19chr4-14	4A	3749998	3750020	AAATCCCTTGCACAAGTATGTTC	3750359	3750377	GCAGGGGTAGGGGGATTAT	IV
A19chr4-15	4A	4396000	4396020	AAATGAACCCATAACCACACG	4396374	4396400	AGGTCATTGACTCTCTAACACTACTCA	I
A19chr4-16	4A	5016668	5016687	ATTCTGCAAGGGAGCTATGG	5017076	5017100	CATGCATTTAAAATTTGAAGGAGTT	II
A19chr4-17	4A	5338568	5338587	AACATTTCCCTCCAGCTCAC	5338831	5338850	GAAGGCGCATCTCTCTCTCT	III
A19chr4-18	4A	5936994	5937019	GAGGGTGAGGATAATGATAGATAATG	5937216	5937242	TGTTAAACAATCATTTAAAGAGATGAA	I
A19chr4-19	4A	6031157	6031176	TTCACCATTCACCATTCACC	6031406	6031427	AAGCTCGGTTACATGTGGATAG	IV

A19chr4-20	4A	6103175	6103194	GATGATAACAGCACGCCAAA	6103517	6103538	TCTAAATTGGCGTCATAGCAAA	IV
A19chr4-21	4A	6114183	6114200	AAACTCCTCCGGGTGGTC	6114650	6114670	GCAGTGACTAAAATCCCCTTC	IV
A19chr4-22	4A	6944323	6944349	AATGACGATTACAATTTACATATCACA	6944683	6944700	TTCGTCTCGCCGTCAATA	I
A19chr4-23	4A	7404609	7404628	AATTGCAGAGGAAGCTTTGG	7404891	7404917	GCCAGTACTCAGAAGATCATCTCTATC	IV
A19chr4-24	4A	7637973	7637994	AGATGACGTGGCAATTTTAATC	7638175	7638194	CGAGAGGGAAGAAGTGTGAA	I
A19chr4-25	4A	7762872	7762893	TTGGCAAGGAAGACAATGTAAA	7763106	7763126	AAGGGAGGGTTGAAGGATAGA	IV
A19chr4-26	4A	8794552	8794576	TATATGTGTTTAATTTTTGCCCATT	8795031	8795050	GTTAGGTTGGTTTGGGTTGG	IV
A19chr4-27	4A	9311657	9311676	TCAGTGTTGTGCTGTGCTGT	9312022	9312041	GCCGCTCTCTTTTCTCAGTC	II
A19chr4-28	4A	10092427	10092450	ACTTAACTTACCAAAACACAGCAA	10092841	10092863	TGAACTCTTCCTTAGCTTGTTTG	IV
A19chr4-29	4A	10248937	10248960	AAATTAGAAAATGGAAGGAGATCG	10249367	10249390	CTCTCTTTAGTGTTCATCCTGTCC	IV
A19chr4-30	4A	11603119	11603145	TCTATGTAACAGAACTTGATCATCATT	11603447	11603466	GATAGCTCGATGGAACGACA	II
A19chr4-31	4A	12099582	12099607	ACAATGGTATTTGGATACTATCTGGA	12099874	12099893	TCCAAACGGATACACCTTCC	I

A19chr4-32	4A	14498088	14498107	TGCTTCGTCTGTTCGTCAGT	14498348	14498366	CAGTCCAAGGCAGGAACAA	I
A19chr4-33	4A	14696397	14696420	CCTTTGCATTAAGACTTCTTTTGA	14696789	14696810	AGGGTCATATTTCTCACCAACC	I
A19chr4-34	4A	15185422	15185446	GGCAATTATATCCATTACAAGTTGA	15185923	15185947	AACACACAATTAATGAAGGTTTGAC	I
A19chr4-35	4A	15578173	15578194	CACAAGCTTTCTGCATTTCATT	15578579	15578601	CTTGTATTTGCAAAGATCGGATA	IV
A19chr4-36	4A	17303324	17303350	CAAATCAGGAGAAGTTAGTGTAGTTGA	17303603	17303625	AAGCCATATACAGCCAAAATCAG	IV
A19chr4-37	4A	17420583	17420607	CAAACACAAAATGTTATGAGAATGC	17420887	17420913	GAACAGAGAAGTTAGATAGGGAGTTGA	I
A19chr4-38	4A	17826566	17826591	GAGAGATGAATTACCAATACTTCTGG	17827005	17827031	CAGTCAATGAGAAAACATATATTGAGA	I
A19chr4-39	4A	18122948	18122967	CTTGCCGTGTTAGGCATCTA	18123246	18123265	CTCTCTGTCTGGTGGCATGT	IV
A19chr4-40	4A	19422081	19422104	GTTTTGAAGTAAAAGGCTTGAGTT	19422305	19422331	CACTCCTTACTCAAATATCCATTTTAC	I
A19chr4-41	4A	21107022	21107047	AAACATAGTGTTTCACATTACCAGAA	21107357	21107375	AACCCACTGGCCACTTTTC	I
A19chr4-42	4A	22107720	22107739	TGCTCGATATTTGGTCGAGA	22108076	22108095	AGCCAAGTGGGCTAAATCAG	IV
A19chr4-43	4A	23659343	23659366	TCAGTATCCTTTTCTCTGATGGTC	23659713	23659732	GAAGAAGGGCTTGTTTGGTC	IV

A19chr4-44	4A	25752633	25752652	GTGTTCCATTTCTGGTTTGG	25752934	25752953	GTGACCTGGTTCAACAAGCA	III
A19chr4-45	4A	26855981	26856003	TTTTGCATGTCTCTCTTACTTGC	26856304	26856328	TTCTAAACATTATTCGACAAGCTGA	I
A19chr4-46	4A	27103165	27103189	GCAAATTCTTCTACCATCTTCTACC	27103396	27103416	AGTGGGTGGTACAGTCATTCG	IV
A19chr4-47	4A	28503234	28503254	GGTGTTTCCTTAGCGTACCAA	28503454	28503474	TGATGCACTGATTGAACAAGG	III
A19chr4-48	4A	30119137	30119156	AAAGCTATTACGCCATGCAA	30119372	30119395	AGGTGGTCAACTACTAAGTTTTCG	IV
A19chr4-49	4A	30457286	30457306	TCCGAAATCAAAAGAGGTTTG	30457512	30457531	ACAAGCAAACCCACCAAATC	I
A19chr4-50	4A	30483720	30483742	AAATCTAGTTCGAGCAAGCTCAT	30484058	30484079	TCCTTTGAATGTTCCTAAACCA	I
A19chr4-51	4A	30581528	30581547	ATCTCGTTGTTGCGTTGTTG	30581943	30581962	TCAATTCGGCCATAAACTCC	I
A19chr4-52	4A	31386297	31386318	GTCCTTATGGTGCTAGACTGCT	31386694	31386713	TGAGAGGAGCCAATTTTCAC	I
A19chr4-53	4A	31478254	31478273	TTCTCATCACAGGTGCCAAC	31478658	31478678	CCGTTTAACGATTATCCATGC	IV
A19chr4-54	4A	31649187	31649213	AACTTAACTCAAAATAAACCTAGATGC	31649480	31649502	AAACCACTAAGCCTAACTGTTCA	IV
A19chr4-55	4A	33609456	33609476	TTTTACCCACCTCCTCCACTT	33609748	33609771	GACAAGGAAGTCTCAGATTGTTGA	IV

A19chr4-56	4A	34314632	34314654	GCAAAGAAATATGGTAGGGTTTG	34314961	34314986	AAGGACATTTAAGGAGAGAGATGTTC	IV
A19chr4-57	4A	34610281	34610305	GCCAAGAATACAGCATAACTAGCTC	34610703	34610723	TCTTGAATTCCGTAGGACACC	IV
A19chr4-58	4A	34722370	34722396	TTTGGATTCATAAGAACTACAACAATA	34722729	34722751	TCAGTTTCTGTCAAATTTGCTT	IV
A19chr4-59	4A	34939015	34939036	GCTAGCTCGAGTCAATAACCAA	34939309	34939329	TCAATCAAACACAGGTTCCAG	IV
A19chr4-60	4A	37485298	37485321	TGATTTTGGATTGTTAGGATGAGA	37485723	37485742	TGCGAGACGTCTGGATAGAA	I
A19chr4-61	4A	37609807	37609832	TTTTGTGAAAAGATATATGGGTTGAG	37610161	37610187	CCACATGAACATCTCAATTATAGGTAG	IV
A19chr4-62	4A	38261657	38261677	CCTCACCTTTGTTCGCTATCT	38261880	38261904	TCCTCTTTACAATCTATCATCACCA	IV
A19chr4-63	4A	39774089	39774113	TCAAAATTAAACTTGTCATCATTGC	39774528	39774552	TTCATTGTTAATTTTCTTTGCATCA	IV
A19chr4-64	4A	50380772	50380798	TGCTTGAGATTTAGTTAAATATGCTTT	50381130	50381150	TCAAGTATGTCCATGCCTCCT	I
A19chr4-65	4A	50551698	50551718	CCCACCACACATGGGATATAG	50552078	50552101	AAGGGATGGTAGCTTGTAAGAGAA	IV
A19chr4-66	4A	74404716	74404738	AGAAGTCACTACCTCAAAGCAGA	74404958	74404977	GAACGTCGAAGGTCACTCAA	IV
A19chr4-67	4A	79786399	79786424	TGACGATGTGATAGGTTAAGTTAAGG	79786778	79786797	GGCAACTGGCTTCTCAGAGT	III

A19chr4-68	4A	80151603	80151629	CGATAACATAAACGTGAAGATAACAAA	80151992	80152017	TTTCTGTATAACATACCATGCATCAA	IV
A19chr4-69	4A	80904099	80904121	AGGCTTCAAAGAAGAGAGGTAAA	80904574	80904595	TTCAACTGCAAGCTTTATCACA	I
A19chr4-70	4A	83806448	83806471	TTTTCTTTGGAAGGAATACTGACA	83806701	83806722	TTCTATCCCTACCGAACCAAAA	IV
A19chr4-71	4A	91156197	91156223	TTCTTCTTTTAATATGTTGGTCATGTG	91156687	91156711	CAAGAAGTTCAGGGAAAACATAAAG	IV
A19chr4-72	4A	92468017	92468036	AGCAAACCTTGTGGAGCAAT	92468289	92468307	AGAGAAGGAGGCGCTTGAG	IV
A19chr4-73	4A	94092863	94092883	CTTCTGTTTCACCAGCAGCTT	94093317	94093334	CATGGGCCCAATCAATTT	III
A19chr4-74	4A	97151100	97151124	AATCTTCATTGAATCTTCATTTTGT	97151348	97151373	ACAGTAGCTGATAAAACAGAAAGAGA	II
A19chr4-75	4A	98751661	98751685	TCTACTCTCTTCTATCGCTTCTTTC	98751880	98751906	GAATGTGGAGATTATAAAGTTTGTGTA	I
A19chr4-76	4A	98907143	98907169	CATATATATCATCAACTTAACCCATCA	98907351	98907369	GGATGAATGGACCTTGTGG	IV
A19chr4-77	4A	99180507	99180527	GCCTGAGTTGGTTAGTGTTGG	99180997	99181017	TCCTCGTCAACCCTACATAGC	I
A19chr4-78	4A	102416518	102416543	CCTGTAACTACTTCCTTTTACCATGA	102416999	102417018	GCCAGCAGGAGGTTTAAAAT	I
A19chr4-79	4A	102597536	102597555	TCCACTACCACCAAACCAAA	102597892	102597912	AGCAGACCTTTCCAAATTTCA	I

A19chr4-80	4A	102791572	102791595	CACTTGACGTACTTCTGAATGTGA	102791843	102791861	TGCTTTGGTCGAGAGATGG	IV
A19chr4-81	4A	103005919	103005940	GCCATAAATCATAATGGTGAGG	103006234	103006260	TGGAAGGAGTATAATAAGCAGTAACAA	IV
A19chr4-82	4A	103559272	103559295	CTTGACCTGTGAGTGTAATTTTGA	103559540	103559562	TCATCTGCAATTTGTCTTCTTCT	I
A19chr4-83	4A	106567164	106567190	TTATTAAAGAGAACACAATAAGCACAA	106567527	106567546	AACGGTTGCAGCACTCTACA	IV
A19chr4-84	4A	106710534	106710553	TTTTGGTTCTGGTCCTTGCT	106710927	106710948	CCTAACAAAATTACGCCATTCA	IV
A19chr4-85	4A	108464940	108464966	AAATAAAGTTAGAGACACACATGCAGA	108465268	108465289	TCCTGAATGCATATGTTCTGGT	IV
A19chr4-86	4A	108755138	108755159	CGGCCTATTAAGAACACACAGA	108755559	108755580	GGGGTTCATATGCTTGATTACA	I
A19chr4-87	4A	109528156	109528180	GGGATTCTAGAAGTAGTGACCTCCT	109528539	109528561	CACGAAGCTTTTATAAGGGAACA	IV
A19chr4-88	4A	110468160	110468183	TTAACGGTGATACTATGTTGCATT	110468645	110468666	CGTTAGGAACCAACTAAGAAGG	IV
A19chr4-89	4A	112309344	112309365	GAATGCTAGAACCTGAAAGTGG	112309859	112309878	CCTCCATCAACAAAACATGG	IV
A19chr4-90	4A	112551644	112551663	ACAGTAGGTGGGTGTCAGGA	112552132	112552151	TCCGAGTATCCAACAAGTCC	IV
A19chr4-91	4A	116057519	116057539	TTGCATGCATTCCAATCATAA	116057754	116057777	AAAGACACTGATATTGGGGAAAAC	IV

A19chr4-92	4A	116844187	116844207	TGCTTAGGATGAGCTCTCAGG	116844598	116844618	AGCAACTCTGATTCCCTTTCC	I
A19chr4-93	4A	118309282	118309308	CACAACTTTGAGAAATATAACCATGAA	118309635	118309660	AACAAAATCAAGAAGATTGAAGTCAT	I
A19chr4-94	4A	119832397	119832421	TGCAAACAAATACAACTTCAATGTA	119832708	119832726	TATTGTTCGGGCAAAATGG	I
A19chr4-95	4A	121548044	121548068	TCCTTAGTCATAGAAACTGGTGTGT	121548240	121548263	TTCCAAACTCTCACACTCACTAAA	IV
A19chr4-96	4A	123392965	123392990	ACTTACTTGCATCTATCAAATCTCAG	123393209	123393228	CCCGCAAGTGGTACCTTAAT	IV
A19chr4-97	4A	123396934	123396953	TTTCTATCCCGCAACAGCTT	123397200	123397221	TGTTTGATTCCGAGTGTAGTCC	I
A19chr4-98	4A	123484631	123484655	TCTTGTTATAGAAACTCACGCTTCA	123485107	123485126	ACACGAAGCCCATAGGAAAC	IV
A19chr4-99	4A	123607531	123607557	AGTTTCTGATCTCTTCTATCTTCTACG	123607843	123607864	CTCATGCGAATAATGAAACAAA	I
A19chr4-100	4A	123812050	123812071	TGGCGATGAGAAAATTAAACAA	123812255	123812274	TGAAAAGCCACTATGCGATG	I
A19chr4-101	4A	124268443	124268466	TGTACAAAGGAGGTAGTATGGAAA	124268908	124268928	TCTTTGGCCATTGACTCATTA	IV
A19chr4-102	4A	125547253	125547272	TGCACATTTCCTAGCCTTTG	125547584	125547608	TTCGTTACTTGTTTTCTATTGCTTG	I
A19chr5-1.1	5A	10484862	10484885	AGACACATATGCAAGTCAGACCTA	10485133	10485157	TTAAAGTTCTGTTGCAGGCATATAA	I

A19chr5-1.2	5A	10484870	10484891	CTGCAAATCAGACTGCATACCT	10485133	10485157	TTAAAGTTCTGTTGCAGGCATATAA	I
A19chr5-2.1	5A	115540209	115540231	AATCCTATGTTCGGGTAGTGACA	115540561	115540581	TTTTCGCAAATTGACCGTATC	II
A19chr5-2.2	5A	115540218	115540239	TTCATGTTCGGGTAGTGACATC	115540561	115540581	TTTTCGCAAATTGACCGTATC	II
A19chr5-3	5A	7052824	7052846	CCCCTAATTAAACATTTGCCTTT	7053069	7053094	CATGGTTTTGATTGTCTTTAATTGAT	I
A19chr5-4	5A	10760919	10760937	TTCGTGCATGCTGTTTTGA	10761124	10761149	ATGGTAATGAAATATCCATTAGGTGA	I
A19chr5-5	5A	14012733	14012751	ACTGCCTCCTGCCATGTTA	14013107	14013129	TTCAATTCTGTTTTCATTGTCCA	I
A19chr5-6	5A	20857598	20857617	TGGCGGTGCTATTACATGAC	20857979	20857999	TTGCCTTCGATAACGTCATTT	IV
A19chr5-7	5A	26485206	26485231	CATCTTTGACTTATTTGCTAGGACTC	26485534	26485553	GAGTGGGAAGAATGGGAAAT	IV
A19chr5-8	5A	29784428	29784448	TGAAACCAAATTTCCCAAATG	29784748	29784768	CCATTTCTCCATTTGAACTGC	I
A19chr5-9	5A	33260863	33260887	GCTTTGTATGCCTTCTTCTTCTACT	33261092	33261118	ACACGGTAGTACATTAGTTCTTTTGAA	I
A19chr5-10	5A	34456438	34456464	AAGAAGCACAGAGTAAAGAGAGAAAGA	34456822	34456842	TGGAGGTACACTCCCAACACT	I
A19chr5-11	5A	37406109	37406130	TTTAATCTGTTTGCTGCTCTGG	37406426	37406452	CACTACATTAGATACTGCTTGGAATGA	I

A19chr5-12	5A	42745813	42745839	TCACCTATATTGTCTACATTGTCCTTT	42746217	42746236	CGTCAGAAGAGTGACGAAGG	I
A19chr5-13	5A	48863749	48863769	TCGCTTGCTTGTTTTCTCTTT	48863963	48863985	TCCCGATTAATAAAGATTCCAGA	III
A19chr5-14	5A	62566746	62566765	AAGCAAGTCCCAATCGGTAA	62567046	62567066	CACTATGACCGTTCCAAGGAG	IV
A19chr5-15	5A	71510536	71510558	TTTTGGTGGCTGTTTGTAACTTT	71510786	71510809	AGTTGTTAGAAAATGGATGGGAGT	IV
A19chr5-16	5A	87679126	87679152	GAAGTAGGACAAGAGAGAATATGAGAA	87679581	87679600	CCATTGATTACCACCAACCA	I
A19chr5-17	5A	91019981	91020001	AAATTGCCATACCCTGAAATG	91020316	91020338	TTCGCTTTTAGTGAATTTGTTTG	III
A19chr5-18	5A	96884517	96884541	GAATAAGATACGGATCGGATAAGAA	96885020	96885039	AGGGAAAATTGTCGGTTGAG	II
A19chr5-19	5A	100244425	100244444	GGATCTCAATCATGCGTTCA	100244665	100244686	GGACTGCTTATCTGAGTTGCAT	IV
A19chr5-20	5A	101318094	101318120	CACTTCTAGCCTTTTAGGTATACTTTG	101318406	101318428	CCTGAGTATCAGTCATTTGCTTT	I
A19chr5-21	5A	103861052	103861071	TCCACAACTACACGCACACA	103861448	103861467	AGTCACAGATTTGGCAACGA	IV
A19chr5-22	5A	104601068	104601087	TGCAAATGCAATGATCAAAA	104601521	104601543	TCCAATTTGAATTAAATCGATGA	IV
A19chr6-1.1	6A	837187	837207	TCATGATTGCAGCTTGAGTTC	837452	837474	TTGTCCATTCTCAGTCCATACAA	II

A19chr6-1.2	6A	837202	837221	GCAGGTGTCAGCTTGAGTTC	837452	837474	TTGTCCATTCTCAGTCCATACAA	II
A19chr6-2.1	6A	116369745	116369766	CCTAACCTAACCCATCAAAACG	116369955	116369977	TGCTACTCTCAGTGTTTGGTTTG	II
A19chr6-2.2	6A	116369742	116369761	AGACCTAACCCATCCAAACG	116369955	116369977	TGCTACTCTCAGTGTTTGGTTTG	II
A19chr6-3	6A	1539692	1539712	CACACACACACAGACGATTGA	1539991	1540010	AGCTGTTGATCCCTTCCAGA	III
A19chr6-4	6A	2954594	2954614	CATAGTGATGTGTGGCGGTAA	2954911	2954929	GAATCCGAGAGGGTTGGAG	IV
A19chr6-5	6A	5607672	5607691	TGGGGAGAATTTCATCAGGT	5607992	5608014	TCGCTTTTATTCAAATTGCCTAT	IV
A19chr6-6	6A	6233606	6233631	CCAAACTCCAAACAAGTATAGAGAGA	6234100	6234123	TCAGAATCACGTCAGTCACTAGAA	IV
A19chr6-7	6A	7844559	7844585	TGTATATATTTGTTAGCCGTGATTAAA	7844771	7844790	CGTCCTTTGCCTCAAGATTT	IV
A19chr6-8	6A	8349895	8349916	GATGATGTTGGATCCAGAGAAA	8350226	8350245	AATTCCTGAAAACGGGAGAA	I
A19chr6-9	6A	10108510	10108527	TGCGTGCCAATCGAATAA	10108913	10108932	CAAAATGAGGGGAGATAGGG	II
A19chr6-10	6A	12293799	12293820	GCCTTAACCAAACAAGAGTTCC	12294216	12294234	AGAAGGCCATCACAACGAG	IV
A19chr6-11	6A	14250287	14250306	CGAGTAGGCGGGTACTCTCT	14250580	14250605	GATAATTTAGGACCATCGTTAAGAGA	I

A19chr6-12	6A	17092273	17092296	AAACACATGTCAAGCACACTATGA	17092626	17092649	AGTGTGTGTCAACTTCATGTGAGA	I
A19chr6-13	6A	19123298	19123318	TGAATGTTGTTCGGTTCTGTG	19123549	19123569	CCGTTTTTATCTGTTCGCATT	III
A19chr6-14	6A	32780584	32780604	TTTATCGCCAGTGCCTTAGAA	32780943	32780966	TTGAAGAGATCTTGGAATCAACAG	I
A19chr6-15	6A	71676173	71676196	CGAGACAAATTAGCCATACTTTCA	71676514	71676539	CAACTACCATAGGTTGAATGTTTAGG	IV
A19chr6-16	6A	81759343	81759368	TCAATATATAAATTTGGACTGGGCTA	81759673	81759692	ATGGAGGGATGGAAAAGTGA	IV
A19chr6-17	6A	83628195	83628214	GGTGGCGATCGATCTATGTT	83628448	83628469	TCAGTGTCTTCAGGAAATGAGC	IV
A19chr6-18	6A	94714210	94714229	CCAGAAAAGGGTGGATTCTT	94714661	94714682	CCTCCAGACCTACCAATAGTGA	IV
A19chr6-19	6A	98926464	98926481	GGGCGGCCAGAGTATGTA	98926754	98926773	GATCCTGGAAAGCCTTCTGA	IV
A19chr6-20	6A	102819030	102819055	ATTTGTTCCTTAGCCTTAGCTTATAC	102819235	102819258	TGTCACTTGGAAGAGAACTAAACA	I
A19chr6-21	6A	103910277	103910297	CAAAATTTATTGAAGCGGTGA	103910628	103910647	AGCGAAGCAGAAGAAGATGG	IV
A19chr6-22	6A	106123935	106123954	CGTTTTCATGACCCCTTCAT	106124162	106124181	ATGCCGCATTGCTTCTATTT	I
A19chr7-1.1	7A	4382866	4382890	CACCAATGACCTAAGATTACAAGTG	4383221	4383241	CACACAAACACAAACAGCACA	IV

A19chr7-1.2	7A	4382866	4382891	CACCTAGCAATGACCTAAGATTACAA	4383221	4383241	CACACAAACACAAACAGCACA	IV
A19chr7-2.1	7A	60040914	60040939	TGAGACTAATTTCATACGATACAACA	60041323	60041342	CGGATTTTACCAAACAAGCA	II
A19chr7-2.2	7A	60040930	60040952	CGATACTCCTCATACTTGCAACA	60041323	60041342	CGGATTTTACCAAACAAGCA	I
A19chr7-3	7A	2283462	2283486	CCCTAACGCTACATAGTACATACCC	2283732	2283751	CATGGCGTCTGATTCTGAAC	I
A19chr7-4	7A	6321900	6321919	TAAGGATGGGAACTGGGAAG	6322101	6322121	CTGTGACGTCGGATAAACAAA	I
A19chr7-5	7A	8105015	8105035	TTGTGTGGAGATAGGGTTGCT	8105307	8105329	CGATCTTCCTAAACTAGCAACCA	I
A19chr7-6	7A	10223075	10223093	CTACCGCTGCTAGCAATCC	10223465	10223485	TGAGCCTCCTATCTTCCTTCC	IV
A19chr7-7	7A	11879937	11879956	AACACACGTTAGTGGCATGG	11880187	11880208	TCGACCATCTCAACATAAGGAG	I
A19chr7-8	7A	13122162	13122184	CAACTATTGTCATTCTTCGGAAA	13122652	13122671	GCTCCCAGCATACTCACAAA	I
A19chr7-9	7A	19438618	19438643	TTATTTTTATGCATATTTAGCGATTG	19438830	19438851	CCAACTCAATTCAAAACTCCAT	I
A19chr7-10	7A	22548673	22548693	TTAACAACCTAAGCCTGTGGA	22549137	22549156	AAGATGGGCTAAAACCGAGT	I
A19chr7-11	7A	25959634	25959654	TGAAGTTGAGATTTGGGGAAA	25959950	25959972	CATTATCCAAATCTCCTTCCAAA	I

A19chr7-12	7A	31241546	31241566	GACCTATACCCATCCCGAAAG	31241975	31241995	CAGAATGCACCTCAAATTCCT	IV
A19chr7-13	7A	42624833	42624855	TCATGCAATTGTTGAGCTAGATA	42625068	42625089	GAAGTCCGAGAGATTTAATCCA	IV
A19chr7-14	7A	48644708	48644728	TCCTCCAAGAATGGTTCTTCA	48645019	48645041	CTCCAACATTTTGAAAGAAGGAA	IV
A19chr7-15	7A	53880917	53880937	CCAAAGCCTATTGTGGTTGAA	53881423	53881446	GCAGTGAAAATACAAGAACGAAGA	IV
A19chr7-16	7A	57921048	57921066	CTGTCTAAGCACCGGGACA	57921503	57921524	TGAGGAGGAAAATACACACGAG	I
A19chr7-17	7A	63514917	63514940	TGAATAGGGAAGTTTGTTTCACTT	63515239	63515262	TCCAATTAGTCAACCCTGTAAAAT	IV
A19chr7-18	7A	66745347	66745368	AAAGAAAAGAGGGCAAGAGTGA	66745565	66745584	TTGATGGTGAGCATCGAAGT	I
A19chr7-19	7A	69881356	69881379	TTTCTCATATTAGCTGTAGCCGTA	69881815	69881834	TAACGCGACATCTAGGCAAG	I
A19chr7-20	7A	73025380	73025398	GTATGTGCGGTGCAGGACT	73025571	73025595	TGCTATGAGGTCAATAGATTTTGTG	I
A19chr7-21	7A	79448409	79448433	CAACCTTAGTTAGCATTCTTTCTGG	79448849	79448871	AACTTCTGCTAACAAAGGGACAA	IV
A19chr7-22	7A	80362299	80362318	ATGTGTTCCGATTCCGTCTC	80362571	80362595	CAAAGTCTAACTAGAGCAACAAGCA	I
A19chr8-1.1	8A	135385	135404	CCGGAAATCAAACGTTACAA	135744	135765	CACACATTCAGTCACGTTCTCA	II

A19chr8-1.2	8A	135387	135413	GGAAATCAAACGTTAAATGTTACATAC	135744	135765	CACACATTCAGTCACGTTCTCA	II
A19chr8-2.1	8A	39128973	39128995	TGCTTGTGATCAAAGGACTAATG	39129333	39129356	TTCAATTAAGCTCACGAATTAAGA	IV
A19chr8-2.2	8A	39128977	39128998	TGTGATCAAAGGAGTGAAAGGA	39129333	39129356	TTCAATTAAGCTCACGAATTAAGA	I
A19chr8-3	8A	1972029	1972048	CGATGATACTCGGTGAGCAA	1972528	1972550	TCCCAACCAACCATTATAAAGAA	IV
A19chr8-4	8A	4529249	4529272	TATAGAAACAACAGCCCATAGAGG	4529476	4529500	TGGTTTAAACATAGTTTCAAGGCTA	IV
A19chr8-5	8A	9378633	9378656	TCACTCTATCCCTTTAATTTGGTG	9379042	9379061	CATCACCACCAAATTTCACC	IV
A19chr8-6	8A	10561954	10561980	TTTTGATTTAGGCTTTTATTACTAGGC	10562402	10562425	TTCAACTATTCATCTCTCACACGA	I
A19chr8-7	8A	12844176	12844198	AGATAATCGAATACTCGCTACCA	12844559	12844578	AAAGGTTGAAACGGCAAAAG	I
A19chr8-8	8A	15980404	15980426	TTGCTTCTCAGAACTTCAGTCTT	15980580	15980603	TCTTTCTTTTCACATAACTCCTCA	I
A19chr8-9	8A	17337499	17337524	CAGAAAGTTAACCAATGGATATGAAA	17337698	17337722	TGAGAGAAGAATTGCACTTTAGTTG	I
A19chr8-10	8A	17658693	17658716	GAAACCAAATATAACTGGCAGAGA	17659066	17659085	GTGTAAATCTGGACGCTTCG	I
A19chr8-11	8A	18581426	18581448	TTAAATTCAGAAAGCCATTGACA	18581673	18581691	AATGACAATGGGTGCTGGA	IV

A19chr8-12	8A	27425831	27425855	TTAAAAAGCCTTTGTTTACTTCATT	27426146	27426170	AAGTAGGAGTGTAAAGGCTTGATTA	IV
A19chr8-13	8A	32668762	32668786	TTGTATGTAGCTGATGGAAAGATTG	32669008	32669026	GACGCTGCCCTAAAAGGAG	IV
A19chr8-14	8A	34699595	34699619	AAAATCGTGTTACTTGATTAATTGG	34700067	34700086	TGATCACCTGAAGGAGCTTG	IV
A19chr8-15	8A	36510796	36510818	CATTTTGAGGTCTTAACTGATGC	36511147	36511168	TGCAAGTGAGATAAAGGTAGCA	I
A19chr8-16	8A	39025662	39025683	CAATTGGTGAGCTTTAGTCCTC	39025917	39025935	CAAGCACCAAAGGACATCA	I
A19chr8-17	8A	39944518	39944544	TGACTATGTGAGATAAAATCTGTTACG	39944805	39944826	CAATTCTCCAATCTTACCCTGA	I
A19chr8-18	8A	41917364	41917387	TGACGTGTCTGTCTGTATTATTGC	41917542	41917565	AACCCAGCAACTCTTACTTATGTG	IV
A19chr8-19	8A	43615630	43615649	AGGACGCGACCAAGTTCTTA	43615927	43615953	TCCTAGTCATATGATAAATAACGGAAC	IV
A19chr8-20	8A	45382693	45382717	TGCCTTCAATCAAATATAAGTGTCA	45383018	45383040	TCCACTTTCTAGGGTTTCTCAAA	IV
A19chr8-21	8A	47823013	47823035	TTCCTTGATTCTGTCTTGTTTCA	47823381	47823402	GCTCTTAATCATGCCAGCTAGA	IV
A19chr8-22	8A	50054220	50054246	CCATATGGTCTCTATTACTTCAGATTC	50054453	50054472	AACCTGCATCCCAGAATCTC	III
A19chr9-1.1	9A	14234862	14234883	AAGCAATCCATCCAGAGAGAAG	14235080	14235104	AAGAAGACAATATCAACAAATTCCA	II

A19chr9-1.2	9A	14234868	14234888	TCCATCCAGACTCCAGAGAGA	14235080	14235104	AAGAAGACAATATCAACAAATTCCA	I
A19chr9-2.1	9A	115579281	115579302	TTTTCGATCCCTAACTACATGG	115579731	115579750	TTTGAAGGTCCCTTTCTTGC	II
A19chr9-2.2	9A	115579298	115579319	TTTGTCCATGGATTTCACACTC	115579731	115579750	TTTGAAGGTCCCTTTCTTGC	II
A19chr9-3	9A	1688127	1688149	TTGGATGCATTGAAAATCATTAC	1688351	1688371	TTGGATTGTAATTGGAGCACA	IV
A19chr9-4	9A	2959620	2959639	ATTCCACAAGCCAGTGCTAC	2959849	2959869	TTGTTGTTTTGGGATTCTTGT	IV
A19chr9-5	9A	5821815	5821835	TGTATTCAACCAGGGATGTGA	5822230	5822249	TTCCTGCCCTTCCTCTATGA	IV
A19chr9-6	9A	7782308	7782329	TTTTTGGGATAAATGAATAGGG	7782588	7782607	GTTCACTGGAAGGCAACATT	I
A19chr9-7	9A	13081543	13081562	CACGCCAACACTCATTGTCT	13081738	13081758	TGGATCGTCACTTCACACAAC	IV
A19chr9-8	9A	16429065	16429091	GTGAATTCCTTATCTCCTTAATTATCA	16429305	16429325	CAAATTCACTCCTCATCAACC	I
A19chr9-9	9A	21034293	21034315	GGACACCTAGATACCCTTTGAGA	21034622	21034645	GAACGGATATGATTTCATGTTTTC	IV
A19chr9-10	9A	29730088	29730111	CAAATCAGCCCCATATATGTATTC	29730337	29730356	GGCATGCATGACTTGCTATT	IV
A19chr9-11	9A	36767190	36767208	GGCGATGAGTTCCTTCCTC	36767377	36767400	GAGGTTTAGAATTAAGTGCCCAAA	IV

A19chr9-12	9A	49564393	49564413	TTCGTTTCCACTGACAAACA	49564649	49564666	CTAAAATGCCGTGCGTTG	IV
A19chr9-13	9A	85316975	85316998	TGAACTTATATGTGAACTGGCTTG	85317212	85317231	GGGCTGTGTCTCTAGGGTTC	II
A19chr9-14	9A	88092010	88092028	CCCTCCCTTCATGTGGAAT	88092484	88092506	CAATTAAGACTGTGAGAGCGACA	IV
A19chr9-15	9A	96582001	96582020	GCCCCATTCCATAGTCCATA	96582390	96582410	ACGCGGTTTACGAATAAAGAC	I
A19chr9-16	9A	98472657	98472680	CTTGCTTATCAAATCTCGTACTCA	98473025	98473044	CATGTGTCAAGCGGGTAGAA	I
A19chr9-17	9A	106253760	106253781	CCGGTGATGTAAGAAATGAAAA	106254027	106254047	CCCTTACAACCACCAACAACT	I
A19chr9-18	9A	110454105	110454131	TTGAAGATGAAAGAATTTATAAGAACA	110454328	110454347	TGAAGTCACTGGTACGATGG	IV
A19chr9-19	9A	112246758	112246778	CCGAATCTGATTTCTCAAACG	112247252	112247272	TGTCGAGAAATCTGTGGAGGT	IV
A19chr9-20	9A	114823940	114823964	CCTTGGACCACTGTGTATGTAGTAT	114824136	114824157	TGCGTTATTTATTTTGCCAGTT	I
A19chr9-21	9A	115596866	115596885	TACGTTATGCCAGCTCATCC	115597220	115597242	AAGGCCAATAAACTTCAAAGTCA	II
A19chr9-22	9A	120408203	120408225	TCATACCAGCGAATTGAAGTACA	120408431	120408455	AAATGTGATATGTTGTGTACGCATT	I
A19chr10-1.1	10A	955960	955984	TTGTTTACATCAACCTCATAATCGT	956210	956234	GCTTTAAATGATAAGAGGTGGTTTC	II

A19chr10-1.2	10A	955963	955987	TTCTCATACATCAACCTCATAATCG	956210	956234	GCTTTAAATGATAAGAGGTGGTTTC	IV
A19chr10-2.1	10A	116925961	116925983	TCTCCATTTAATTCAGAACCTCA	116926349	116926370	GCCATAAATTTGATGTGTTCCT	II
A19chr10-2.2	10A	116925982	116926004	TTCAGAACCTCATATTTGGTAGC	116926349	116926370	GCCATAAATTTGATGTGTTCCT	II
A19chr10-3	10A	636247	636268	TCAGCGAACTATCCGTATTTCA	636454	636479	GCAATAGTTGGTCAATAGTCATGTAT	I
A19chr10-4	10A	3936203	3936228	TCCTGTTCAGTGTTCATAATAGTCAT	3936512	3936533	AGTGGAGGGGTTAACTTCAGAT	I
A19chr10-5	10A	4393406	4393426	TGCAAGCCCATACTCAGTAGA	4393618	4393637	GTTTGGCTAACCGGAAATGT	III
A19chr10-6	10A	7417106	7417130	CCTACATTTTAATGACGATGATTTG	7417558	7417583	TGAATTACTGTACTTGGATTTCTCCT	IV
A19chr10-7	10A	11174112	11174133	GGCAAGTCTCAACTCTCACCTT	11174544	11174567	AATTGACTGATATGACATGCAACA	I
A19chr10-8	10A	14981399	14981424	AAATTAAACAATCTTCTTAGCATTCC	14981815	14981841	CGTTATTTCAATCTAAATCAATAGACA	IV
A19chr10-9	10A	20064378	20064398	TTGTTGGATGATACGGATTGA	20064647	20064666	ATTCAATCCTCAACCCTCCA	I
A19chr10-10	10A	24246291	24246311	TTTTCTCTTGAGCTTCCTTCC	24246604	24246623	AAGACGGATCTAGCATGAGC	I
A19chr10-11	10A	28527226	28527252	GAATTACTCAATGGAATTACTCAATGT	28527440	28527463	CTCTACCTATACCGCCAGTATGAA	I

A19chr10-12	10A	32074636	32074658	AATCCTCTAACTTGCCACCTAAG	32075008	32075027	CAAGGCTAGAACGCAGAGAA	IV
A19chr10-13	10A	64035448	64035471	GAGCTCAAGATGTGTAGTGTATGC	64035697	64035721	TGACTCGATTAACTTCCTATTTTCA	II
A19chr10-14	10A	76812835	76812854	ATAGCATGCCAGAACCTGCT	76813305	76813330	TTACTAATCCAGTTCATCTGGTATGC	IV
A19chr10-15	10A	79988270	79988293	TGAAAAGAACACCACAATAATGAC	79988487	79988510	TTGTAAACTTCTTAGCTTGCTGTG	IV
A19chr10-16	10A	89583016	89583039	TGGTATATTTTGGAAGAGATGGAA	89583308	89583328	TTGTTAAGCCTGCAAAAATCA	I
A19chr10-17	10A	92371207	92371229	TGAATGAATACCCTGACAATAGG	92371574	92371592	AGATGATTGACCGGCCTCT	IV
A19chr10-18	10A	95042496	95042518	GACCTTTTGTTACCTCATCATGC	95042702	95042721	AGACCTTCGACCCCAGATTT	IV
A19chr10-19	10A	98542052	98542073	TCTATTTTCGTGTGTGGTCCTC	98542475	98542496	CGTTATACTCCAGGTTGCCTAA	I
A19chr10-20	10A	105216905	105216927	CCCAATTTCCGTATATGATTTGA	105217154	105217176	GGCAAGGATACCTTAAAGTGGTT	I
A19chr10-21	10A	107742929	107742948	TCAAGCTACCACCTCAACCA	107743395	107743417	CAGGAACATTATTGTCACTGCAT	I
A19chr10-22	10A	110511421	110511445	CATCATCATCATCATCATCATTAAA	110511697	110511716	CGCGTACGAGAAAGGTTACA	I
A33chr1-1	1A	475789	475809	CCAATCCAATGCAAGTTTGTT	475997	476016	TGGGATGAATAAACGTGCAA	IV

A33chr1-2	1A	5053409	5053434	CACAACTAACCTTCTTTACTTCCTCA	5053890	5053911	CAGATTTCAGCCCAATGTTAGA	IV
A33chr1-3	1A	12300124	12300145	AATAGAACCAAGAAAGGCGGTA	12300593	12300612	TTCATAGGGTTGGAGGAGGA	IV
A33chr1-4	1A	18305025	18305045	TCTCAGGGTTGTCTCCTTTCA	18305225	18305246	CCAAAACCTAGAAAGAGGTTGG	IV
A33chr1-5	1A	31539444	31539470	CACAAAATACTAAAGTGTTCAAAACAA	31539871	31539890	TAAGGTCGGGATCTTGAAGG	IV
A33chr1-6	1A	37319714	37319734	TCACACAGTCACAGACGAAGG	37320022	37320048	AGAGTTATGGTTAGAATTGGATTTAGC	I
A33chr1-7	1A	49326897	49326918	CACACCCTTTGTACAAGATCCA	49327269	49327293	TGACATGATTAGAGAGATCTTGAGG	IV
A33chr1-8	1A	58599193	58599211	TGGTGATTTGGAGGTTTGC	58599616	58599636	TGAAAAGCAAAGGCTGGATAG	IV
A33chr1-9	1A	94773399	94773417	CTTGGGCCTTAACTTGCAG	94773800	94773823	ATGTTGTTTTGTGTTTATGCAGAG	IV
A33chr1-10	1A	103934590	103934614	AACACGTATGGTCTACACAATTTGA	103934947	103934973	TCCATTACTTTATGACAATATGAGTGC	I
A33chr1-11	1A	572344	572365	TTTATGAGCTCCTTTCCCATTC	572721	572743	TGGGGTAGATCATTTTGGTAGAG	III
A33chr1-12	1A	1073181	1073200	ACTGCATGCCAGTGTTATGG	1073445	1073468	TGAGATTTACAAAACATGCTACCC	IV
A33chr1-13	1A	14820848	14820873	AAGAGTTGTTCAGCTCTACTTCCTTT	14821065	14821089	CGACAGACAAGTAGTGGTAATTGAA	I

A33chr1-14	1A	36256190	36256214	TTTTCCTTATACTTTTCAGTCACCA	36256497	36256520	TTCAAATTCTCAGCTTATTCAAGG	II
A33chr1-15	1A	86116324	86116344	AAGGACCCAATTTTTGGTAAA	86116731	86116752	TCATGCGGTAAAATAATTAGCA	I
A33chr2-1	2A	964631	964653	CTTGAGTAACAGCGATAGAAAGC	964975	964992	ACGGGAACAGGACAATGG	I
A33chr2-2	2A	5884310	5884334	GCTAGCTTCACAGAATAAAATGGTC	5884615	5884641	AGAAATATTGCTGTAGTTGTTGATTTT	IV
A33chr2-3	2A	9240389	9240414	CCATCAAATATTCTTTTACGTTTCAA	9240686	9240708	CAATGTTGGGTAACTCATCACAA	IV
A33chr2-4	2A	14787468	14787487	AATCCGACCTCTTCTGTGGA	14787779	14787805	GAATACGACACACAGGATATACTTACA	I
A33chr2-5	2A	23590237	23590260	CACTCTGCTTACTCTGCTTACTGT	23590630	23590650	TCCCAAATCTTCATTTGTTCA	IV
A33chr2-6	2A	46592616	46592642	CTTCTCTTAATGATTCCACATACACTA	46592840	46592863	GCAGAAGGAGAAGAAAGATCTACA	I
A33chr2-7	2A	61023027	61023053	TGATTGAGACAGTAAACTTAGCTGAAA	61023310	61023328	GCGTTGTCCGCTAGCATAA	IV
A33chr2-8	2A	71645497	71645520	TCTCATAAATCCTGCTTTACTCCA	71645738	71645757	TTCTCCACTGCCAGCAATAA	IV
A33chr2-9	2A	84344162	84344183	TTAATGGTAGGAATGCAAATGG	84344645	84344664	ACGTATCTGGGGGACACTTC	I
A33chr2-10	2A	91591251	91591270	TCATGTGCTTCTCCACTGCT	91591611	91591630	TTAGACGGGTTTCTGCAGGT	I

A33chr3-1	3A	340022	340048	GAGACTAGAGAGAAGACTTAAATGCTG	340477	340496	ATCGTCCCAGCAGCAATTAT	II
A33chr3-2	3A	7613287	7613311	TCAATTATGTGATATTGTGTTGCTG	7613559	7613581	GCACATTATTATGATTGGGGAAC	I
A33chr3-3	3A	19267790	19267809	CTATCTCACGCGCTCACAAT	19268166	19268192	CAAGACTAGCTAATAAGAAATTGGTTT	I
A33chr3-4	3A	32290623	32290645	GGCCTCATAAAACTTTGAGACAA	32290831	32290857	TCTCTCCAGTAATATCTTTATGACGAA	I
A33chr3-5	3A	43397324	43397346	TGAGTGTGAGGGTGAGTATGAGA	43397537	43397557	GATCCATTATCCCCAATACCC	III
A33chr3-6	3A	57094659	57094685	TGTGAAGTACTAAAGTGTGAACTGTGA	57094860	57094879	TCCCCTTGAGTACACCCCTA	I
A33chr3-7	3A	84263858	84263881	GTGATAGTGATGGCTACGATTTAG	84264272	84264291	TCCCCTAACCTGTCAACTCC	I
A33chr3-8	3A	106944964	106944988	CATCTGTACCATAGAATCAATTTGC	106945352	106945372	AAGGGATGAATGTGTGCCTAA	IV
A33chr3-9	3A	115810042	115810068	ACTTTTGATGTAGCTATACATCTGTCA	115810495	115810520	AATACAAATTGTGAATATTGATTCGT	I
A33chr3-10	3A	128693305	128693330	TAGAAGAACCGCAAATTAAATAAATA	128693514	128693533	TGATGCAAGCAAAAGTAGGC	IV
A33chr4-1	4A	102509	102534	TGACGATAAAATAAAACACAACAACA	102823	102841	AGTGAAAGGGTGGGAGAGG	III
A33chr4-2	4A	2072925	2072944	GTGAGATGTCCGGTGATGTT	2073172	2073191	CTATTCTTCCCGCCATGACT	III

A33chr4-3	4A	7915215	7915239	TCAGAGTCCTCATTCACCATATAGA	7915437	7915455	TTGCGCAGATCGAATTCTT	IV
A33chr4-4	4A	17303322	17303343	AACAGATCAGGGGAAGTTAGTG	17303604	17303625	AAGCCATATACAGCCAAAATCA	IV
A33chr4-5	4A	30197870	30197889	ACGATGGTTTACGTCACCTG	30198337	30198357	GAAGGGAATTTGGAGGACTTT	IV
A33chr4-6	4A	50380772	50380798	TGCTTGAGATTTAGTTAAATATGCTTT	50381130	50381149	CAGGTATGTCCATGCCTCCT	I
A33chr4-7	4A	77443986	77444011	AAGCATTGGTAATTTGTCAATATCAG	77444439	77444461	TTTCCCTGAAAAAGATGAGTTTT	I
A33chr4-8	4A	92468017	92468036	AGCAAACCTTGTGGAGCAAT	92468289	92468307	AGAGAAGGAGGCGCTTGAG	I
A33chr4-9	4A	102791579	102791604	CGTACTTCTGAATGTGAATATCATTG	102791827	102791846	ATGGCGCACTAAGACTCACA	IV
A33chr4-10	4A	113730372	113730397	GCATATTGCATATTGGTATTATCAGG	113730858	113730877	TTAAGCCCCTTCTCTGCATC	II
A33chr5-1	5A	6399139	6399159	CAAGGCAACAAACACATTCTT	6399618	6399639	GAGTGTGAAAGCGATAATGACA	IV
A33chr5-2	5A	9358778	9358797	CCATCAGCACCTGAGTTACC	9358994	9359013	TCTAAAATAGCGTGCCCTCA	I
A33chr5-3	5A	20857601	20857623	CGGTGCTATTACATGACCTTTAC	20857979	20857998	TGCCTTCGATAACGTCATTT	I
A33chr5-4	5A	28460846	28460866	ACAGTGCTTCAAAGAGGAGGA	28461089	28461111	GAGTTGTTAGCGTTATCATCGAA	IV

A33chr5-5	5A	33638752	33638778	ACCTCAAAGTAATAATAACCTTCATTG	33639066	33639089	TGCAGGAACTAGTGTGTAAAAGTG	I
A33chr5-6	5A	42745813	42745839	TCACCTATATTGTCTACATTGTCCTTT	42746217	42746236	CGTCAGAAGAGTGACGAAGG	IV
A33chr5-7	5A	71775582	71775602	TGCTAAGCTGGCCACTGTATT	71775784	71775803	AGTGCGTTGTGAAGGAGGAT	I
A33chr5-8	5A	94690611	94690636	TTACGAATTTAAACTAGTCCTTGTGA	94691062	94691083	CAACCTTTAAGATCCACACACA	IV
A33chr5-9	5A	100392839	100392862	TTGGGTCATTATTTTAGCTTCCTT	100393284	100393306	TCGGTACTAATGACACATTTTCG	IV
A33chr5-10	5A	107756638	107756662	GGAACAAAATGGACTTAACTCATAA	107757027	107757046	GAGACCCAATTGGCTTGATT	I
A33chr6-1	6A	356690	356716	GACTAGTGATAAAGGGAATGACAAATA	356912	356931	GGGACCATTGACCTTCAAAA	IV
A33chr6-2	6A	4874402	4874424	TGATCCTGATCCTGATTCTGATT	4874771	4874796	TGTTTTGTTATGGTTCTTGTCTTATG	IV
A33chr6-3	6A	9988741	9988760	GGCTGGATCAGCGAACTTTA	9989185	9989210	TGTTTTATGTGTGAAAATGATACCTG	I
A33chr6-4	6A	19098438	19098461	GATCTAAGACCCACAAATAATGCT	19098870	19098892	TTAGTAACTGTTGGTCCATCTCC	I
A33chr6-5	6A	26484890	26484911	TGAAGCTAATCAAACCTCCTCA	26485269	26485290	GTGGCAAATGTTTGATGATTCT	IV
A33chr6-6	6A	47795296	47795316	GGATGGGACGATGGAGTTAAT	47795687	47795707	AGAAGCAAAGCAAGCCAATTA	I

A33chr6-7	6A	71676173	71676196	CGAGACAAATTAGCCATACTTTCA	71676514	71676539	CAACTACCATAGGTTGAATGTTTAGG	IV
A33chr6-8	6A	81941274	81941293	GCCAGCCATTCTTGATGATA	81941490	81941509	TATGAGCGTCGTCAAAATCG	IV
A33chr6-9	6A	91126595	91126617	TCCTCCTTAACCTTTTCACACAG	91126920	91126939	AGCCTCAACCCTTTACAACG	I
A33chr6-10	6A	101751803	101751822	GCACGTGTGGTGATTTGTGT	101752038	101752062	AAAGGTTTGTTCGATTAGATCTTCA	IV
A33chr7-1	7A	6561600	6561625	AACAATAGCTTCTTTTATGAAATTGA	6561905	6561923	CTGAAAGGCGGTGCATTAT	IV
A33chr7-2	7A	9023783	9023802	GCTGGAGGATGGAGGATAGA	9024090	9024114	ACTACTCAGGATAGAATCGGACAAA	I
A33chr7-3	7A	13280411	13280429	CACCCTCACCAGTCACCAG	13280808	13280828	TGGAAAGCTAAGGAAGCATCA	I
A33chr7-4	7A	21073738	21073761	TGTCATGGTAGAACTTATGACACA	21074035	21074057	ACATTTATGTGGTTGCATTAGGT	I
A33chr7-5	7A	31241539	31241560	AAAACAGGACCTATACCCATCC	31241976	31241995	CAGAATGCACCTCAAATTCC	IV
A33chr7-6	7A	52079960	52079982	AAGTATTTCACACTCCCGTTTTT	52080398	52080418	ATCACAAGTTCCGAAACATCA	IV
A33chr7-7	7A	56840129	56840154	TTTGTTGATCAAATATTTTGGTTTTC	56840440	56840459	CCATTTCTAGCCTGCTCCAC	IV
A33chr7-8	7A	63514912	63514937	CACTATGAATAGGGAAGTTTGTTTCA	63515155	63515174	ACGATGATTAGGCGGTGTCT	I

A33chr7-9	7A	69881356	69881379	TTTCTCATATTAGCTGTAGCCGTA	69881815	69881834	TAACGCGACATCTAGGCAAG	I
A33chr7-10	7A	78822017	78822042	TTGTACACATAGCATAACTCAAATCA	78822325	78822344	GGGAAGGAAATTGCAGAGTC	I
A33chr8-1	8A	135409	135434	CATACTTACATCACATAGACGATTCC	135744	135765	CACACATTCAGTCACGTTCTCA	I
A33chr8-2	8A	4529245	4529268	AACCTATAGAAACAACAGCCCATA	4529476	4529500	TGGTTTAAACATAGTTTCAAGGCTA	IV
A33chr8-3	8A	8056817	8056837	GAAAACAGGCTTGGTTGGTAG	8057276	8057295	GAGCGTTGATTGAAGTCTGC	I
A33chr8-4	8A	14426201	14426220	GGGAAGAAGAACGGGAACTA	14426541	14426560	GTCATCGAAGCCATTTAACG	I
A33chr8-5	8A	18786042	18786061	GAACGGAACGGCCTACTTTA	18786352	18786376	TGTTAAATTTCTCATGCATTTCAAG	I
A33chr8-6	8A	29129012	29129038	TTTTTATTGTTGGATAAGGGTTAATCT	29129360	29129383	TGCTCCATGGACAAATTTAGTATT	IV
A33chr8-7	8A	32668762	32668786	TTGTATGTAGCTGATGGAAAGATTG	32669006	32669024	CGCTGCCCTAAAAGGAGTT	I
A33chr8-8	8A	37155266	37155287	CCTCGAGTCTTATCCTCAAAGA	37155500	37155525	TTGTCGTAAATAAGATACTTGAGGAA	IV
A33chr8-9	8A	41393225	41393247	GCTTCCGTTACTTAATTTGTTGC	41393528	41393554	TCCATTATAAACACAAGTCTAGTGAGA	I
A33chr8-10	8A	48905745	48905768	TCTTTGCTCAAACATTTCTGTAGG	48906159	48906184	AACCTACCCTATATTGGTGATACTGA	I

A33chr9-1	9A	1786529	1786553	CGGTTTGATTTACATTTCAATTTTT	1786718	1786737	GCTGCATGACTGAGAAGACG	I
A33chr9-2	9A	9672469	9672492	TGATGGTGTAACTGAATGTTTCAA	9672822	9672841	TGGATGATTCAATGGCCTAA	IV
A33chr9-3	9A	18362517	18362540	CAAGGTCATAGATACAATCGAAGG	18362793	18362812	TCGAATCAATTGGGATTTCA	I
A33chr9-4	9A	29730088	29730111	CAAATCAGCCCCATATATGTATTC	29730337	29730356	GGCATGCATGACTTGCTATT	IV
A33chr9-5	9A	47732018	47732043	TTTGAGAAAAATTAAGATAGGGAATG	47732313	47732339	CAGCTTAAAACTCTTTTCATAATCTTC	IV
A33chr9-6	9A	83339354	83339373	CTGTCCTCGGTCGTTCTCTT	83339755	83339778	GGAATACAATTATCGTCTCCTCCT	I
A33chr9-7	9A	89697445	89697465	TGGAGAGAAGAAGGGTCTGAA	89697726	89697750	TGGCTTTACATGCACTACTACATTC	IV
A33chr9-8	9A	98472659	98472683	TGCTTATCAAATCTCGTACTCATGT	98473025	98473044	CATGTGTCAAGCGGGTAGAA	IV
A33chr9-9	9A	106253760	106253781	CCGGTGATGTAAGAAATGAAAA	106254027	106254047	CCCTTACAACCACCAACAACT	I
A33chr9-10	9A	113366236	113366256	TTTGTTTGGGACTTCCACTTC	113366437	113366458	AATCACAACTGCCCATATGAAA	IV
A33chr10-1	10A	329167	329188	ACCCTCTATGGCTTCATTTACA	329408	329427	GAGAAAGCCAGGTCCTTTGA	I
A33chr10-2	10A	3936198	3936221	AATGTTCCTGTTCAGTGTTCATAA	3936513	3936533	AGTGGAGGGGTTAACTTCAGA	I

A33chr10-3	10A	11174112	11174133	GGCAAGTCTCAACTCTCACCTT	11174544	11174567	AATTGACTGATATGACATGCAACA	I
A33chr10-4	10A	21860978	21860997	TCCCAGCAATGGATAACACA	21861163	21861187	AGTTGACTGATAAAATGAGCGACTT	I
A33chr10-5	10A	31151148	31151173	CGAAACATATGTATTACCATCTAAGC	31151513	31151532	AACTGACACCTTGCAGCAGA	IV
A33chr10-6	10A	72099332	72099352	ACATGCAGGTAGGTTTCCATT	72099545	72099566	TTCATTTCACAGTCATCATCCA	IV
A33chr10-7	10A	79266230	79266255	TCCTGTATCCTTCATCTTATTGTAAA	79266742	79266761	GGCTCAAGCTCTCTTTGGAT	IV
A33chr10-8	10A	92371207	92371229	TGAATGAATACCCTGACAATAGG	92371574	92371592	AGATGATTGACCGGCCTCT	I
A33chr10-9	10A	105085786	105085809	TGGTACTTGACACTTGGATAGCTT	105086150	105086169	TTGGTTGCGTTGTTGAGAAG	I
A33chr10-10	10A	114015825	114015844	TTTCATTTCCCCAGATGCTC	114016055	114016077	TCTGATTGAGTTGATTTGGGTCT	IV
A33chr11-1	1B	3811934	3811951	AACCACCCATGACCCTGA	3812232	3812250	GCTCTGGTCCAGTGGTTTG	I
A33chr11-2	1B	6382769	6382788	TTCTTTAAACGAGGGGCAGA	6382967	6382986	TCAATGTGTTTGGCGATTGT	I
A33chr11-3	1B	7849884	7849910	TTAAATGATTAAAATCTGAAAACCTTG	7850147	7850170	TTGTTTCATTGTTATGGGAGTTTT	I
A33chr11-4	1B	8004363	8004389	AAATATGTATCTGTATGTGTTCGTTCG	8004609	8004629	TCTTCGTCGAGTCCTCAATTC	IV

A33chr11-5	1B	12279538	12279557	CCATGACAACCATGTCAACC	12279736	12279757	CCTTCTCCTTAAGCTCCCTTTC	IV
A33chr11-6	1B	18015521	18015547	TCTCTAGCATTACTCATTCATAAAACA	18015738	18015761	CTGAATTGATTGTTTTCTAACTGC	I
A33chr11-7	1B	32774523	32774545	CCAATTCAAGAGTGATTGTATCG	32774779	32774800	GCCCATTTAGCTAGACTGCTCT	I
A33chr11-8	1B	33187245	33187268	GGCATTTGATTCTTGTTCTAATTC	33187446	33187467	TAGAAGATGGGAAATTGAATGG	I
A33chr11-9	1B	53481520	53481542	TCAATAGAGGAGAAATGGGATCA	53481957	53481976	CGAAGGATCGAATCCAGAAG	I
A33chr11-10	1B	120035035	120035061	TTCAACAACTTTAAAATCTCCATTATT	120035434	120035455	TTGTTTTCACTAGAGGCACAAA	I
A33chr12-1	2B	1516751	1516770	TTGCTGAGCTGCGAATACTG	1517187	1517210	TCAATGATCTGCAGCATAATAACA	I
A33chr12-2	2B	4396453	4396477	GAAGAGTAAGGTGCTTTAATTGGAA	4396648	4396668	CACCTATGTTTGAAGCCCAAG	IV
A33chr12-3	2B	5627811	5627831	AGCCATTTCTCTTTGTTGTTG	5628166	5628186	CCGCATGTGAACCTAATAGAA	I
A33chr12-4	2B	19050214	19050239	TGGAATAAATTGAGGAAGAATTACAA	19050683	19050709	AGTTTCTATGCTGCTACTATGTTTTGA	IV
A33chr12-5	2B	22826587	22826608	CAAGATAGAGCATGCAATTCAA	22826787	22826806	GGGGTGGAATCTTTGTTTGA	IV
A33chr12-6	2B	87850773	87850790	AATGCAGATCGGCAGGAC	87851139	87851158	CCGAACACAACCCCAATAGT	I

A33chr12-7	2B	104289449	104289471	GCTGCGTGTTGTGTTATTTGTAT	104289750	104289774	AAGATTGAAACAGCTTTAAGTTTGC	I
A33chr12-8	2B	104780480	104780500	TCGCTTTTATTTGCATGTGTG	104780828	104780850	ACACCTCCTCTGATTTTCTCTCA	IV
A33chr12-9	2B	111678731	111678750	AGAATGCAGGCCAAAACAAT	111678995	111679015	CTATAACACGTGTCGCGTCTG	IV
A33chr12-10	2B	113911337	113911358	CTTGGAATTGAAGCAACTCTTG	113911757	113911783	CAATTACTAATAGGGATTCACTTTGTC	II
A33chr13-1	3B	6559460	6559480	TCGGTCAATCTTTAACGCATA	6559654	6559679	CAAGTATTTGAATATGACCAAGACAA	IV
A33chr13-2	3B	16093895	16093920	AATCAATCATATATACTTGCGTGTCT	16094136	16094160	GGGTAGTTCATAGTTGTTTAATTCG	I
A33chr13-3	3B	35087580	35087606	GATAGATATGAGTTCTAGCTGACAATG	35087960	35087979	GAACAGCAGTTGATCTGCAA	IV
A33chr13-4	3B	83054506	83054525	AGCTCACACATGAGGCAAAC	83054798	83054823	CAGATTTTAGAAGCTCTAATCAGTGG	I
A33chr13-5	3B	118922778	118922797	TCGTGTGAGCTTTGTGAGGT	118923269	118923292	GGATGTAATGGGTCTTCTTATGGT	I
A33chr13-6	3B	134298771	134298796	GGTAGTTCACTTTGGAGTTACATTGA	134299253	134299273	TCAGTGGAGCCATCTTTAACC	I
A33chr13-7	3B	137735803	137735824	CACCGTTGCTAAGTCCTCTCTA	137736049	137736068	TTGGTCTTTGGAACGAATTG	IV
A33chr13-8	3B	139429687	139429708	CAAATAGCAACAATGGTCGTTT	139429979	139430004	TCACTACGACCCTACTCAATAAGATT	I

A33chr13-9	3B	140720774	140720793	CCGACTCCTAACCCTAATCC	140721083	140721107	GAAGTCGCTCTCCTAGTTAAGTACC	I
A33chr13-10	3B	143323355	143323381	GATGTTATATTATATCTGAGTCCACGA	143323644	143323663	CGAAGTATCCCACGTTCCAT	I
A33chr14-1	4B	1542545	1542571	TTCTTTTACAATCTTTCATACTCTCAA	1543021	1543047	ACTGATTTGTACAAGTAGGACTAGGAT	I
A33chr14-2	4B	4514285	4514308	GATCATATATTCATGTCCATGTGC	4514605	4514625	TGACATGCCAGAAGAATTTTG	I
A33chr14-3	4B	4639343	4639369	TGTGTTTTGTTAGTTAGCCTTATTGAA	4639832	4639851	GTGTGATCCTCGAATCATGG	I
A33chr14-4	4B	24900834	24900858	CCAAATAGTCCTAGACTCCTAGCTC	24901046	24901066	TTCCTTCATAAAATCCCTCCA	I
A33chr14-5	4B	98132847	98132873	AGGGATACTAAAATAGATCAGAGATGC	98133090	98133109	ACCATTGCTGGAAACCACTA	I
A33chr14-6	4B	109312214	109312234	GGGTGGGTTTAGAGTTAGCTG	109312484	109312503	CGTCAAACTCTTCCGCTTCT	I
A33chr14-7	4B	124361331	124361357	AAAGATCTATTATTCAAGAGTCGCATA	124361538	124361559	TCCCATACGGTTTGAAGATATG	IV
A33chr14-8	4B	125061097	125061116	GCCATTCGTCAAACTGAACA	125061442	125061463	AATTTTGATGGTGCTGATGTTG	IV
A33chr14-9	4B	134418710	134418735	TTCAGTTAGTTACATCGGCTAGAGTC	134419016	134419041	TGACGTATCGATAACATATTCTTGTG	I
A33chr14-10	4B	139058541	139058560	GTGCAAACCGCAAACTACAA	139058916	139058942	GATAGTAGGGTAGTGAAGTACCTGAAA	IV

A33chr15-1	5B	9182277	9182299	GCAGGTTGAATTTGTATTTGAGG	9182588	9182608	ACTTCACGATCACATTGAGCA	I
A33chr15-2	5B	16873292	16873313	GGGCTACGGGGTCTTAATTAGT	16873708	16873727	AGGAAATGACCGACACCTTG	I
A33chr15-3	5B	19208389	19208413	CAGTAAATTGTGAATCACCCTCTTT	19208764	19208786	GGATGCAGATAAATACTGCGAAT	I
A33chr15-4	5B	20771601	20771622	TTCCACTCTCTTCTTTTCTTGC	20772035	20772059	TGTCAAGTCATGTCATTATGCTATT	IV
A33chr15-5	5B	25489569	25489592	AAAAACAAATCACCCAGACTATCA	25489946	25489970	AAGGATTATACAAAACACGGATTTC	IV
A33chr15-6	5B	26762773	26762796	GCACACTCTAGATTTCAGGTCTCA	26763111	26763132	GCATGTTCCTAGTGAGCTTCAG	IV
A33chr15-7	5B	73399881	73399901	CATTCATCTAGAGGGCTGCTG	73400170	73400191	AAGGCAAGATAGAGTTGGCATT	I
A33chr15-8	5B	100759514	100759534	CAATTGAACACGACCTTTTCC	100759819	100759842	TCAAATGTACTTAGCAAGCCAAAC	I
A33chr15-9	5B	133373533	133373559	GCAAGGTGATATTAAATGTAAATCTGT	133373788	133373807	GTCACCCATCACAACAGGAG	IV
A33chr15-10	5B	138577748	138577767	TTTCTCTCCATGCGACTGAC	138577965	138577990	CATGCTATTGACCTTAGATATGATGA	I
A33chr16-1	6B	1670486	1670504	CCGAAATTGGTTGTCGTTG	1670711	1670736	CCAATCATTTATCTTTTCCTTAGCTT	IV
A33chr16-2	6B	9569255	9569275	TGTGCCTCAATTCTCTGATTG	9569752	9569773	CCAGCCAAACTAACAACTCATT	IV

A33chr16-3	6B	56712272	56712296	TCGATAGATTCACCTATCAACACTA	56712701	56712720	CCAATTGCTGCATTTCATTA	IV
A33chr16-4	6B	126944469	126944491	TGATCTTTCTCACTCTCTTGGTG	126944715	126944737	CCACTCTCTCATTGTAACCGTAA	I
A33chr16-5	6B	132399813	132399837	TCAGATGGATTAAGTTTCTTGTGTG	132400039	132400062	AACAAGCACACTAAAGGATACGAT	IV
A33chr16-6	6B	139941757	139941782	AAAGCAACAATAATTACATCACACAA	139942090	139942109	TCTCAGAACCTTGACCCAGA	IV
A33chr16-7	6B	144323421	144323440	GAAACAGTCGAGGCCCTAAG	144323739	144323765	CAGAGTAATCAGCTAACAGAATAATGA	IV
A33chr16-8	6B	145333230	145333254	TTGATATTTCGAATCTTATGCAACA	145333662	145333683	TCTTAAACGAGGGAGAAGCTGT	IV
A33chr16-9	6B	146670771	146670797	TCGACTCGTTAAGCTGTAAAATACTAA	146670980	146670998	GTTGTGGCTGGCTTGACAT	IV
A33chr16-10	6B	147714778	147714800	AGGTATATGGTTCGCTCTATGAA	147715177	147715198	AACATAAAAGGCACAGTGAGGT	I
A33chr16-11	6B	6710089	6710111	CAGTGTTCATGAGGCCTTAAAGT	6710285	6710304	AACCTGCCCAATTCACAAAG	I
A33chr16-12	6B	15355486	15355512	TCCATCCAGTTTAACTAATATTTTTCT	15355850	15355874	AAATGAATTAAATTGGTGATGAAAG	IV
A33chr16-13	6B	20242081	20242103	TGAAGTTCTGTTAATCCCTCGAC	20242303	20242322	TCACTCTCCGTCCCTACTCC	IV
A33chr16-14	6B	124407351	124407370	CAACTGAGCACTCAAATTGC	124407595	124407614	CATTTCTGTCACCAGCCATC	I

A33chr16-15	6B	129377487	129377510	TTTATCACAATTCTGTTGGGTAGC	129377927	129377950	TTCTCTTATGAATAGGCTCACCAG	IV
A33chr17-1	7B	508703	508722	TTAACATACACCCCGCATCA	508946	508965	ACCAGAATCGTACCCAAAGC	IV
A33chr17-2	7B	1544457	1544475	ATGCGCCATGAGTCTTCAA	1544695	1544719	AAACAAGGGATAAAATGAACAGAAA	IV
A33chr17-3	7B	7825292	7825318	TGGTATTTGGTAGAAACACTACATTTG	7825515	7825538	AAGTGCTATAACCTTTGGCAATTA	I
A33chr17-4	7B	20199402	20199421	CCAACCGATGGAGTAAGGAG	20199870	20199893	GGTTTGAAGCAATAATAGCAAAAG	IV
A33chr17-5	7B	40362901	40362923	GCAGGAATCATAACTCCAATAGC	40363219	40363238	GCGACTTGTTATGGGAGGAG	IV
A33chr17-6	7B	42192429	42192448	GGAAGCCATCCCAATCTACA	42192899	42192920	TTTTACCCAAGTCACATTTTGC	IV
A33chr17-7	7B	73365466	73365487	GAAACAGTCACACAGAAGAGCA	73365746	73365767	TCCATGGGCTAAGTATTGCTAC	IV
A33chr17-8	7B	79097221	79097240	AGAAGCTTTCGCATTTGACA	79097538	79097564	TCCATAAGTTTGTTGATTAATTGTAAA	I
A33chr17-9	7B	105174076	105174099	CACACTACCCCATATCGATCTACT	105174410	105174435	TCAATAGTAATGGGGATAACTTGATT	I
A33chr17-10	7B	131641785	131641808	TCGGTCTTCAAAGTATCACTTCAC	131641967	131641992	GGTCATTACCTATTAGTGATGTTTGC	I
A33chr18-1	8B	3370267	3370293	GTATTCCATTGAAAGTTGTTTAATTTG	3370600	3370619	CTGGTGGAACTTCCTTCAAA	IV

A33chr18-2	8B	5149876	5149895	CCATGCCATGTGTTTCTCTT	5150297	5150317	TGCAGTCTCGGTTTCTATCCT	IV
A33chr18-3	8B	6576803	6576821	ACCCGCCATAGCTTCATTT	6577106	6577125	AAGTCGGATTGCAAGAGAGC	IV
A33chr18-4	8B	14032901	14032920	GTTGCCCACCTTTCAACATA	14033349	14033374	TTGGAGCTATTAAAGAGAAAGTGAGA	IV
A33chr18-5	8B	16768123	16768147	TCCTCACCATCTCAGATTACATACA	16768324	16768344	TGCCTTGTGTTTTAAGGTTCC	I
A33chr18-6	8B	19679264	19679283	CGTTCCCTCCGAAACTTCTA	19679486	19679512	AAGTTTACAGTCTTACCACAAGAAAGA	IV
A33chr18-7	8B	24419681	24419700	AGCGCTACCCGAGTTAGTGA	24419924	24419947	GACAATTGGATCAAAAGAAGTTGA	II
A33chr18-8	8B	30034533	30034558	TGGTTAGAAAGAAACCTAAGATGTTG	30034966	30034985	GCGATGTAAAATGCACAAGC	IV
A33chr18-9	8B	111590479	111590498	GGCCAGACAAACAAACAAAT	111590747	111590766	TCGGAGTTAGAGGAGGAGGT	I
A33chr18-10	8B	130445279	130445304	CACTCCACGCGACTATATATCTTAAA	130445635	130445658	AGTTACATCTAACAAGGGTGCCTA	IV
A33chr19-1	9B	380118	380138	ACCCATTTCTGGGTTAATCAT	380366	380385	AGGGCAATCAATCAAAGGAC	IV
A33chr19-2	9B	21443189	21443208	TTGTCCTGGGCAGGTAGAAC	21443430	21443449	GATGCCACATCAATGTCGAG	I
A33chr19-3	9B	22108284	22108304	AGCATCTAGCATGAGCATCAG	22108482	22108504	TTTCTATGCTTTTGGTGTTCTGA	IV

A33chr19-4	9B	38830519	38830539	GCAAAGTAGCAGTTGGCAAAG	38830775	38830795	GGAGGTTGGATGAAGTTGGTT	IV
A33chr19-5	9B	52410999	52411022	CCAACCTATCTTAACTCCAACTCC	52411238	52411262	TCATCTATCAAGAGAAAATGGAAGG	I
A33chr19-6	9B	53296428	53296452	AAAAAGGGATTTTTCTAAAGGTTTG	53296902	53296923	CCAGAGAGCTTGCACTTAATTC	I
A33chr19-7	9B	97717694	97717719	TGCTCTTGGCATTAGTTATTATTTTG	97718055	97718074	TTGGCCTACGAGCATCTTCT	IV
A33chr19-8	9B	98606545	98606565	TCATCCCCATACAGGCTACAG	98606745	98606765	CAGTGGTAAATTGGCAATGCT	I
A33chr19-9	9B	114107875	114107896	GGCTCATCAAAACTTCATTTCA	114108146	114108165	GGTTGATTTGGTGTTGCAGA	I
A33chr19-10	9B	136323077	136323097	TCTTCTCGTCCTTTCCTTTCA	136323435	136323454	GCGTGAAATTGTTGGGATTT	I
A33chr20-1	10B	4262518	4262537	AATCCATCCATCCATCCATC	4262770	4262789	GGAACCACCGTAATTCTTCG	IV
A33chr20-2	10B	7770952	7770971	AAGGAGGCGGAGATGAACTA	7771214	7771234	TGCAGCTCACACCTATCTCTG	IV
A33chr20-3	10B	13987716	13987740	CATTTAAACTCACTGTCTCACTCCA	13987970	13987996	TGAATTGTGAAGTATTTTAGAAGCAAA	I
A33chr20-4	10B	39337850	39337870	GCAAGAAAGAACGAAACAGGA	39338068	39338087	ACTGCTTCAGATCCAACACG	IV
A33chr20-5	10B	105643895	105643919	GGCCTTGAAATGTTTATATATAGGG	105644216	105644235	CTCCTTCACCTCGTTCTTCC	I

A33chr20-6	10B	121723252	121723271	GCTATTCATGCCTCAAACCA	121723532	121723556	AGTGAATGAGATGAAGATTGATTTG	IV
A33chr20-7	10B	132235308	132235327	ACTCCCATGGACACAACTCC	132235516	132235535	TGACAGAGAGGCTACGGTTG	IV
A33chr20-8	10B	139697378	139697400	CAATGAGCAGTACATGGGTATGA	139697600	139697625	TCTCCTTTTATCTTGCATACAGATTT	IV
A33chr20-9	10B	139966595	139966621	TACACATATTACAAATAGAATGCAACA	139966930	139966949	AATGCAGTGCAGGATTGTTG	I
A33chr20-10	10B	141584326	141584349	TTTGAATTCTAGCTACCAACCGTA	141584595	141584621	TGCATACTCAAATTTATAAAACTCAAA	IV
A33chr20-11	10B	4481072	4481091	CAAAGTGTGGAATGGCTGAA	4481313	4481330	GCGTGATGAGAAGCTGGA	IV
A33chr20-12	10B	10968384	10968406	AAGAGATTATGTTATGGCGGAAA	10968777	10968800	TTCAAATTCAATCTCTCCATTCTG	I
A33chr20-13	10B	112537181	112537202	TTCCTTGTTTTTCAAAACTCCT	112537653	112537673	CAAGGGGGATAGTTTGTTTTG	IV
A33chr20-14	10B	143462127	143462150	TTGAGTTGGAGACTAGAAAGGGTA	143462595	143462614	CCCAGCAGTGTGTCAAGAAA	IV
A33chr20-15	10B	143920360	143920386	ATTATGTATCAATTGTGTCAATGAAAA	143920718	143920737	ATCCACATTTGTCCATCACG	IV
G2chr1-1	1A	5491452	5491470	AGTTGCGGCGGAGTTATTT	5491650	5491669	AAACCGGAGAAGACGAAACA	IV
G2chr1-2	1A	8541411	8541437	GATGAGAAATAGTTTAAGAGAGCTTCC	8541660	8541679	AAGGGTGCCTCGATTACTCA	I

G2chr1-3	1A	18407063	18407082	TTGCCACGACAAGCAATAAT	18407331	18407350	AAAGCAACGAGTCAGGCAAT	IV
G2chr1-4	1A	51240036	51240060	AATATAGGTGTTCACATGCAGATTG	51240511	51240535	GGCAGATTTAAGCTAGTACAACTGG	IV
G2chr1-5	1A	93642587	93642613	TTGTCACTCTTCATAATTCCTTATTCA	93642878	93642899	CCTCCATTTTCTTGAGTTAGGC	IV
G2chr1-6	1A	93642733	93642753	ATGGGAATGCATGCTATAAAA	93642938	93642957	TGAATCCAAGGAGCGGTAAT	IV
G2chr1-7	1A	93643110	93643136	TTCTTAGTACTATTTCTTACCGTGCTC	93643482	93643507	AACAATTGTCATCTCTCTACCTACCT	IV
G2chr1-8	1A	96923717	96923743	GATCTTATTACATCTATCCAACTGTGC	96923961	96923980	ATTTTCCCACTTGGTTGGAT	IV
G2chr1-9	1A	106731477	106731502	CCATATACTCAAACTCAATACCCAAA	106731767	106731789	CCAAACCCTCACTAAAAGATTCA	I
G2chr1-10	1A	106731484	106731506	CTCAAACTCAATACCCAAACTCA	106731811	106731831	AAAGGGAAACACGCTAAAACA	I
G2chr2-1	2A	6150728	6150747	TTCAATCTGCCATGGTTCAC	6151031	6151052	CCACCAAAGATATGCTATGCAA	I
G2chr2-2	2A	6880229	6880248	CATTGGACTCACAGCACCAT	6880617	6880637	ACGCTTGTTATTTGGACGTGT	I
G2chr2-3	2A	6880238	6880259	CACAGCACCATGTGAGAATAAG	6880618	6880637	ACGCTTGTTATTTGGACGTG	IV
G2chr2-4	2A	24222597	24222622	GTTATCTTATCTCGTTCTCACAGTGC	24223040	24223059	ACCGGAAGCCAACATGTACT	IV
G2chr2-5	2A	37607588	37607614	CCTGACCTTAATCTAAGAAA	37607795	37607814	TGACAAGCAATCAAGGGAG	IV

				ATTAGAA			A	
G2chr2-6	2A	45104723	45104747	TGCTCTGTATTTCTGTAGATGGTTT	45105179	45105203	GGCAATAGGAGTAAAACTAACATGG	IV
G2chr2-7	2A	67498669	67498690	TGACAATATCCGACGACTTTCT	67498854	67498873	TCACAAAAGTCACCCATCCA	I
G2chr2-8	2A	86430571	86430590	GGGATTAGCCTGTCACCAAA	86430960	86430984	TCACTCCTAACTCCACTACTCACAA	IV
G2chr2-9	2A	88868724	88868748	TCTAGTAATGATTCGATGATGGATG	88868955	88868977	TTGTCTCAGAAAGTTCCAATTCA	IV
G2chr2-10	2A	81444266	81444290	TCTATGATCAAGGAAAGAAAGAACA	81444685	81444704	GACCCAAGAATCAACCCAGT	I
G2chr3-1	3A	2904898	2904921	TAAAGCATAAGTTAAGCACCCAAA	2905326	2905348	TGTGGAAGTACATTTGGTTTACG	IV
G2chr3-2	3A	3642054	3642078	ATTAGACGAGAGCTAAGTCCAGAGA	3642258	3642277	TGGAAAAGCTGGTTGGTAGA	IV
G2chr3-3	3A	6556785	6556803	CATGCTGATGGTGGTGTTG	6557096	6557115	GACCCGGTCACATACTCTCC	IV
G2chr3-4	3A	17169160	17169183	GACTCACTCCAGTTCATCTCTCAA	17169368	17169391	GGTAGCAATTATGAAATAGCCAAG	IV
G2chr3-5	3A	31465493	31465515	TTGTCCTAGCTGCTATTCTGAAA	31465823	31465842	ACGTTTGTGAGCATGGTTTG	IV
G2chr3-6	3A	37381437	37381463	CAGATTAATTCTTATTCTACCGAGTTG	37381830	37381849	AATCCACCCATGAATGACAC	IV
G2chr3-7	3A	121311987	121312006	GAGGGCATGGTTCAAGAGAC	121312514	121312533	ACCTCGCTCACACAACACAT	IV
G2chr3-8	3A	138007244	138007269	CGGATTCAGTAAATGTTTTTA	138007455	138007479	TGCTGTCAAGTATGTGGTTTA	IV

				ATCTT			ACTC	
G2chr3-9	3A	140231705	140231724	TGGGCTGATTAGCAAGCTCT	140232222	140232243	TGGAATATCAAGCATCAAAGGA	I
G2chr3-10	3A	141940259	141940284	CGAAATTTTCATTAAATTAAGTGTGG	141940608	141940634	TGAGAATATGTTGTGTAATGCAATAAG	IV
G2chr4-1	4A	9765660	9765679	AACTCAACATGGGGATTTGC	9765961	9765981	TGACAGAACCTCATCAAAACG	IV
G2chr4-2	4A	28264285	28264309	TCATGTTAGATTTATGTTGTCATGC	28264725	28264744	CTCAATTCGCCACTGGTCTA	IV
G2chr4-3	4A	72128394	72128413	TGTGGTTCTCTCGCTGTTTG	72128651	72128673	TGATGCATAATTCTTCCATTTCA	I
G2chr4-4	4A	110306386	110306407	GAAGGCATCAACTCCTTTATCA	110306613	110306632	TTCCACGCAGAAACTCATTG	IV
G2chr4-5	4A	120079207	120079231	AAAGGATGATGAATACATTTCTTGG	120079594	120079620	TTCATAGGAGCTAATGTTAATGTTTCT	IV
G2chr4-6	4A	123381061	123381085	AACACTAACACTCTTTCTTTCCTTG	123381396	123381415	TGCAGTGTCTCGAGGGAGTA	IV
G2chr4-7	4A	123396934	123396954	TTTCTATCCTGCAACGGCTTA	123397398	123397417	TTTTGCTGCACTCACAAACA	I
G2chr4-8	4A	123432066	123432089	TCAAGTGATTTACCTGAATGGTTT	123432415	123432435	TTCATGAAATTGGGTTTGAGC	IV
G2chr4-9	4A	123436521	123436546	TTGTTCATTATGACATTTCTCATTCA	123436775	123436795	AGTCATTGTTTGGCTGAATGG	IV
G2chr4-10	4A	123511202	123511225	GCATTCTACCCTTAACTTTGTTCC	123511526	123511544	CTTGGGTAGGGTGCGTTTT	I

G2chr5-1	5A	11355924	11355946	TCCCTCCAATTATAACATCCTTG	11356170	11356195	CAGTTATTTCTGGATTGTACATGGAT	IV
G2chr5-2	5A	11358776	11358800	CATCTCCATTATGATTTTAGCCTTT	11358954	11358974	CCTCACCAATAACGGAAATCA	IV
G2chr5-3	5A	11422657	11422683	AAAGACTTGATTTTACCATACCCTTTT	11422866	11422889	CAATCATGTTATGGACACACAAAA	IV
G2chr5-4	5A	11971438	11971459	GGAGTTCTGAGTGATTTTTGGA	11971878	11971897	CCCATTTCCCCACTCACTAA	IV
G2chr5-5	5A	27470553	27470578	AAACTAGTAGACGAAGTAGACGATGC	27470927	27470951	AAGGTATCTTAAGGTTTTCACAGCA	IV
G2chr5-6	5A	36181076	36181100	CGACCTGCATATATAACAAATTCAA	36181312	36181331	AAGGAACCCTTCGATGTGAA	IV
G2chr5-7	5A	36184497	36184523	TGTAGAGGAGAAAGTGATTGGTTTAAT	36184709	36184728	AAACTGAAAAGCAACGCACA	IV
G2chr5-8	5A	36185179	36185204	GACAAGATATATTTATCACCATGCAA	36185552	36185571	ACAACCAGCGAATGTGTAGG	I
G2chr5-9	5A	90244559	90244582	CAGCTTACCTTTTCTTTCATCCTT	90245024	90245043	AAAAGATTCAAGCCCATTCG	IV
G2chr5-10	5A	108605578	108605602	CAGTGTAAATTGTCAATCCTTTGTG	108605773	108605792	TCCAGCCAATGAAGAGTCAA	I
G2chr6-1	6A	378118	378137	TGAGAGCTTTGGTTTGGTGT	378297	378316	TTGAAGCCATGCTCTGTTTG	IV
G2chr6-2	6A	1220855	1220878	TCTGGAACCCTTTTAACACTTTTT	1221208	1221228	AGCCGACTGCACTTAAGTTTT	IV
G2chr6-3	6A	1782171	1782190	AGAAGCAGCAGAACCAGAC	1782429	1782452	CATGATGATTCGTTTACACAG	IV

				C			ACA	
G2chr6-4	6A	1782307	1782326	AGTGGAGAGAGAAGGGGTGA	1782728	1782748	TTGGAGTTTTACAGTCCATGC	I
G2chr6-5	6A	3783222	3783241	GGTGACGATGAATGACTGGA	3783447	3783473	ACATTGACCTTATTTGTATTTAATTCG	IV
G2chr6-6	6A	38834073	38834094	GCCATGATAAACTTGGGTATGA	38834362	38834381	CCAGCCATAGCCACATACAT	IV
G2chr6-7	6A	53144118	53144139	CCCGTTATTCATGTTGTTCAAA	53144385	53144404	TGCATGAAGGATGGATGTTT	IV
G2chr6-8	6A	95417675	95417696	TTGGATTGGTCTAGTGGTTAGC	95417940	95417961	TGCCATCATTGATTTCTTATCC	IV
G2chr6-9	6A	105287833	105287859	GGATTTTGTTAGTGTGTAGATTGACTG	105288319	105288341	AGACCCTTTACAATCTCCAATCC	IV
G2chr6-10	6A	115124553	115124577	GCTGGCTAGAAATATCTCTTCTCTT	115124959	115124980	GCGACTACCATAATGAGATTGG	IV
G2chr7-1	7A	15576465	15576488	TTTTGATCTTTGAATCTTGTGCTT	15576669	15576687	AATGTCCCATTCAGCCACA	I
G2chr7-2	7A	16891970	16891989	TGTTTGCGAGGTGTACATGA	16892422	16892441	ACGGTTTCTGGCCTAAACAT	I
G2chr7-3	7A	60102815	60102838	GAACCAATCCAACTATCTTCTTCA	60103039	60103063	TCGTCATCAGTATAAGTCAGTAGCC	I
G2chr7-4	7A	67401805	67401829	GCACATAGAAGTACACACCATGTTA	67402242	67402268	AGTTTGGAGTAGTTAAACCCTAAACTC	I
G2chr7-5	7A	76671009	76671035	TTAAATTGCATATAAAACAACAAGAGA	76671239	76671258	GATATCCATGCAAACCCAGA	I

G2chr7-6	7A	76779123	76779146	CCACTAGCCCAACTATGAACTATC	76779326	76779346	TTGGGAGATGTTAACTGAGCA	I
G2chr7-7	7A	76779128	76779150	AGCCCAACTATGAACTATCTCAA	76779641	76779662	TCTCAATGCTGTCCGTTATTCT	I
G2chr7-8	7A	78037142	78037163	TGCCCAATAATTTGTATTTTCA	78037378	78037397	GAATATCGCCCATAACCAGA	I
G2chr7-9	7A	79026507	79026526	ACGGATTTTCACCAATGAGC	79026974	79026998	AGTCTATACGATTTGAAGCAAGCTC	IV
G2chr7-10	7A	79027972	79027992	AATGGCTGCCAAGACTTGTTG	79028305	79028326	CTATATTCGGCTATGCAGACCA	I
G2chr8-1	8A	6743483	6743508	AATCACAAAAGTGGAATAAATTTTCA	6743772	6743791	TGGAGAAAACGAGGGAAGAG	IV
G2chr8-2	8A	6804106	6804128	GCCAATGATGTAACAAATAGTCC	6804794	6804820	GAACATGACGACTAGTTTTAACTTCTT	I
G2chr8-3	8A	17511547	17511565	GCACATTTGATGGATGTCG	17511930	17511952	GATTTCTGCACCAAAATTAAAGC	IV
G2chr8-4	8A	18068847	18068873	GGTTGATCAGATAATAATAATGGCAGT	18069435	18069454	CGCAATACTCGGAGAGCATA	IV
G2chr8-5	8A	18473468	18473494	ATTATGAGGTTTGATAAGATAAAGCAT	18473877	18473895	TATCGACGCGCCTTATGTT	IV
G2chr8-6	8A	19300674	19300696	TGCATGAGTTTGGTTTAAAACTT	19301145	19301164	TTCGTCCACTTGTCCCATTA	IV
G2chr8-7	8A	19309199	19309220	AGAGCACAGTAAAGATGCATGA	19309626	19309647	TGTCTTGAGAGTGTGTTTGCAT	IV

G2chr8-8	8A	19337769	19337790	TTCTCTTCTCGTAGCGTAGCTT	19338008	19338029	TGATGATTTCTTGATAGGCAAA	IV
G2chr8-9	8A	25283108	25283127	GGTTGTTGAGGCATGCATTT	25283317	25283336	ATGCACCGAGATTTTGAGGA	I
G2chr8-10	8A	46007980	46008000	CGACCTTGTAACCAATGAGGA	46008345	46008365	CTTGTAGCCGATTTGGATGAC	IV
G2chr9-1	9A	7672028	7672050	GCTCAGATAACATAAACCACTGC	7672232	7672252	TTGATTGATTCGACCTGATCT	I
G2chr9-2	9A	8049631	8049649	TGCGCTACTGCCTTCTGTT	8050048	8050067	GATTGAACGAGGGAAATGCT	IV
G2chr9-3	9A	45615058	45615077	CGTCATCGCTGTTATTGTCC	45615315	45615335	CGTGAATATGAGTTCCGTGTG	IV
G2chr9-4	9A	45619269	45619291	CAGGCTAGAGAAGTCACCAAAAA	45619631	45619654	TTTAAATATTTTCGTTGCATGGTT	IV
G2chr9-5	9A	106318540	106318566	TGCATCACAGACATACAGTAGTAGTTG	106318816	106318834	TCCATAACACCGCGTGAAT	I
G2chr9-6	9A	117682580	117682605	AAAGTTACAACTTACAAGAGCTGCAT	117682780	117682800	TTGGTGACTTAACCCTTTCCA	I
G2chr9-7	9A	117682780	117682800	TGGAAAGGGTTAAGTCACCAA	117683245	117683267	AGCTAGGTCAGATTGGTCTGAGT	I
G2chr9-8	9A	117700116	117700138	GTTCAAAAGATAAAGGAATGCAA	117700490	117700513	TGGTATACTCATCATAAGCCCAGT	I
G2chr9-9	9A	7672044	7672069	CCACTGCAATACAATCTATCATTCTT	7672236	7672258	TGTAAATTGATTGATTCGACCTG	IV
G2chr9-10	9A	106317741	106317760	CATGGATTTGGTCATCATGG	106318441	106318467	TTATTAACATGTACATCAACTGCAACT	I

G2chr10-1	10A	12095675	12095701	CACGAGTCCATAATACATAATATACCA	12095899	12095918	TCCGGATTACCATAGACGAG	IV
G2chr10-2	10A	29321035	29321058	CTTAACATTTTGGGCAGTAAACTT	29321438	29321459	GCAAGGGAAGAGTACACAGATG	IV
G2chr10-3	10A	80252187	80252211	TCCTAAGTCCTAAGACCTAACTCCT	80252484	80252504	TGTCATCGGAGAACATACCAA	I
G2chr10-4	10A	80252187	80252210	TCCTAAGACCTAACTCCTAACACC	80252508	80252527	GAAGGAGGTTCCGGTAGTGA	IV
G2chr10-5	10A	80253310	80253332	TTTTTCATGCTCAAAACCTAAAA	80253671	80253689	CATTGATCGCAAGCTCGTT	IV
G2chr10-6	10A	80270320	80270341	GCTTAGGACTAATCCAGCGAAA	80270575	80270594	TTGGAGTGTGGAACCATGAG	I
G2chr10-7	10A	89260247	89260273	GCATATGAAAACAGAATAGATACACAC	89260483	89260509	TGCGTATATACATATAGACTCTTCCTG	IV
G2chr10-8	10A	94175565	94175585	TTTGCAGGTGGGTTAATCTTT	94175882	94175904	CACAAAGGGAGGATTACTTTAGG	IV
G2chr10-9	10A	103725248	103725266	CTGACACCACCTTCGACCT	103725517	103725536	TCCACTTTTACCACCGCATA	I
G2chr10-10	10A	105123819	105123838	TTTGTTTGATCACCGCAAAT	105124251	105124273	TGCCGTATTTTAGTAGGACGTTT	I
G2chr11-1	1B	38124	38146	ATCATATCCTTCTTCATCAATGG	38387	38410	TGAATTCCTGGTTTTAATAGGTGA	IV
G2chr11-2	1B	23099206	23099232	GATACGCCTCTATATAAATTGTACACG	23099391	23099410	GCATCACAAGGCATTCAAAG	IV

G2chr11-3	1B	54133139	54133156	CTGGCGTGTCTGGGAAGT	54133324	54133344	AGTGCTCGCTAAGATGAGGAT	IV
G2chr11-4	1B	121886410	121886427	TTATCTCGCTGCCGGTTC	121886666	121886685	CGGTACTATGCAAGCAACGA	I
G2chr11-5	1B	129727555	129727579	GATGTTGATATTCATTTTGTTACGC	129727919	129727940	TGCAGTACCTAGGAGAATGTGC	IV
G2chr11-6	1B	144115885	144115911	TAATAATAAAATGGAATTCAGCATACA	144116244	144116266	AAATCCACTATTCAGTCAACCAA	I
G2chr11-7	1B	144130480	144130504	TCATTCAACTAGCAGAATCTTTACA	144130729	144130749	GAAATGTGCAGAAAAGGAAGA	IV
G2chr11-8	1B	144131040	144131063	CCTTAGGAGAGTCGATGTTTTACA	144131379	144131400	TGCAATTTTCCTTATTCCGTTA	IV
G2chr11-9	1B	144131264	144131287	AACTCAACACTAATCATTCCCCTA	144131551	144131570	CATGTCATCGTCAGCTTTCA	IV
G2chr11-10	1B	144132095	144132113	AGAGGCGCATGGAGAGTTT	144132361	144132380	ACCGAGCATACCATCGTTTT	IV
G2chr12-1	2B	7752850	7752873	CAATCCAAGACATTCCTAAGGTTT	7753148	7753171	GCAGTGAATACAGAGAAGGTTTGA	IV
G2chr12-2	2B	14753347	14753365	GGCTGTGCGAATTTTCAAT	14753584	14753603	TGTTTTCCATAAACCCCACA	IV
G2chr12-3	2B	18048762	18048788	AAAATTGCTCACTCAATGTCTACATAC	18049204	18049224	GAAGGATTGAATTCGCAGCTA	IV
G2chr12-4	2B	18048784	18048809	CATACACTTTAAAAATCCAAAACAGT	18049001	18049026	CCAAATTACATTCTAAGTAGACACCA	IV

G2chr12-5	2B	22649889	22649913	TCATTATATGTGTGAATGACCAACT	22650149	22650174	TGAATATTGAGGTTTAGATTTAGCAA	I
G2chr12-6	2B	29294847	29294865	GCCAACCACTCTCCCTCAT	29295308	29295330	TTGCTGTACTGGACACTCTGAAA	IV
G2chr12-7	2B	34433959	34433980	TTAGTGAAGTTTTAGGGCCTTG	34434344	34434363	CATGTCAAAGACTCCCCTCA	IV
G2chr12-8	2B	114989603	114989629	GCCTTGATTGAAAATCATATAAGTAAA	114989876	114989895	TCTCTAGCGGCTTTCAAATG	IV
G2chr12-9	2B	116955488	116955508	CCACATGCTTGATGATTGATG	116955776	116955795	CAATCACCGCCCTTTAATTT	IV
G2chr12-10	2B	116956417	116956435	CAGCGCAATCAGATCATCA	116956679	116956698	TGGAGACGTGAGGAAGATGA	IV
G2chr13-1	3B	8833461	8833485	CCGTTGTAGTACCAAGAAAACAATA	8833953	8833972	TGAATCATGAATGGGAAACG	IV
G2chr13-2	3B	14933215	14933235	TCTCACTCCTCACTCCTCCAA	14933685	14933706	TGCAATTATTGTGAACATGTGG	IV
G2chr13-3	3B	39983345	39983368	GGCACTCTTATGTACCCATATCAA	39983585	39983610	CCAAACGTTAAAGACTAACACCATAG	IV
G2chr13-4	3B	39984227	39984246	CACAGGCATGGTCCATTTAT	39984501	39984524	GAAAATTGAACAGAAGCGAATAAA	IV
G2chr13-5	3B	47100025	47100044	GGAGAAGAGACTGGGTTGCT	47100319	47100338	ATTGTCCTCACCAGTTGCAC	IV
G2chr13-6	3B	54696227	54696246	AGAGCATTCAAATCCATCCA	54696531	54696555	TGGAATATGATTACGAGTTTACGAC	IV

G2chr13-7	3B	131041964	131041984	TTAATCCCAAGATTTGCTTCG	131042428	131042446	GTTGGCAGTTGGCAATCAT	IV
G2chr13-8	3B	131042434	131042453	GCCAACTGCCAACACATTTA	131042679	131042704	GGAACAACTTAAAAGGCTAAGCTAAA	I
G2chr13-9	3B	136167158	136167177	AACATCGACACGGACACATC	136167407	136167433	AATGCAGTGTACTGTTTAAAGTTCTGA	IV
G2chr13-10	3B	136168173	136168195	AGAAGGTGAGATTTTGGTTGACT	136168617	136168638	TCAACAACTCGAGCCTATTCAT	IV
G2chr14-1	4B	4638396	4638415	GGGGATGGAAAATGTGTGAG	4638665	4638684	TGCCCACATAAGATCCCATT	IV
G2chr14-2	4B	4639006	4639029	TCATTGAGTGTGAGTATGTGACAG	4639286	4639308	AACGAAGAAACTGCATGATTAGA	IV
G2chr14-3	4B	4639339	4639361	TGCATGTGTTCTGTTAGTTAGCC	4639568	4639589	TGATGATTGCCTGTTTCAGATT	IV
G2chr14-4	4B	4640088	4640107	CCAGGTGACTGAACGTGCTA	4640277	4640296	TGGTGATCTGTCAAATGCAA	IV
G2chr14-5	4B	7787472	7787498	GAGAAATATTCATTTTGAGCCATACAT	7787619	7787640	TGCGTAAAACACCGATACACTT	IV
G2chr14-6	4B	9651712	9651738	TCAATCTTTAATTGAGAATAAGGGATA	9651987	9652008	TTCATATGTCCAATTTGTGTGC	I
G2chr14-7	4B	88710809	88710833	CATCCAGAGATGTAGAGAGAGAGAA	88710962	88710984	TGCCCTTATTATGAAATGTTTTG	II
G2chr14-8	4B	109564968	109564990	TCCTTTGCTACATTGTTGAGTTG	109565110	109565130	CGGGACAAGAGTTTTAAGCAA	I

G2chr14-9	4B	111044395	111044415	AGGTATCAGGCCAAAATGTGA	111044624	111044647	ACATGTTTGTAACGTGTGATGTGT	IV
G2chr14-10	4B	133091324	133091344	TTGGGATCATTAGGAGGCACA	133091917	133091943	GGTTTGTACTACTAATTGACCTCGTGT	IV
G2chr15-1	5B	9154246	9154265	CTTGTTCCTGTGGATGGTTG	9154572	9154593	ATATTTCCAATTGTGTCCTTGC	IV
G2chr15-2	5B	9182001	9182025	TGATCTACCTCCTCTCAATCTATGC	9182259	9182281	CCTGCACTAGAAGAGATTGGTTT	IV
G2chr15-3	5B	9182277	9182299	GCAGGTTGAATTTGTATTTGAGG	9182588	9182610	AAACTTCAGAATCACATTGAGCA	I
G2chr15-4	5B	26260247	26260272	GCTTAGTACGTAATTTTAGAGTGCAG	26260405	26260428	TGTAGAAATTACACGACTCCATTG	IV
G2chr15-5	5B	26428936	26428960	GCTAATATGCCACTTTACTCCCTCT	26429155	26429177	AATGAAACAAACCCAGCTAGACA	IV
G2chr15-6	5B	26472111	26472130	TTGTCTTTGCCTTTGCCTTT	26472325	26472350	TGTTTCTGCTTGCATACTAGATCTTT	IV
G2chr15-7	5B	26534270	26534290	TTCCTCCAATATCCAAACAGA	26534712	26534738	GTCTTTGCTATTACCAATGTAAATTCT	IV
G2chr15-8	5B	34818214	34818237	CCTGATTGATATCATATGCCTTCT	34818702	34818722	CCAGATTTCAACTTTGCGAAC	I
G2chr15-9	5B	73399866	73399890	TCAATGGAATTATCTCAAATTCAAA	73400174	73400195	GTTGAAGGCAAGATAGAGTTGG	IV
G2chr15-10	5B	144936659	144936680	CTCTAGTGTGTCCCATGAGTCC	144937086	144937105	CAGAGCAATGCCTCTTTGAG	I

G2chr16-1	6B	11307600	11307620	TTACTGGTTGTTGCGATCAGA	11308057	11308077	AGTCCTGTAGTCGCCAGATGA	I
G2chr16-2	6B	18801810	18801831	TGTTGGACTGACTGACTGACTG	18802065	18802088	GCATTTAAGAGTCTGTTGTTAGCC	I
G2chr16-3	6B	20049798	20049819	CCCCTTAGTCCTTTACATTGGA	20050282	20050303	TCAATCTGACTTCTGCTTCCAA	I
G2chr16-4	6B	111667590	111667609	TTTAACCGGTCAGGATGCTC	111667922	111667947	TCAAATTTTACTATTTTGGAGAAGCA	IV
G2chr16-5	6B	118739772	118739797	ACAAATTTATACGACACCTAGTCAAC	118740235	118740253	GACGCTGGAAGATGGATTG	IV
G2chr16-6	6B	128935052	128935072	CCCTACCCTACCCTACGCTAC	128935509	128935528	GTGAAAATGGATGCGAGTTG	IV
G2chr16-7	6B	137322841	137322864	TGCTTCTTATAACAATGGTCTTCA	137323171	137323197	CTACCATAAATCTATTAACAACCGAAA	IV
G2chr16-8	6B	138671799	138671820	CCATCGTAACAAGAACACTTGC	138672238	138672261	CTATAACAGCTTACTCTCCCCAAA	IV
G2chr16-9	6B	142819431	142819451	TCCTGCTTCATTGTCATTGTT	142819709	142819730	TCACTCTGAAATTAAGCGAACC	I
G2chr16-10	6B	100201391	100201409	CCATGGTCGTCGTCCTCTT	100202232	100202251	TCAGCCATGACGACTCAAAA	I
G2chr17-1	7B	20560446	20560471	TTATATCTTGACACATAGCATGATGA	20560862	20560885	TGAGATATTGATACTTGCGATGAA	IV
G2chr17-2	7B	25776468	25776491	GCTTTCTTCGAGTATGATTATTGG	25776671	25776690	TGTGGTGGTGAAGAACATGA	IV

G2chr17-3	7B	68075567	68075588	GATGATGACGATGATGATGATG	68075853	68075877	AAAGTAAACTTAGCATTGCAAAACC	I
G2chr17-4	7B	104854607	104854630	CTTTGAAAGCGGTTAATATGAGAA	104855060	104855079	TCACAAGGGATCAAACATGG	I
G2chr17-5	7B	128355197	128355220	AATTAAAGTGGGATGGATACTCCT	128355467	128355490	CACAATATAACGAAGAAGGTTTCC	I
G2chr17-6	7B	128355208	128355233	GATGGATACTCCTATACGTGTTATTG	128355471	128355495	AACAACACAATATAACGAAGAAGGT	IV
G2chr17-7	7B	128355257	128355276	TGCATCACAACAACACTTGC	128355466	128355491	ACACAATATAACGAAGAAGGTTTCCT	I
G2chr17-8	7B	129636057	129636080	TGTTTGATTAGTTGAGAACTCGTG	129636423	129636442	AACGACCACTGGGATCATTC	I
G2chr17-9	7B	131641786	131641810	CGGTCTTCAAAGTATCACTTCACTT	131641966	131641992	GGTCATTACCTATTAGTGATGTTTGCT	I
G2chr17-10	7B	133781624	133781646	TTGAAACGAATCAAACGATAACA	133781924	133781948	TGAGCAAGTATCAGACAAACAAAAG	I
G2chr18-1	8B	1677640	1677660	CGGTGGCTTGCAAAAATATAA	1678095	1678117	TTGTGCCTCATTAACAGAGATGA	IV
G2chr18-2	8B	3131141	3131163	GATTAACAGGCTGATTTTAATGC	3131557	3131579	CCAACAGACTAATTTCGGTTAAA	IV
G2chr18-3	8B	4430290	4430310	TGTGGCAAATTAAAGAAGACG	4430802	4430824	GAAATCGACCTATCAGAATTCAA	IV
G2chr18-4	8B	20098499	20098520	TTGGAGCTCTCGTAATTCCTTC	20098776	20098799	TTGCATTCGAATATAAATTTCAGC	I

G2chr18-5	8B	107442282	107442300	GCTGTGTTTGCGTTGGAGT	107442436	107442455	GATACGCGACCCCTAACATC	II
G2chr18-6	8B	110510444	110510468	CGTGAATTTTGTAAAGTTACTCCAA	110510594	110510615	TGTCCTTGATTATTTTGCTCCA	IV
G2chr18-7	8B	116874101	116874122	AAATCAGAAAAGTTGCTGTCCT	116874267	116874288	TCTGTAGTGGATGGTTGATCTG	IV
G2chr18-8	8B	128365967	128365986	ATAACGGCATAAACGCGACT	128366209	128366227	TTGCCGTCCGAGTTTATTG	I
G2chr18-9	8B	12818997	12819016	TTAAGGAATGGGCATCAAGG	12819552	12819573	TTAAGGGCCAAATAAACTAGGG	I
G2chr18-10	8B	52585612	52585635	TGGCATAATTTCTCCTATGAATTT	52585850	52585870	TCTCAGTGCCATCAATCAGAA	IV
G2chr19-1	9B	2292018	2292039	CCATACGCAAAATACATGGAGA	2292266	2292288	TGAGTAGCGATGAGCTTCAGTAA	I
G2chr19-2	9B	7987303	7987329	TGAAGTTCAAACATCAATTGGATATAG	7987606	7987629	AATCGATTTGATGTTCAATGTTGT	IV
G2chr19-3	9B	22601022	22601046	AAATAAATAACATGGTAAGCAAACG	22601417	22601441	GCACATGAATTGATCATTAACATAA	IV
G2chr19-4	9B	32692605	32692630	GGGATCACTCAAGTAATTGAACTAAG	32692846	32692866	CGTTGGATTAGAGCCATTTTC	IV
G2chr19-5	9B	110267100	110267119	CGAGCATGCGTTATTGTGTT	110267380	110267401	TTACAAGTCAAACGTGGTCTCC	I
G2chr19-6	9B	135872325	135872346	TGAGAGAAAGTTCGCAAAAGTG	135872711	135872733	CGACAAGTACCTGATATCGACAA	IV

G2chr19-7	9B	143953860	143953879	CACACGAAACCCATCTGCTA	143954185	143954204	TGAACACGAATGCAACCTTT	I
G2chr19-8	9B	154245437	154245461	AAAATCCTTAGCTTTGAGACTATGC	154245785	154245803	ATTCCTACCCCGCCCTTAT	IV
G2chr19-9	9B	155900998	155901017	ATGGCAACAGGAAGAAGCAT	155901232	155901251	GTGAAAGAAAAGGGCTGTGG	IV
G2chr19-10	9B	155904739	155904761	TTTGAACTGGTCTCTTTCTTTCC	155905183	155905202	CCTGCTCCACACCTTCCTAC	IV
G2chr20-1	10B	11512154	11512180	TCAGTCCATGCATACATAGATAGTACA	11512547	11512573	ACATTAAAAGACCTTGATTTGAAGTAT	IV
G2chr20-2	10B	116098628	116098648	CCCAAGATATGAAATGGAGGA	116099005	116099030	AGTCCATTTCAGGATTCTAGTAAAGA	I
G2chr20-3	10B	126383205	126383229	CAAATTAAACCATACATATCCCATT	126383526	126383545	ATGCACGTTTCGTCTTTTGA	IV
G2chr20-4	10B	127954815	127954840	TTAATCAGATTTTAGGTGTGATAACG	127955244	127955263	AGATAGCAATGGGGCAGGTA	IV
G2chr20-5	10B	143682979	143683001	AAATGAGATTACCAAAGGTCGTC	143683239	143683258	ATCATGTCTGCCTCGGAAAT	IV
G2chr20-6	10B	143910946	143910965	CACCCTAGCCATTAGGCAAT	143911462	143911486	TGTCTATTAAATTTACGGGTTTTGC	I
G2chr20-7	10B	143917504	143917529	CGTTTCTATTTTAACCATGATAACCA	143917801	143917820	GCTCCCTTCAAACAGAATCG	IV
G2chr20-8	10B	143917527	143917551	CCATGATAAACATGAGTGTTATTGA	143918013	143918036	AACAGATAAGAAACATCCGACTCA	I

G2chr20-9	10B	143925477	143925498	TGAGTTATTGACCACAGCACAA	143925906	143925924	GTGGTCCTGCAAGGTGAAG	I
G2chr20-10	10B	143927102	143927124	CCAATGTCTGTTGAGTGTTGACT	143927313	143927336	CGCAGTGTAAATTTAGATCCTACC	III
G3chr1-1	1A	93642731	93642750	GTATGGGAATGCATGGGAAT	93642988	93643006	CGCGTAAATGAAGCCCATA	IV
G3chr1-2	1A	97795628	97795647	TGCTCCTCCATGTTCATTTG	97795858	97795880	GGCTAATCTCACTGTGGTTTGTT	I
G3chr1-3	1A	12330419	12330443	TCCACAAATTTATCCAAATTAAACC	12330596	12330621	TCATCACAGTTAGTCAGCTACACATT	I
G3chr1-4	1A	28604021	28604043	CTGTCACAAGCTTTGAAGAGTCA	28604290	28604315	AAAGGCGATAACTAATTCAATCTTTT	IV
G3chr1-5	1A	95932250	95932273	TGATATTATCCCTTAATCCTGCTT	95932521	95932545	TGATCATGAACAGAGTTACTCAAAG	IV
G3chr1-6	1A	97795628	97795647	TGCTCCTCCATGTTCATTTG	97795858	97795880	GGCTAATCTCACTGTGGTTTGTT	I
G3chr1-7	1A	94768327	94768347	AGAGACGGGTACAATGGTGTT	94768725	94768744	GACTGACGAGAGGGCTCATT	IV
G3chr2-1	2A	1883150	1883173	TCAATTGATATGTCACCACTCAAA	1883263	1883289	CAAACTGATAGAATAAATTGCTTTCCT	I
G3chr2-2	2A	97954312	97954331	GCTGTTCATCTTGCCCATTT	97954538	97954557	CGGGGAAGCAAACATAAAAC	I
G3chr2-3	2A	11938116	11938142	AAAAATATGCTACATTAAAGCAGATCC	11938508	11938534	ATGGATGAAACATGGTAAGTAACTAAG	I
G3chr2-4	2A	13749900	13749920	CGACACTACCACTCGTCTCC	13750181	13750204	AATCAAACTGATTGGATTATT	I

				T			GGA	
G3chr2-5	2A	94527637	94527656	TCTTTTGTGGAATGCTTTGG	94527813	94527835	TGCTTTCACAAATTAAATCCAAA	IV
G3chr2-6	2A	6641846	6641865	CTCTCAACCGCCGTTACTTT	6642310	6642336	TTGAAGAAAACAGGCATATATGAGTTA	IV
G3chr3-1	3A	8829491	8829511	TTGTGTGCAAGTCCATTTCAA	8829610	8829629	TCCTCATTGAGTCTGCGTCA	I
G3chr3-2	3A	45387994	45388020	ATTCGAATTTAAATGTATTGTTAGAGG	45388267	45388286	GCATTGAAATCCAACCAGAA	I
G3chr3-3	3A	17169160	17169183	GACTCACTCCAGTTCATCTCTCAA	17169384	17169405	AGCAGACTAGCAGTGGTAGCAA	IV
G3chr3-4	3A	6450959	6450985	TTTGCTAAAGTAAAATCTTAACATGAA	6451322	6451341	TCGATATTTTGTCCGGGTTA	IV
G3chr3-5	3A	125773827	125773845	CCGAATCACTCAGGCTCAA	125774158	125774177	TTGTTGGAATGTTGGCATGT	IV
G3chr3-6	3A	137756077	137756096	TTGGAGGTGAAAGAGGAGGT	137756420	137756446	CGGGTACATAGTTAAATAGGATATGAA	IV
G3chr4-1	4A	11585302	11585321	CCAAAGTCCAAACATTGCAG	11585408	11585431	GGTTTAGCTGTTGTGTCATTTGTT	IV
G3chr4-2	4A	124876659	124876679	TGGTGGATATTTCATCCCTGT	124879647	124879671	CACGTTTTATATCTATCGCAGAGAA	I
G3chr4-3	4A	25749764	25749785	ACTTTGACGGAGAACTAATTGC	25750013	25750036	GGAAATCATTTTGAGTGTCTAAGC	IV
G3chr4-4	4A	117734439	117734460	TTGGGATTCAAGTTTAAGTGGT	117734679	117734702	TGTTCATAAGTAACCACGTATGCT	IV

G3chr4-5	4A	123413854	123413880	TTGGTGACTATACATTATATGGTTTGA	123414073	123414091	ATGCATGTTGAGCCCAAAG	I
G3chr4-6	4A	39240821	39240841	TTGGAAGATGATTTCATGTGG	39241009	39241030	AGCTCGACCACAAATACAAGAG	IV
G3chr4-7	4A	123770009	123770033	TGGCAGTGTTAAAGTCATAGGTAAA	123770204	123770224	TGAGCGCAATGAGAATAATGA	I
G3chr5-1	5A	11422657	11422679	AAAGGCTTGATTTTACCATACCC	11422866	11422889	CAATCATGTTATGGACACACAAAA	IV
G3chr5-2	5A	107668904	107668923	TAAATCTTCGAGGGGATGGA	107669103	107669121	GATCCCCATTCCAACCAAT	IV
G3chr5-3	5A	6708432	6708458	AGACTAGAACATTAGTTATGGGTTGAT	6708593	6708612	TGGAAGTGGGATTTTGTTTC	IV
G3chr5-4	5A	114057781	114057807	TTTGCAAACTCTGTCTAGTCTAATCAC	114058109	114058131	TTATGTAGACAAGCCACCACAAA	IV
G3chr5-5	5A	32689721	32689740	TTTTGCAATTCCTTCGATGA	32689971	32689996	GAAAGTCTACTATCTCCAGTTCGATG	IV
G3chr5-6	5A	56262125	56262145	CCATTCTTCTTGGGCTATTGA	56262290	56262314	TTCAAAGCAACCTAACAGTAACAGA	IV
G3chr5-7	5A	108605578	108605602	CAGTGTAAATTGTCAATCCTTTGTG	108605829	108605849	AAATGCGATCAGTACCCTCCT	IV
G3chr5-8	5A	114057492	114057513	CCAACATCATTCATCACAAACT	114057784	114057808	TGTGATTAGACTAGACAGAGTTTGC	IV
G3chr5-9	5A	12264056	12264082	GGACATGATTATTTGGTGTAACTATCT	12264349	12264367	ATGCAAAGCCCTTCACAAA	IV
G3chr5-1	5A	17479969	17479995	TGAAAACATCCTATCTATTTA	17480181	17480200	GGTTCCAAGCTTCTTTGCAT	IV

0				GGCACT				
G3chr5-11	5A	33633732	33633753	CTCTTCAGTGCTGACAAAATCA	33634020	33634042	AGCAGATTCAGAAGGAGATTGTT	I
G3chr5-12	5A	94198519	94198540	TTAAGTCTCGGGTTATTCATGC	94198853	94198873	CCGTTTAATTTGTTTGGCTTC	IV
G3chr5-13	5A	94969019	94969043	AAGTCTGTCTCTGAGATGAGAAATC	94969030	94969051	TTTTCATGACACAATGTCCAAG	I
G3chr5-14	5A	114057781	114057807	TTTGCAAACTCTGTCTAGTCTAATCAC	114058109	114058131	TTATGTAGACAAGCCACCACAAA	IV
G3chr6-1	6A	73002466	73002485	TGATTATAAGGGCGGGTCAG	73002654	73002676	CGGAGATTTTGAGGTTCTATTTG	I
G3chr6-2	6A	104032000	104032019	GGACTCTGGGGTTCTTGTGT	104055417	104055436	CTTTCACCAGTCACCACGTC	IV
G3chr6-3	6A	112403627	112403651	TCCTGTATGTGGACTTTCAGTAACA	112403967	112403990	TGAGGATGATCTTCTGCAATATCT	IV
G3chr6-4	6A	9841455	9841477	GGCTTTGTAGGCTTAGTAGCACA	9841651	9841677	TCATACCATCATTACAATCTTACATTT	I
G3chr6-5	6A	97858196	97858216	GGATGGGTAGTCCTTACCAAA	97858548	97858567	ACAACCTGTGATGTGGCAAT	IV
G3chr6-6	6A	36648622	36648643	TGTGTTAAAAGACCCTTGATGA	36648664	36648683	CCAAAAATTCCCATGGTCTT	IV
G3chr6-7	6A	18530547	18530569	TTCAATTAAGAGTGAGGGAAAGA	18530966	18530985	GTGGCGGTTGTTGTTATGAG	I
G3chr7-1	7A	2823828	2823848	AGCTAGTAACACAGGGCCAAT	2824071	2824095	CATCATCAAAGGAAAGTAATCATCA	IV

G3chr7-2	7A	76713700	76713723	TCACGTCTTTTATCTTTTGGATGA	76713837	76713857	TTGGGTTTTGAGGGACTAGAA	IV
G3chr7-3	7A	796822	796846	GAGGGTGACAAGATAGAAGGTAGTT	797095	797117	CAATGAGAGTAAATGGGAGTGTG	IV
G3chr7-4	7A	80533442	80533463	AGACCGGTAGTAGTGCGATACA	80533744	80533764	TGTGCTGTGTTTTGTGTCTGT	IV
G3chr7-5	7A	6318815	6318835	TGTGATAACCAGGGAAGTTGC	6319028	6319050	AGCACCAAGTTTGAAAAGAAGAC	I
G3chr7-6	7A	37267211	37267234	TCTCTTTTAAATTCACAACGGTAA	37267596	37267622	CAAGATATTGTAATGGTATGAATGTGT	IV
G3chr7-7	7A	72495476	72495499	ATTAATTCGATTATGTTGTTGTGC	72495934	72495953	GAAATGGCCACTTGCATCTA	I
G3chr7-8	7A	76848890	76848911	TGACACCAGCAATTGTGTTAAA	76849116	76849135	TTCGGCCTCATCTCTCTAGC	I
G3chr7-9	7A	2128684	2128707	GGTCTCGGGTTAGAACTTAGAAGA	2129005	2129024	GCGAAAACAAACATGCAAAA	IV
G3chr7-10	7A	71647121	71647143	AAGGCCTTTAATAGCACAAAAGA	71647468	71647489	TCTTCCATAATCAGTTCCAACC	IV
G3chr7-11	7A	79043441	79043464	GGATTATTTTATTTTGCAGAGTCG	79043647	79043666	GATGGATGGAGGGGTGTAGA	IV
G3chr8-1	8A	25225904	25225923	TTCTTGAGTTCACGGTGGTG	25226038	25226061	CCACAAACATTGAGCTTATGTACC	I
G3chr8-2	8A	37294920	37294941	CGTAGTTGACTAGCATTGTTGC	37295037	37295056	TTGCGCAATTCACCTAATTC	IV

G3chr8-3	8A	40843729	40843751	TTCCAATTGTTCATGAGATGAGA	40843918	40843939	AAATCTACTCCAAGGCTTTCCA	IV
G3chr8-4	8A	18079357	18079375	GCGACTTCGTGGTCCAGTA	18079607	18079632	TTTGTTAGAGTTATCGGAAACAACAT	IV
G3chr8-5	8A	25572743	25572765	AGACAAATGATGAAGATCAGAGG	25573098	25573119	TGGGAGTATTGGTTACTGTGAA	IV
G3chr8-6	8A	37138180	37138206	CGTACATTATTGGTTACTCACTTTCTC	37138401	37138420	GGGTTTGTTTGGCATTCTTT	I
G3chr8-7	8A	46521060	46521085	GATGTGAATACAAGAAGGCTATTTTT	46521538	46521561	GGTGTGAATGTTATTAGCATGGTT	IV
G3chr9-1	9A	20370225	20370246	TGATCATAGGCGTAATCACACA	20370320	20370344	GAGGAGATAGATATTGAGGATGGAA	I
G3chr9-2	9A	112025090	112025109	ACGAAAACCAACCGATAACC	112025316	112025337	AGGTCTCTGAGTTTCTCTGCAA	IV
G3chr9-3	9A	116509881	116509907	GTTTGTTACTAGATATTCAGGACCATT	116510283	116510302	AAGTTTGAGATGGCAACACG	IV
G3chr9-4	9A	12914807	12914828	CCTGACAAAGTTTTTAGCAGCA	12915048	12915070	TTTTCATTGTCACACTTTTGCTT	IV
G3chr9-5	9A	14234870	14234889	CAGAGAGAAGCAGGGAGGAG	14235090	14235112	TGCGAAGAAAGAAGACAATATCA	IV
G3chr9-6	9A	37850133	37850158	CAAAAGACTTATGACTTATGGAGCTG	37850479	37850499	TCCCTGAGTCTATTTGGTTCG	IV
G3chr9-7	9A	98110534	98110555	TCAAGAAAACTACATGGGCAAT	98110963	98110985	CACAAGAATCAATCCCTTAGCTT	I

G3chr10-1	10A	68745334	68745356	CGATCAACAATTTTCACATACCA	68745587	68745606	CCGTAACTCCCTAAGCATGG	IV
G3chr10-2	10A	113970785	113970808	AAAGAACTGAACTCTAGTGGGACA	113971037	113971059	TTCCAGAAATGATGCATAAACAA	IV
G3chr10-3	10A	6191701	6191727	AGCTAGCTATATTCAAATGATGTTTTC	6192034	6192057	TGGCAACGTATAATAGTAGTTGGA	I
G3chr10-4	10A	116321706	116321725	TTGAGCTGGGTTGACTTGAG	116321914	116321935	TCTTTCTATTCCCTTGCACAGT	IV
G3chr10-5	10A	18026571	18026591	TTTGGGGTTATCCTTAGCAGA	18026848	18026868	TATAATCTCACACCCGGCATA	IV
G3chr10-6	10A	68746250	68746269	AAGGCAAAATGGGGTATCAA	68746676	68746695	CCATTGCCTCACAAAATGAA	I
G3chr10-7	10A	80274197	80274216	CCTTTCCATATTCCGTTTGG	80274558	80274580	TTTCATGGTGTTCTAACTCAGCA	IV
G3chr10-8	10A	80278242	80278268	GCAATACTATGGTAGAAAGCATAGAGA	80278473	80278498	TTTCTCAATAAATTACCTTGATTTGG	IV
G3chr11-1	1B	6339513	6339538	CAACGCATAAGAATTAAACACAATTT	6339751	6339772	AACTCTGCTGTGTTGGTAGGAA	I
G3chr11-2	1B	146555759	146555778	ATCAATGAGCAGCAACAACG	146555997	146556016	GCAGGGGTGTTACGAGTCAT	IV
G3chr11-3	1B	18015521	18015547	TCTCTAGCATTACTCATTCATAAAACA	18015767	18015791	TGTTTGTCATATCATTTAGCTTTGA	I
G3chr11-4	1B	118678060	118678086	TTCATTATAGCTTATTAAATGCAAACA	118678269	118678288	TGTTGTCTGTTGTTGGGCTA	IV

G3chr11-5	1B	136266782	136266804	GAGGACTACTGCAACTCTTCAGG	136266982	136267004	AGCAAGGGAAGATGGTTTAGAGT	IV
G3chr11-6	1B	144116597	144116616	GGGCATCCAATCACTCACTT	144116919	144116938	GGTTGGACATTTGACCCTTC	I
G3chr12-1	2B	4800773	4800794	GCTTTTCACTCAATCACTCTGG	4800881	4800905	TGTGCAGTTTAACACATAACACAGA	I
G3chr12-2	2B	26269326	26269346	CAATGCAAAGAAGAGGAGGAA	26269574	26269598	GGAATCATCAACGTATTAATTTTGG	I
G3chr12-3	2B	8692897	8692922	TTGAACCTGTGTATTAGGATTTAGGA	8693266	8693283	GGGCTCTGCAGGACTCTG	IV
G3chr12-4	2B	13331030	13331055	CATCTTCAATTCATCCTAATCTTGTT	13331366	13331385	CCCCATGCATTCTATGTCCT	I
G3chr12-5	2B	33324147	33324169	TTGAAGAGATCAACAATTCAACG	33324546	33324565	AATGGGTACGGGTAGCAGTG	IV
G3chr12-6	2B	115196738	115196764	CGTATTAATATGAAGTATGATGACGTG	115197061	115197085	TGAATTTAATGCGAGTAACAAAGTT	IV
G3chr12-7	2B	2919662	2919681	TGCCCTACTCCTATGGGCTA	2920023	2920048	TGATCTGTTGATTTTACACTATGCTG	IV
G3chr12-8	2B	4404809	4404831	TTTCATGAATTCAAGTTTCAAGG	4405044	4405063	CCAGCTGACATTAGGGACAA	IV
G3chr12-9	2B	82037563	82037588	CACAACATACTTACACTCACACTGTC	82037919	82037939	TTGCTAGACGGAGTAGTGGTG	IV
G3chr13-1	3B	5853030	5853053	GAAGAAGACGAACAATTCTGACAA	5853175	5853194	ATTGTGTGGAGGGAATTTGC	IV

G3chr13-2	3B	143719309	143719334	AGTTTTATCTCATAGCAGTAGCACAC	143719420	143719442	TTTGAAGTAATAAAATGCGTTCG	IV
G3chr13-3	3B	710968	710988	TCGTTGGTGATTTTAAATGGA	711226	711251	CATTGTTGTTATCATATGAAGGAAAT	I
G3chr13-4	3B	8255725	8255746	CATTGCTCGGTGAATACAAGTT	8256062	8256081	CTTTTCAGGGTTTTGCCATT	I
G3chr13-5	3B	13853713	13853733	GCCAACCAAATAGAGAGGAAA	13854025	13854044	TCAATTTGGGGACCTAAACC	I
G3chr13-6	3B	24332602	24332627	CAATAATGGATTAGCACTAACATGAG	24332823	24332844	TCCATCGAGAACTCATTCTTTG	I
G3chr13-7	3B	47801205	47801228	GAAATTTGATGAACACATACATGC	47801577	47801596	CAAAATGCGAAATCCCAAGT	I
G3chr14-1	4B	7695307	7695329	CTTTGCTACAATGCAAGTTTCTT	7695534	7695560	TGAAATCATTAGAATTAAGGAAATGTG	IV
G3chr14-2	4B	137609105	137609125	TCATCAATTTCTTCAGCACCA	137609245	137609264	ATAACCAGCTGATGCAACCA	I
G3chr14-3	4B	6684393	6684417	TCAGAAGTATGATTAGAACAACCAA	6684669	6684688	AGAGCAGGATGTCATGGTTG	IV
G3chr14-4	4B	10949116	10949139	GTACTCTTTTGGTACGGAATTAGC	10949332	10949354	TGAGAGAAACGTTTACACTTGGA	IV
G3chr14-5	4B	17946543	17946568	AGGATCATTCGTGATAGTATGTTATG	17946798	17946818	GAACTCAATTATTGCGATGCT	IV
G3chr14-6	4B	30996151	30996171	GAGGAGGAGGAGGAAGAGTTG	30996344	30996370	TTTGGGTACCTTTAAAAGACTTATGAT	IV

G3chr14-7	4B	130887396	130887415	TGCACACATCTCCTCACTCC	130887686	130887707	TCTGATGAAGCTCTGATGAAGG	I
G3chr15-1	5B	9182277	9182299	GCAGGTTGAATTTGTATTTGAGG	9182448	9182470	AGAGCTCTGAAACGAGAGAATGA	I
G3chr15-2	5B	153839686	153839709	TTTTGTGAATTCTATATGCGCTTC	153839849	153839868	CCTGCATCAACCAAAACAGA	I
G3chr15-3	5B	8827074	8827100	CACGATCAAACATAAATAAGTAAGAAA	8827456	8827479	TTGGTGGAATTGTATTGTATTAGG	I
G3chr15-4	5B	22858021	22858047	TTGTAGGTGAATGACAGTTAGTTTTAG	22858361	22858385	GGTTTCATTTATTCTACTTCCATGA	I
G3chr15-5	5B	112042462	112042488	TGAAAGTAACTAATGGATGTGAATATG	112042712	112042731	TCTTTTGCGAACCAGTTGAA	I
G3chr15-6	5B	134450809	134450829	GGGAAGTTCTTCACCAGGTCT	134451216	134451242	ATTACAATGCAATCTACAACCTAATTT	IV
G3chr16-1	6B	21872585	21872604	AGCCAGATAGTGACCCCTCA	21872869	21872888	ACCTTGTTTGCTCCACCTGT	I
G3chr16-2	6B	142275794	142275820	GGACAACTACGTACTATTAGCACAAAC	142275907	142275928	GTTTGGTATTTGGCTCATGATT	IV
G3chr16-3	6B	16112425	16112451	TCAAACAGAAATATATCATGAGAAGAA	16112651	16112673	CAGATGGAGTGCTATTACGGTTA	IV
G3chr16-4	6B	20525349	20525370	CATATCACATTGCCTTTGGAGA	20525547	20525566	CAAGTGTGCATGGGGTATTG	IV
G3chr16-5	6B	26720119	26720139	CCACCGTATTCATCTTCATCA	26720477	26720498	TGAATACTTCCCCAGGGTATTT	I

G3chr16-6	6B	117206777	117206803	AAACATGTTAATATACAAATGGCTTCA	117207015	117207038	TGCTTTACATCAGTGTTCATACAA	IV
G3chr17-1	7B	7447076	7447098	AAAAAGATTTCTAGCCACCTGAA	7447313	7447333	GCATCCCTGTTCTTACGTGTT	I
G3chr17-2	7B	127744868	127744891	AACTCAAATAAGTGCTGCAAAGTT	127745034	127745054	TGAGTGGGATTTTAGCCGTAA	IV
G3chr17-3	7B	132250410	132250432	TTCTATTTCCCATTCACTCATCC	132250777	132250795	CAATTCGGAGCGGTACAGA	IV
G3chr17-4	7B	1417396	1417417	TGATGTTTGTACCTTGGAGGAG	1417739	1417760	GGACCATCATATACCAGGTCAA	IV
G3chr17-5	7B	13715441	13715466	GAATAGTCTTCTTTAACCAAACTCCA	13715729	13715748	AGTTAATTTGGGGGTGGTCA	IV
G3chr17-6	7B	22064827	22064852	CAAAATCAAAGCATTATAGACAAAGG	22065023	22065047	CTGAACTTACAAAGACACTTCCAAA	IV
G3chr17-7	7B	48872134	48872160	TCATCAGATTCATAATTCATCTACAAA	48872526	48872550	TCTAAGGATTAGGTTCTTGTTGTCA	IV
G3chr17-8	7B	2118798	2118817	TCCAAAATTGCTTCTTGCAC	2119095	2119114	GAAGGGTCGGCTTAGTAGGA	IV
G3chr17-9	7B	27271093	27271113	CCCCTAAGGTCTGTTTTACCC	27271313	27271334	CGCCTACAAAATTTGAGACATC	IV
G3chr17-10	7B	28816781	28816804	CATGCACATTATCTTTGACTTTGA	28817165	28817186	TGGTGTCTGTAAACCTCTGAGC	IV
G3chr18-1	8B	4552488	4552507	ACCGTCTCGTCTCTTGTCAC	4552684	4552706	TCTTGTGTATTGGTTTGTTTTCC	I

G3chr18-2	8B	120916178	120916197	CTGTCCTGTCAGCCTCCTTC	120916386	120916407	ACGCCCTTCGATATATTTCTCT	I
G3chr18-3	8B	3613086	3613106	CATGACAGAGAAATGGTCCAA	3613371	3613391	TGGATCTTTTAGATGGCGTTT	IV
G3chr18-4	8B	12097642	12097668	TGTTCTGATTTTAATATAGGTGTTTGC	12097900	12097924	AAACATCTTCACATGAGTAGTCACC	IV
G3chr18-5	8B	23368873	23368894	GGAACAGCAAGTACCATTTTCA	23369142	23369165	GAGAAGTATTTGATGGATCATGGA	I
G3chr18-6	8B	32495043	32495061	GGATGCAGGATGCTCTCAA	32495307	32495326	TCCTGACCAACATTGGCATA	I
G3chr19-1	9B	8277160	8277181	GAAGGGTGACAAATTAAATGGA	8277317	8277340	AACATTCTGATAAACCGACATACA	I
G3chr19-2	9B	147696105	147696124	TTGGTTTTCTAATCGCTTGC	147696382	147696405	TTGGTAATTTATATGGGTTCGATT	I
G3chr19-3	9B	157836620	157836640	TTAGCATAACCGTTGACATGC	157836970	157836991	CAAGAAGTTTTCCAATCCCATA	IV
G3chr19-4	9B	258215	258238	AACAACTAACAGGTGATGATTGGA	258483	258506	TCAGTAACTCGGTGAATATCCGTA	IV
G3chr19-5	9B	11446976	11446994	ATCACGCTGAAGGCACAAC	11447228	11447252	GCGTTGGTTTAGTTAATGTATTGGT	I
G3chr19-6	9B	51491558	51491580	TCACGCTAAAGCATCTCTATCTC	51491777	51491801	TTTAGGATATGAATTGCTTTCTTTC	I
G3chr19-7	9B	125958648	125958667	GGCAATTTCACAGCTTCCTC	125958976	125958997	CACTTACCAACAGAGTGGGAGA	IV

G3chr20-1	10B	2074299	2074318	GAGTCACCAGTCAGCACCAA	2074502	2074525	TTGATTTTTCATTTGCTAAGCACT	I
G3chr20-2	10B	107812990	107813012	TTGACTTCTCAACCATGTCATCT	107813140	107813159	ATCAACGCAACCAAAGCATA	I
G3chr20-3	10B	7202570	7202595	CCTAATCTTGACAAAGAAATTTATCC	7202791	7202810	CGGTTGGTGATTTCTGTTCT	IV
G3chr20-4	10B	18027409	18027432	TGGCTGTGAAGAATTATAATGTGA	18027738	18027757	TGGCCTGTTTTACCTCAATG	I
G3chr20-5	10B	35787928	35787952	TTCCATCCTAATGTTAGAGTGTTTT	35788133	35788158	CATAATCGGTAAGTATCTTGAAACAG	I
G3chr20-6	10B	126368422	126368444	TTGTAGCCTCAATTTGCTACTCC	126368626	126368652	TTTGTATTATTCATAGAAGCCAAACTT	IV
G3chr20-7	10B	140696401	140696421	AGAAAGCCAACGAAAAAGAAG	140696732	140696751	TCATTTGGTAGGGTTGGCTA	IV

NOTE:Type I, Wild peanut accessions have electrophoretic bands, while cultivated peanut does not have electrophoretic bands; Type II, Wild peanut accessions do not have electrophoretic bands, while cultivated peanut has electrophoretic bands; Type III, The electrophoretic bands of wild peanut species and cultivated peanuts are at different migration rates; Type IV, Both wild peanut species and cultivated peanuts lack electrophoretic bands, or the bands are at the same migration rate.
